# Global genomic epidemiology of chromosomally mediated non-enzymatic carbapenem resistance in *Acinetobacter baumannii*: on the way to predict and modify resistance

**DOI:** 10.3389/fmicb.2023.1271733

**Published:** 2023-10-06

**Authors:** Wedad M. Nageeb, Nada AlHarbi, Amani A. Alrehaili, Shadi A. Zakai, Ahmed Elfadadny, Helal F. Hetta

**Affiliations:** ^1^Department of Medical Microbiology and Immunology, Faculty of Medicine, Suez Canal University, Ismailia, Egypt; ^2^Department of Biology, College of Science, Princess Nourah bint Abdulrahman University, Riyadh, Saudi Arabia; ^3^Department of Clinical Laboratory Sciences, College of Applied Medical Sciences, Taif University, Taif, Saudi Arabia; ^4^Department of Clinical Microbiology and Immunology, Faculty of Medicine, King Abdulaziz University, Jeddah, Saudi Arabia; ^5^Department of Animal Internal Medicine, Faculty of Veterinary Medicine, Damanhour University, Damanhour, El-Beheira, Egypt; ^6^Department of Medical Microbiology and Immunology, Faculty of Medicine, Assiut University, Assiut, Egypt

**Keywords:** *Acinetobacter baumannii*, carbapenem resistance, multidrug efflux pump, penicillin binding proteins, new drug targets, molecular predictors, diagnostic markers

## Abstract

**Introduction:**

Although carbapenemases are frequently reported in resistant *A. baumannii* clinical isolates, other chromosomally mediated elements of resistance that are considered essential are frequently underestimated. Having a wide substrate range, multidrug efflux pumps frequently underlie antibiotic treatment failure. Recognizing and exploiting variations in multidrug efflux pumps and penicillin-binding proteins (PBPs) is an essential approach in new antibiotic drug discovery and engineering to meet the growing challenge of multidrug-resistant Gram-negative bacteria.

**Methods:**

A total of 980 whole genome sequences of *A. baumannii* were analyzed. Nucleotide sequences for the genes studied were queried against a custom database of FASTA sequences using the Bacterial and Viral Bioinformatics Resource Center (BV-BRC) system. The correlation between different variants and carbapenem Minimum Inhibitory Concentrations (MICs) was studied. PROVEAN and I-Mutant predictor suites were used to predict the effect of the studied amino acid substitutions on protein function and protein stability. Both PsiPred and FUpred were used for domain and secondary structure prediction. Phylogenetic reconstruction was performed using SANS serif and then visualized using iTOL and Phandango.

**Results:**

Exhibiting the highest detection rate, *AdeB* codes for an important efflux-pump structural protein. T48V, T584I, and P660Q were important variants identified in the *AdeB*-predicted multidrug efflux transporter pore domains. These can act as probable targets for designing new efflux-pump inhibitors. Each of *AdeC* Q239L and *AdeS* D167N can also act as probable targets for restoring carbapenem susceptibility. Membrane proteins appear to have lower predictive potential than efflux pump-related changes. OprB and OprD changes show a greater effect than OmpA, OmpW, Omp33, and CarO changes on carbapenem susceptibility. Functional and statistical evidence make the variants T636A and S382N at PBP1a good markers for imipenem susceptibility and potential important drug targets that can modify imipenem resistance. In addition, PBP3_370, PBP1a_T636A, and PBP1a_S382N may act as potential drug targets that can be exploited to counteract imipenem resistance.

**Conclusion:**

The study presents a comprehensive epidemiologic and statistical analysis of potential membrane proteins and efflux-pump variants related to carbapenem susceptibility in *A. baumannii*, shedding light on their clinical utility as diagnostic markers and treatment modification targets for more focused studies of candidate elements.

## 1. Introduction

Although initially described as an opportunistic bacterium of low pathogenicity, *A. baumannii* has emerged as an important pathogen in critical care settings during the last decades. It is known to be widely involved in diseases such as mechanical ventilator-associated pneumonia, osteomyelitis, peritonitis, endocarditis, septicemia, and meningitis, as well as wound, skin, soft tissue, urinary tract, ear, and eye infections (Ayoub Moubareck and Hammoudi Halat, 2020). Additionally, outbreaks caused by *A. baumannii* are common in intensive care units (ICUs) and burn units (Lima et al., 2019).

Especially concerning is the emergence of multidrug resistant (MDR) *A. baumannii* and its alarming spread all over the world. Their emergence is usually associated with prolonged hospitalizations and increased morbidity and mortality, particularly in ICU patients. The incidence of MDR *A. baumannii* has been associated with some underlying risk factors, including burn infections and premature births, as well as the use of mechanical ventilation, indwelling devices, and extensive exposure to antimicrobial therapy (Agyepong et al., 2023).

Carbapenems are extremely important for controlling severe Gram-negative infections, particularly those caused by multidrug-resistant bacteria. Carbapenem antibiotics, such as imipenem, meropenem, and doripenem but not ertapenem, are considered last-resort treatments for MDR *A. baumannii* infection (Vila and Pachón, 2012; Nguyen and Joshi, 2021). Both imipenem and meropenem are used as empiric therapy for a variety of severe Gram-negative infections. However, since 1991, when the first carbapenem-resistant *A. baumannii* (CRAB) was recognized, a considerable increase in the number of these strains has been documented worldwide (Peleg et al., 2008; Gales et al., 2019; ECDC, 2022). Although carbapenem is one of the last-resort treatments, hospital outbreaks caused by CRAB isolates have been described earlier worldwide (Durante-Mangoni and Zarrilli, 2011; Migliavacca et al., 2013). This also includes high-resistance levels in burn units (Shoja et al., 2017; Lima et al., 2019). Evidence shows that patients infected with CRAB exhibit significantly higher mortality than those infected with carbapenem-susceptible *A. baumannii* (Lee et al., 2012). The rate of mortality related to CRAB infection was reported at 45.4%. Ventilator-associated pneumonia and bloodstream infections were the most prevalent infections in these cases (Schimith Bier et al., 2010; Kanafani et al., 2018; Gu et al., 2021). With a strong capacity for clonal transmission and acquirement of antimicrobial resistance determinants, in 2017, the World Health Organization (WHO) announced CRAB as the top-priority pathogen, critically requiring research and development of new antibiotics (Tacconelli and Magrini, 2017).

Mechanisms of carbapenem resistance in *A. baumannii* can be classified into enzymatic mechanisms, which involve the production of different carbapenemases and non-enzymatic mechanisms. The latter includes genetic alterations of PBPs, overexpression of efflux pumps belonging to the resistance-nodulation-cell division (RND) family, and the loss or modifications of outer membrane porins (Nguyen and Joshi, 2021; Vahhabi et al., 2021).

Efflux pumps such as *AdeABC* are reportedly involved in multidrug resistance (Vila et al., 2007; Xu et al., 2019). *AdeABC*, a resistance-nodulation-division (RND) family-type pump, has three component structures, *AdeA, AdeB*, and *AdeC*, which form the inner membrane fusion protein, the transmembrane component, and the Outer Membrane Protein (OMP), respectively. The *AdeABC* operon found in 80% of clinical isolates is chromosomally encoded and normally regulated by a two-component system, including the sensor kinase gene (*AdeS*) and its related response regulator gene (*AdeR*). Overexpression of *AdeB* has been attributed to mutations in the *AdeS* and *AdeR* genes and also to the insertion of ISAba1 into the *AdeS* gene or upstream from the operon, resulting in multidrug resistance in *A. baumannii* (Marchand et al., 2004; Coyne et al., 2011). Studies have demonstrated that the *AdeB* gene shows 10–40 times higher levels of expression in resistant isolates than in carbapenem-susceptible isolates, suggesting the association of *AdeABC* efflux pump with carbapenem resistance (Lee et al., 2010). It has been suggested that the *AdeABC* genes are common in *A. baumannii* but may be absent in fully susceptible isolates (Nemec et al., 2007). The *AdeB* gene, especially when hyperproduced, has been previously suggested as a genetic marker of MDR AB clinical isolates (Huys et al., 2005; Huang et al., 2008). Other efflux pumps include *AdeIJK* and a third RND efflux pump, *AdeFGH*, which confers multidrug resistance when overexpressed (Xu et al., 2019). Mutations in the upstream-located transcriptional regulator *AdeL* have been linked to *AdeFGH* overexpression (Coyne et al., 2010; Leus et al., 2020).

Next to antibiotic efflux, PBP modifications and changes in outer membrane proteins are among the most important non-enzymatic mechanisms of carbapenem resistance. PBP modifications are considered important mechanisms of chromosomally mediated acquired carbapenem resistance; however, they are less frequently reported (Poirel and Nordmann, 2006; Martínez-Trejo et al., 2022). It has been proposed that the loss or disruption of small outer membrane proteins would cause carbapenem resistance. This suggests their role as influx channels for carbapenem; however, carbapenem-binding sites have not been fully identified in all these types of membrane porins (Vahhabi et al., 2021).

Although both carbapenemase production and outer membrane permeability have been proposed to be jointly responsible for high levels of carbapenem resistance (Poirel and Nordmann, 2006; Nguyen and Joshi, 2021), investigators have associated imipenem-resistant clinical isolates of *A. baumannii*, which exhibited no carbapenemase activity, with the loss of outer membrane proteins (Limansky et al., 2002; Uppalapati et al., 2020). Other previous studies have also shown that high-level carbapenem resistance could result from the loss of outer membrane porins with the absence of carbapenemase activity (del Mar Tomás et al., 2005). In addition, there is evidence to suggest that clinical resistance to carbapenem develops during treatment in relation to a 4.6-fold increase in *AdeB* gene expression, while carbapenemase genes remain negative (Yamada and Suwabe, 2013). The same study also reported a loss of CarO resulting in carbapenem resistance with the absence of any carbapenemase activity. Experimental evidence has also shown that increased carbapenem MICs occur solely because of the overexpression of efflux pumps in the absence of carbapenemase activity or the overexpression of structural genes for the enzymes (Yoon et al., 2015). In addition, the overexpression of *AdeABC* combined with enzymatic resistance has been shown to contribute to carbapenem resistance in more than an additive manner, with a good relationship demonstrated between the level of expression and the level of resistance (Yoon et al., 2015).

The modification of PBPs as a carbapenem-resistance mechanism also appears to be under-reported. Carbapenem-resistant-derived *in-vitro* mutants exhibited hyperproduction of seven types of PBPs (Gehrlein et al., 1991), while other imipenem-resistant isolates exhibited an absence of 73.2-kDa PBP, besides carbapenemase production (Fernández-Cuenca et al., 2003). Both impermeability of the outer membrane mediated by different membrane porins and efflux-pump activity are important intrinsic determinants of resistance in Gram-negative bacteria, specifically in *A. baumannii* surviving in harsh hospital environments, and this necessitates a thorough and detailed study of such determinants (Bazyleu and Kumar, 2014).

Although carbapenemase-mediated resistance is studied extensively (Beigverdi et al., 2019; Strateva et al., 2019), its exact contribution to carbapenem resistance remains uncertain (Lee et al., 2012). In addition, the overall contribution of other non-enzymatic mechanisms involved in carbapenem resistance, including reduced outer membrane permeability, penicillin-binding protein alterations, and efflux pumps' overexpression, appears to be less well-studied (Lin, 2017). Carbapenem resistance can be encountered in a wide range of diverse genetic backgrounds. This study focused on determining the genetic structure and examining different membrane-related elements and their role in carbapenem resistance, including alterations in outer membrane porins, PBPs, and efflux-pump changes. This can prove especially valuable in cases of resistance emerging in prolonged treatments in critical settings.

In contrast to carbapenemases, most outer membrane proteins and PBPs are integral parts of the cell and are considered structurally and physiologically indispensable. In addition, it has been suggested that efflux pumps such as *AdeABC* and *AdeIJK* are tightly integrated into *A. baumannii* physiology and play distinct physiological roles related to cellular growth and survival (Leus et al., 2020). Under such circumstances, identifying determinants and markers of susceptibility among such a group of chromosomal cellular components would serve as candidate stable diagnostic markers.

This research aims to study the diversity in membrane genes contributing to carbapenem resistance and to determine the repertoire and phylogeny of resistance determinants among timely and geographically spaced clinical isolates of *A. baumannii*. A complete understanding of the mechanisms contributing to resistance phenotypes can be used as a target for novel drug development. Consequently, determining the contribution of different resistance mechanisms can aid in screening for new antimicrobial targets by prioritizing the most effective treatment targets.

In this study, we highlight the importance of these chromosomally encoded elements in determining carbapenem resistance. Our aim is to exploit this knowledge to design diagnostic markers that can guide the selection of the best treatment options, ultimately reducing the emergence of resistance. In addition, understanding the mutations that affect efflux-pump function could help design improved active efflux-pump inhibitors.

## 2. Methods

### 2.1. Genomes and genes studied

A total of 980 whole genome sequences of *A. baumannii* available at the NCBI and the PATRIC databases (Wattam et al., 2017) currently integrated into the Bacterial and Viral Bioinformatics Resource Center (BV-BRC) (Olson et al., 2023) were studied. The studied genome IDs are shown in Supplementary Table S1. *A. baumannii* genomic FASTA files were downloaded from the PATRIC database with their corresponding imipenem and meropenem MIC data. Nucleotide sequences for the genes were retrieved from the NCBI gene bank and queried using MegaBlast against a custom database of FASTA sequences on the BV-BRC system using BLASTn to search the nucleotide database using a nucleotide query sequence for each of the following entries: multidrug efflux RND transporter periplasmic adaptor subunit *AdeA* (NCBI Reference Sequence: NZ_CP009257.1: c3978859-3977669), multidrug efflux RND transporter permease subunits *AdeB* (NZ_CP009257.1: c3977672-3974562) and *AdeC* (NZ_CP009257.1:c3974485-3973088), efflux system response regulator transcription factor *AdeR* (NZ_CP009257.1:3979005-3979748), two-component sensor histidine kinase *AdeS* (NZ_CP009257.1:3979780-3980853), multidrug efflux RND transporter periplasmic adaptor subunit *AdeF* (NZ_CP009257.1:1450871-1452091), multidrug efflux RND transporter permease subunit *AdeG* (NZ_CP009257.1:1452098-1455277), multidrug efflux RND transporter outer membrane subunit *AdeH* (NZ_CP009257.1:1455290-1456738), multidrug efflux transcriptional repressor *AdeL* (NZ_CP009257.1:c1450673-1449643), multidrug efflux RND transporter periplasmic adaptor subunit *AdeI* (NZ_CP009257.1:1990739-1991989), multidrug efflux RND transporter permease subunit *AdeJ* (NZ_CP009257.1:1992002-1995178), multidrug efflux RND transporter outer membrane channel subunit *AdeK* (NZ_CP009257.1:1995178-1996632), and multidrug efflux transcriptional repressor *AdeN* (NZ_CP009257.1:4265008-4265661).

tBLASTn was used to search the translated nucleotide database using a protein query sequence for each of the carbapenem-associated resistance proteins, CarO1, CarO2, and CarO3 (with protein data bank accession codes 4RL9, 4RLB, and 4FUV). tBLASTn was also used to query the outer membrane protein A (GenBank: ATG88079.1), OmpW (NCBI Reference Sequence: WP_004712981.1), Omp33-36 (NCBI Reference Sequence: WP_000733003.1), glucose-selective porin OprB (GenBank: BAP65421.1), OprD outer membrane porin (NCBI Reference Sequence: WP_002059892.1), and a group of penicillin-binding proteins, including PBP1a MrcA membrane carboxypeptidase (NCBI Reference Sequence: WP_000736677.1), PBP1b (NCBI Reference Sequence: WP_000667420.1), PBP2_*mrd*A (NCBI Reference Sequence: WP_000809155.1), PBP3 FtsI (NCBI Reference Sequence: WP_000227939.1), PBP6 *dacC* (GenBank: AEK25710.1), PBP6b D-alanyl-D-alanine carboxypeptidase (NCBI Reference Sequence: WP_000667917.1), PBP7/8 *pbpG* D-alanyl-D-alanine endopeptidase (NCBI Reference Sequence: WP_001984577.1), and *MtgA* monofunctional biosynthetic peptidoglycan transglycosylase (NCBI Reference Sequence: WP_000642942.1).

Genes included in the analysis had >98% sequence identity and >25% query coverage OR > 90% sequence identity and >50% query coverage. The protein BLAST output or the translated protein sequence for each gene locus in each strain was then aligned using the MUSCLE option, following which it was visualized and comprehensively explored for different amino acid substitutions using the MEGA software. Gene variants identified for membrane protein genes were blasted using tBLASTn to blast the variant protein sequences against a custom-built nucleotide database using BV-BRC tools and services (Wattam et al., 2017; Olson et al., 2023).

### 2.2. Identification and analysis of chromosomal-gene variants

A literature search was performed on each of the databases of PMC PubMed, ACADEMIC SEARCH COMPLETE (EBSCO host), Scopus, and Web of Science core collection using the following search criteria: “carbapenem resistant *Acinetobacter baumannii*” [title/abstract/keywords] or [topic] AND “resistance mechanisms” [title/abstract/keywords] or [topic]. A search was also performed to include abbreviations such as “carbapenem resistant *A. baumannii*” or “CRAB.” Additional, more detailed searches were performed using the following search criteria: “*Acinetobacter baumannii*” or “*A. baumannii*” AND “membrane proteins” and also “*Acinetobacter baumannii*” or “*A. baumannii*” AND “efflux pumps” to extract all previously identified variants in the studied genes. A summary of output variants previously mentioned in the literature is listed in Supplementary Table S2. These variants, in addition to other variants identified during the current analysis, were studied in further detail. A matrix was built to study the distribution of different variants and their correlation to carbapenem MIC in the agents studied. Predictive values of their performance as predictors for carbapenem resistance and their distribution in relation to genetic background relatedness were studied. This was performed by classifying the sequences under study into a susceptible group and a non-susceptible group based on MIC values at the cutoff value of 2, according to the latest EUCAST breakpoint tables (EUCAST, 2023). The significance of gene variants among the two groups was determined by conducting a chi-squared test for independence, and their predictive values toward the outcome of interest (susceptibility/lack of susceptibility) were determined as previously indicated (Nageeb and Hetta, 2022).

### 2.3. Identification of the functional and phylogenetic context of analyzed variants

These measures were evaluated in relation to their genetic background distribution and predicted functional effects to filter out the most probable significant phenotype predictors. PROVEAN (Choi and Chan, 2015) and I-Mutant predictor suite (Capriotti et al., 2005) were used to predict the effect of the studied amino acid substitutions on protein function and protein stability, respectively.

Both PsiPred (McGuffin et al., 2000) available at http://bioinf.cs.ucl.ac.uk/psipred/ and FUpred (Zheng et al., 2020) available at https://zhanggroup.org/FUpred/ were used for domain and secondary structure prediction for each of the studied proteins. The CATH/Gene 3D v4.3 (Knudsen and Wiuf, 2010) database available at https://www.cathdb.info/ was searched using FASTA protein sequences to locate the position of structural domains on each efflux pump's protein sequence. UniProt (Consortium, 2019) was also searched for protein features and predicted structures using AlphaFold (Jumper et al., 2021) when available. Variants were mapped and visualized on the Protein 3D structure using BIOVIA Discovery Studio Visualizer v21.1.0.20298, Release 2020, San Diego: Dassault Systèmes and The PyMOL Molecular Graphics System, Version 2, Schrödinger, LLC.

To investigate the genetic relatedness of the studied sequences, whole genome-based phylogenetic reconstruction was performed using SANS serif (Rempel and Wittler, 2021). The length of K-mers was set to the default value of 31, and the assembled genomes' FASTA files were used as the input. SANS serif was used with the option “strict” to generate an output file in the Newick format, which was then visualized using iTOLv6 (Letunic and Bork, 2021). Phandango interactive visualization (Hadfield et al., 2018) was used to plot the variant distribution and other metadata in relation to the genetic relatedness phylogram.

## 3. Results

### 3.1. Epidemiology and diversity of the studied *A. baumannii* genome sequences

In this study, we report the distribution of the structural and regulatory genes encoding different efflux pump systems among a large group of studied whole genome sequences of *A. baumannii* and their correlation to carbapenem resistance. The studied sequences included a total of 980 whole genome sequences isolated from different geographic localities, including United States (Alaska, Washington, and Maryland), China (Shandong, Liaoning, Zhejiang, Beijing, Guangdong, Jiangsu, Shanghai, Tianjin, and Hunan), South Korea (Seoul), Thailand, Sweden, Germany, Italy, Iraq, Colombia, United Kingdom, Afghanistan, Honduras, Peru, and India. The studied sequences were isolated from different body sources, including sputum, blood, abdominal fluid, tracheal mucosa, tissue, skin, feces, rectum, urine, and wound, and spanned a range of isolation years between 1982 and 2017. They included 423 imipenem-resistant, 557 imipenem-susceptible, 354 meropenem-resistant, and 208 meropenem-susceptible isolates.

In this study, we examined the molecular epidemiology and the predictive values of different membrane-related elements in relation to carbapenem resistance, including efflux-pump proteins, and we performed a more detailed analysis of the distribution of different variants related to these groups of genes. We aimed to explore the repertoire, predictive capacity, and phylogeny of these studied resistance determinants among a diverse group of clinical isolates.

**Figure 1:**
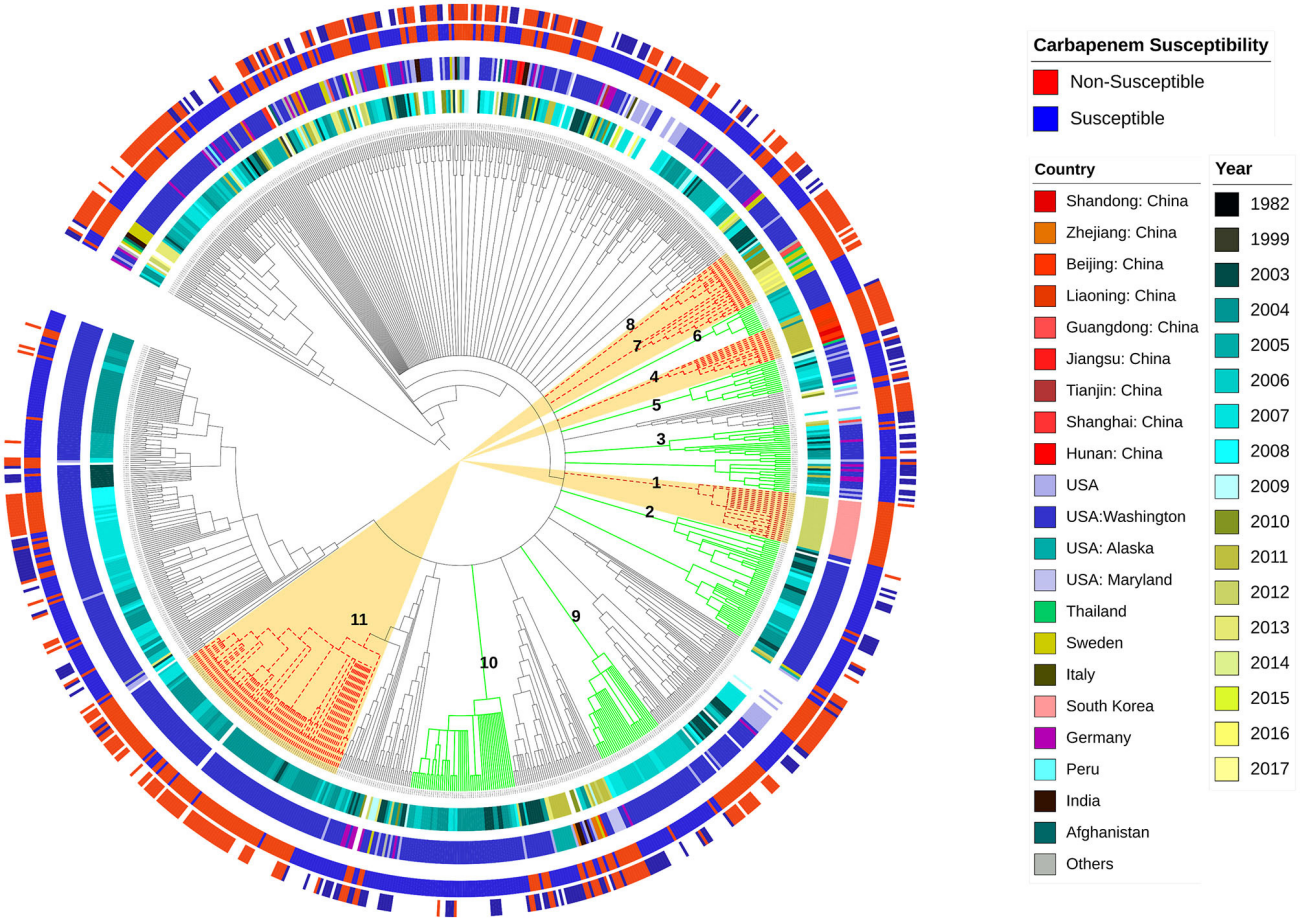
Whole genome-based genetic-relatedness phylogram and a heat map illustrating the correlation of the genetic background with the sequences' phenotype, their genome country of observation, and the year of isolation. Inner circle 1 shows different years of isolation. The second circle shows the country of isolation. The third circle represents the imipenem-susceptibility phenotype, while the fourth circle represents the meropenem-susceptibility phenotype.

Figure 1 illustrates the diversity of the studied sequences and shows the distribution of their phenotypes of imipenem and meropenem resistance, their geographic location, and the year of collection in relation to genetic background relatedness. From the figure, it can be observed that the studied sequences are genetically diverse with a heterogeneous distribution of resistance phenotypes in relation to the genetic background, country, and time of collection. Cluster 1 shows imipenem-resistant sequences, which were isolated from South Korea, and this cluster was located between two other carbapenem-sensitive clusters (Clusters 2 and 3) in the phylogram genetic order. Cluster 2 shows both imipenem- and meropenem-susceptible sequences that were collected in Washington, D.C. (USA) between 2003 and 2008. Similarly, Cluster 3 also contained carbapenem-susceptible sequences isolated from Germany and the USA, mostly between 2005 and 2009. Cluster 4 contained sequences that are resistant to both imipenem and meropenem collected from three different localities in China (Shandong, Liaoning, and Beijing), mostly during 2011, and this cluster was related to two other susceptible clusters (Clusters 5 and 6). Cluster 5 contained sequences that are susceptible to both imipenem and meropenem, mostly isolated in the USA between 2003 and 2009, while Cluster 6 contained carbapenem-susceptible isolates that were also isolated in the USA between 2005 and 2006. Both Clusters 7 and 8 showed closely related imipenem and meropenem non-susceptible sequences isolated from different countries, including Thailand, Sweden, USA, and China, with Cluster 7 being isolated between 2011 and 2016 and Cluster 8 between 2003 and 2010. Other examples showing the diverse distribution of the current analyzed set of sequences are Clusters 9 and 10. Both clusters contained imipenem-susceptible isolates that were isolated in the USA between 2006 and 2007 and between 2003 and 2008, respectively. The sequences from Cluster 11 contained imipenem- and meropenem-resistant sequences that were also isolated in the USA between 2003 and 2007.

### 3.2. Distribution of efflux pump and efflux-pump regulator genes and related variants in *A. baumannii*

*AdeABC* is known to be regulated by the *AdeRS* two-component regulatory system, *AdeFGH* by the LysR-type transcriptional regulator *AdeL*, and *AdeIJK* by the TetR-transcriptional regulator *AdeN*. Exploring these genes in the current analyzed set of sequences showed that *AdeA* occurred at the frequency of 94.18%, *AdeB* at the frequency of 95.61%, *AdeC* at the frequency of 68.57%, *AdeR* at the frequency of 92.85%, and *AdeS* at the frequency of 93.46%.

The *AdeA* gene was absent in 55 sequences, with two other sequences not meeting the identification criteria. The *AdeB* was absent in 30 sequences, with 13 other sequences not meeting the identification criteria. The *AdeC* was absent in 305 sequences, with three other sequences not meeting the identification criteria. *AdeR* was absent in 68 sequences, with two other sequences not meeting the identification criteria. The *AdeS* gene was absent in 62 sequences, with two other sequences not meeting the identification criteria. Both *AdeF* and *AdeG* were absent in six sequences, with one other sequence not meeting the identification criteria in both genes, while *AdeH* was also absent in six sequences, with two other sequences not meeting the identification criteria. The *AdeL* was absent in six sequences, while both *AdeI* and *AdeN* were absent in five sequences. The *AdeJ* was absent in four sequences, while *AdeK* was absent in four sequences, with two other sequences not meeting the identification criteria.

**Figure 2:**
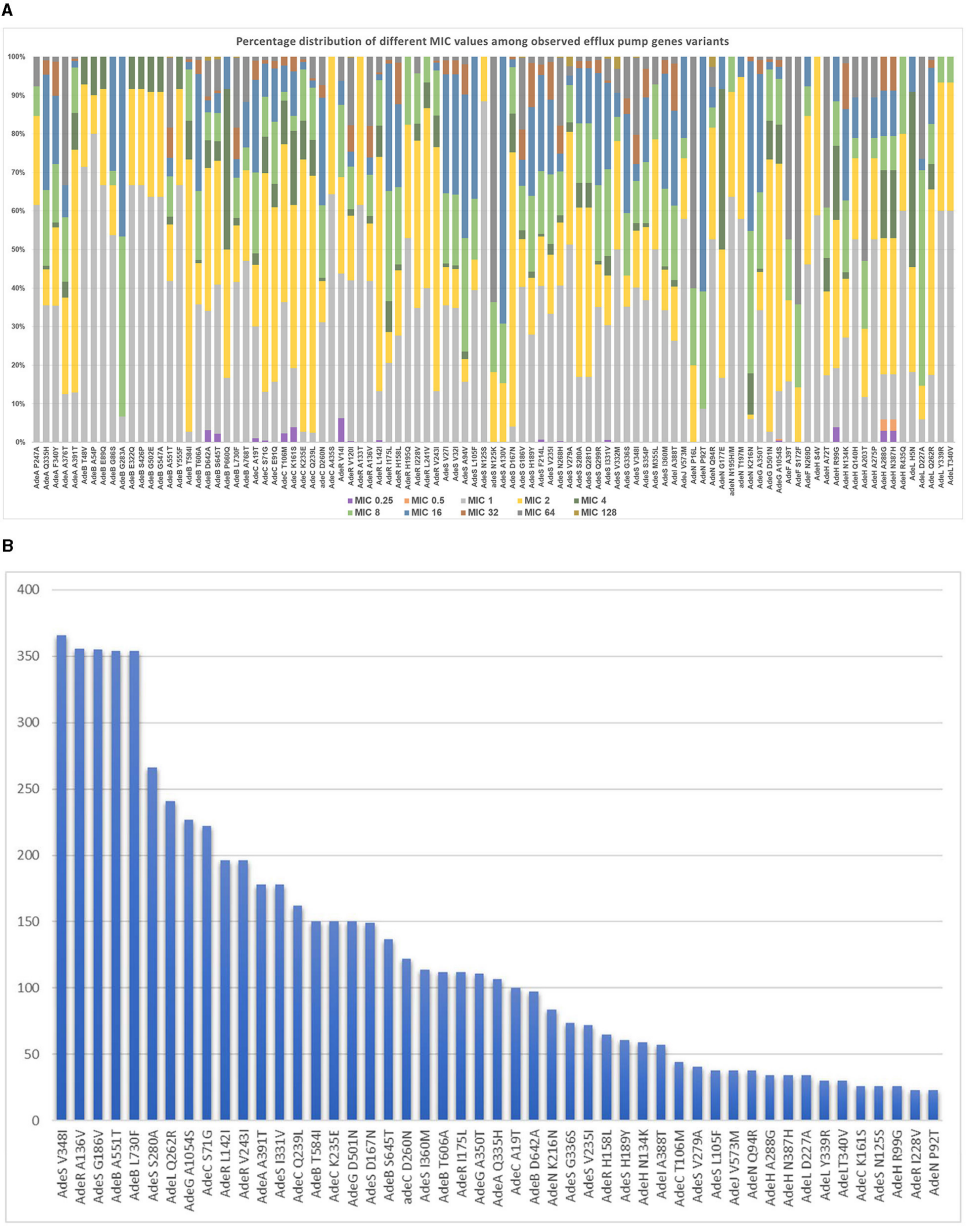
**(A)** Frequency of observation of significant efflux pump gene variants in relation to different MIC levels. **(B)** Frequency of observation of the most frequently occurring significant efflux-pump gene variants.

The analyzed structural and regulatory variants, related to the three studied efflux pump systems included a total of 117 variants, including the analysis of variants previously reported in the literature (shown in Supplementary Table S2) and other new variants analyzed in the current study. The percentage distribution of 97 variants occurring at more than 10 sequences among different imipenem MIC levels is shown in Figure 2A. Figure 2A illustrates a frequency of observation of significant efflux-pump gene variants in relation to different imipenem MIC levels. The frequency of significant efflux-pump variants occurring at more than 20 sequences is shown in Figure 2B in the descending order of frequency of occurrence among the entire studied set.

**Figure 3:**
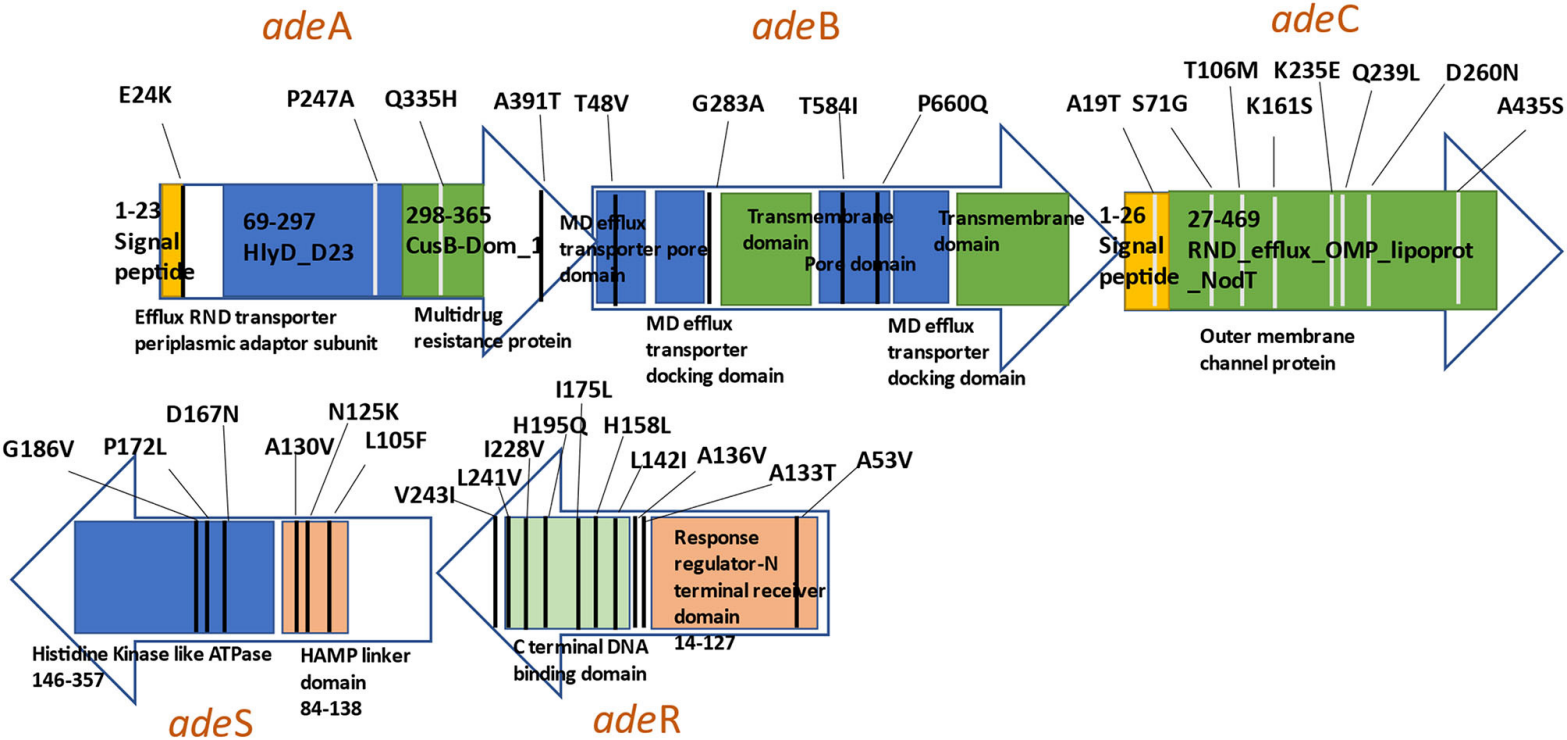
Diagram illustrating significant genetic variants observed in the *AdeABC* efflux pump.

All 117 variants were analyzed for their significant correlation and predictive capacity toward the carbapenem-susceptibility phenotype, and the results of the group of variants showing a significant outcome are summarized in Tables 1, 2. A total of 67 and 76 variants exhibited significant differential distributions in relation to imipenem- and meropenem-susceptibility phenotypes, respectively. Among these, 27 variants were significantly encountered in both the imipenem and meropenem non-susceptible groups, while 40 variants and 49 variants were significantly more distributed in relation to imipenem- and meropenem-susceptible sequences, respectively. The position of variants in relation to each gene structure is diagrammatically illustrated in Figure 3.

**Table 1:** Efflux-pump variants in the carbapenem non-susceptible group.

**Variant**	***p*** **-value for the chi-squared test of significance**		**Specificity**		**Positive predictive value (PPV)**		**PROVEAN score**	**Prediction (−2.5 cutoff)**
	**Meropenem**	**Imipenem**	**Meropenem**	**Imipenem**	**Meropenem**	**Imipenem**		
*AdeA* Q335H	0.005	0.007	92.9	91.4	77.9	55	−1.703	Neutral
*AdeB* G283A	0.002	0	100	99.8	100	93.3	−5.593	Deleterious
*AdeB* A551T	0.017		74.9		69.9		−0.233	Neutral
*AdeB* T606A	0.019	0.022	91.5	90.7	75	53.2	−0.047	Neutral
*AdeB* L730F	0.013	——-	75	——	70.1	——	−2.401	Neutral
*AdeC* A19T	——–	0.019	——-	91.8	——–	54	−0.548	Neutral
*AdeC* D260N	0.001	0	92	90.9	79.5	58.2	0.037	Neutral
*AdeR* A136V	0.021	——-	74.5	——	69.5	——	−1.168	Neutral
*AdeR* I175L	0	0	94.3	94.3	82.9	71.4	1.206	Neutral
*AdeR* H158L	0.001	0.037	96.7	94.8	85.1	55.4	−2.425	Neutral
*AdeS* L105F	0	——	100	——	100	—–	−3.954	Deleterious
*AdeS* N125K	——-	0.009	——-	99.6	——-	81.8	−2.997	Deleterious
*AdeS* A130V	——	0.002	——–	99.6	——–	84.6	−3.963	Deleterious
*AdeS* G186V	0.002	0.039	75	66.7	71.7	47.3	−8.183	Deleterious
*AdeS* H189Y	0	0.019	97.6	95.4	88.6	57.4	−2.046	Neutral
*AdeS* V235I	0.011	—–	94.8	——	78.8	—–	−0.566	Neutral
*AdeS* I331V	——-	0	——-	86.3	——–	56.7	−0.723	Neutral
*AdeS* G336S	0.031	0.013	94.3	94.3	76.5	56.8	5.479	Neutral
*AdeS* V348I	0.002	——	74.5	—–	71.3	—–	0.156	Neutral
*AdeS* I360M	0.008	0.005	91.5	90.9	76.3	55.3	−0.093	Neutral
*AdeI* A388T	0	0.009	98.6	95.9	92.7	59.6	−0.548	Neutral
*AdeN* P16L	——-	0.004	——–	99.5	——-	80	−4.442	Deleterious
*AdeN* P92T	0	0	100	99.6	100	91.3	−0.423	Neutral
*AdeN* K216N	0	0	99.1	98.9	96.6	92.9	−1.339	Neutral
*AdeG* A350T	0.003	0.004	92.5	91.3	78.1	55.9	−1.770	Neutral
*AdeF* S172F	0.025	0.001	99.5	99.6	92.3	85.7	−2.494	Neutral
*AdeL* D227A	0	0.02	99.1	99.1	93.3	70.6	−4.373	Deleterious

**Table 2:** Efflux-pump variants in the carbapenem-susceptible group.

**Variant**	***p*** **-value for the chi-squared test of significance**		**Specificity**		**Positive predictive value (PPV)**		**PROVEAN score**	**Prediction (−2.5 cutoff)**
	**Meropenem**	**Imipenem**	**Meropenem**	**Imipenem**	**Meropenem**	**Imipenem**		
*AdeA* E24K	0.009	——-	100	——	100	——	−1.415	Neutral
*AdeA* P247A	——-	0.043	——	99.5	—–	84.6	−2.399	Neutral
*AdeA* A391T	0.004	0	90.7	89.8	52.9	75.8	−0.219	Neutral
*AdeB* T48V	0.012	0.006	99.4	99.8	77.8	92.9	−3.302	Deleterious
*AdeB* A54P	0.008	0.034	99.7	99.8	85.7	90	4.125	Neutral
*AdeB* T87M	0.02	——-	99.7	——	83.3	——	−1.196	Neutral
*AdeB* E89Q	0.027	0.015	99.4	99.8	75	91.7	0.055	Neutral
*AdeB* F178Y	——-	0.021	——-	100	——	100	−2.485	Neutral
*AdeB* N205A	0.001	——–	100	——	100	—–	1.243	Neutral
*AdeB* E322Q	0.001	0.015	99.7	99.8	88.9	91.7	−0.743	Neutral
*AdeB* S426P	0.001	0.015	99.7	99.8	88.9	91.7	−2.154	Neutral
*AdeB* G502E	0.003	0.022	99.7	99.8	87.5	90.9	−1.558	Neutral
*AdeB* G547A	0.003	0.022	99.7	99.8	87.5	90.9	−0.142	Neutral
*AdeB* Y555F	0.003	0.015	99.7	99.8	87.5	91.7	−0.817	Neutral
*AdeB* T584I	——-	0	—–	90.5	——	73.3	−5.974	Deleterious
*AdeB* D642A	0	0.003	93.2	93.4	68.4	71.1	2.928	Neutral
*AdeB* S645T	0	0	91	91.3	68	73	−0.044	Neutral
*AdeB* P660Q	0.012	——	99.4	——	77.8	—–	−7.601	Deleterious
*AdeC* S71G	0	0	85.3	84.2	52.7	69.8	−0.684	Neutral
*AdeC* T106M	0	0.005	98.3	97.6	81.8	77.3	−2.257	Neutral
*AdeC* K161S	0	——–	98.3	——	72.7	—–	−1.988	Neutral
*AdeC* K235E	——-	0	——	90.5	——	73.3	−0.244	Neutral
*AdeC* Q239L	——-	0.001	——	88.2	——	69.1	−6.106	Deleterious
*AdeC* A435S	0	0.001	100	100	100	100	−1.982	Neutral
*AdeR* A53V	0.025	———	100	—–	100	——	−0.362	Neutral
*AdeR* A133T	0	0.002	100	100	100	100	−0.717	Neutral
*AdeR* L142I	0	0	87.9	87.9	53.8	74	0.780	Neutral
*AdeR* H195Q	0.007	0.033	99.2	99.3	75	82.4	0.52	Neutral
*AdeR* I228V	0.001	0.037	98.6	98.8	73.7	78.3	0.660	Neutral
*AdeR* L241V	0	0.019	99.4	99.5	84.6	86.7	0.385	Neutral
*AdeR* V243I	0	0	89.3	89.1	58.7	76.5	−0.019	Neutral
*AdeS* D167N	0.041	0	92.1	91.3	50	75.2	−3.208	Deleterious
*AdeS* P172L	0.025	——-	100	——	100	—–	−8.811	Deleterious
*AdeS* V279A	0	0.002	97.7	98.1	70.4	80.5	1.301	Neutral
*AdeS* V332M	0	0.014	98.3	98.3	75	78.1	0.224	Neutral
*AdeJ* P506L	0.048	——	99.7	——	80	—–	−1.237	Neutral
*AdeJ* S517G	0.001	——	100	—–	100	—–	1.188	Neutral
*AdeJ* V573M	—–	0.034	——	97.6	—–	73.7	−1.403	Neutral
*AdeK* A437S	0.009	——-	100	——	100	—-	0.681	Neutral
*AdeN* Q94R	0.001	0.002	97.5	98.3	67.9	81.6	−0.113	Neutral
*AdeN* H170Y	0.048	——-	99.7	——	80	—-	0.282	Neutral
*AdeN* G177E	0.012	——-	99.4	——	77.8	—–	−5.102	Deleterious
*AdeN* N195H	——-	0.022	——	99.8	——	90.9	−1.853	Neutral
*AdeN* N195M	——-	0.022	——	99.8	——	90.9	−2.866	Deleterious
*AdeN* T197M	0	0.001	99.4	99.8	66.7	94.7	−0.846	Neutral
*AdeG* D501N	——-	0	——	90.5	——	73.3	−1.336	Neutral
*AdeF* N269D	0.032	0.043	99.2	99.5	70	84.6	4.148	Neutral
*AdeH* S5V	0	0	100	100	100	100	0.07	Neutral
*AdeH* R100G	0.001	——	98	——	69.6	——	−6.815	Deleterious
*AdeH* Q141H	0.018	——	98.6	——	66.7	——	−2.085	Neutral
*AdeH* A276P	0.018	——-	98.6	——	66.7	——-	0.389	Neutral
*AdeH* A289G	0.003	——–	96.9	——	63.3	——–	−2.704	Deleterious
*AdeH* N388H	0.005	——-	96.9	——	62.1	——-	−1.969	Neutral
*AdeL* H5N	0.027	——–	99.4	—–	75	——–	−0.471	Neutral
*AdeL* Q262R	——-	0.002	——	80.4	—–	65.6	−2.442	Neutral
*AdeL* Y339R	0	0	98.9	99.5	80	93.3	0.213	Neutral
*AdeL* T340V	0	0	98.9	99.5	80	93.3	0.266	Neutral

Those showing a significant correlation to any of the susceptibility phenotypes were further tested for their predicted effect on gene function. The *p*-values indicating the significance of both imipenem and meropenem association, as well as the predicted functional effects of these variants, are shown in Tables 1, 2. These variants were also further mapped to the predicted secondary structure of each corresponding protein. Only specificity and PPV for each variant were considered. The sensitivity of the variants was not considered in this study. The variants identified did not occur at a high frequency in the population; therefore, they were not considered suitable candidates for use as screening markers, and as a result, their sensitivity was not assessed.

### 3.3. Distribution of membrane protein genes, PBPs, and related variants in *A. baumannii*

Regarding the distribution of membrane protein genes, Omp33-36 was completely absent in two sequences, while 292 other sequences showed hits below the threshold used for gene identification. Additionally, for OmpA, 52 sequences showed hits below the threshold used for gene identification. Genes identified below the threshold could be disrupted by insertion sequences and could be non-functional. OmpW was completely absent in nine sequences. OprB was absent in 1 sequence, while OprD was completely absent in 4 sequences, with 79 other sequences showing hits below the threshold used for identification. Three protein variants were identified for Omp33-36. These were further analyzed for their relation to carbapenem susceptibility. For OmpA, most studied sequences showed two variants. OmpW sequence appeared to be highly conserved, with V1 being the most frequently occurring variant and another two variants that were observed at a lower frequency. OmpW T18A was observed and further studied. Three variants were observed in OprD, with V2 and V3 being observed in fewer isolates. Two variants were observed in OprB, with V2 being observed in fewer isolates. Substitutions such as OprB F57L, OprB T304A, OprB S360N, and OprB G403S were observed and further analyzed.

CarO sequence was identified in 71 sequences, with hits below the threshold used for gene identification. CarO1 showed a significant correlation to imipenem resistance, while CarO3 variants showed a correlation to meropenem resistance, although it was non-significant. Omp33_V2 showed a significant correlation to the imipenem-susceptibility phenotype, while Omp33_V3 showed a significant correlation to the meropenem-susceptibility phenotype. OmpA_V1 showed a significant correlation to imipenem and meropenem resistance, while OmpA_V2 showed a significant correlation to imipenem susceptibility. OprD_V2 showed a significant correlation to meropenem susceptibility, while OprD-V3 showed a significant correlation to imipenem susceptibility. OprB G403S showed a significant correlation to both imipenem and meropenem resistance. OprB N360S showed a significant correlation to imipenem susceptibility, while OprB F57L was significantly correlated to meropenem susceptibility.

FASTA sequences of the studied variants are provided in Supplementary Table S3. The significance of the variants in relation to each of the imipenem and meropenem-susceptibility phenotypes along with the predictive values for the observed variants in membrane protein genes are shown in Table 3.

**Table 3:** Correlation of membrane-protein variants with carbapenem susceptibility phenotypes.

**Variant**	**Correlation with (*p*-value)**	**Positive predictive value (PPV) %**	**Specificity %**	**I-Mutant prediction**
OprB F57L	Meropenem susceptibility (0.027)	75	99.4	Large Decrease in Stability
OprB G403S	Imipenem resistance (0.001)	91.7	99.8	Large Decrease in Stability
OprB G403S	Meropenem resistance (0.01)	100	100	Large Decrease in Stability
OprD_V2	Meropenem susceptibility (0.003)	66.7	97.7	Neutral
OprD_V3	Imipenem susceptibility (0.019)	86.7	99.5	Neutral
OmpA_V1	Imipenem resistance (0.007)	46.7	46.7	Neutral
OmpA_V1	Meropenem resistance (0.003)	68.2	54.2	Neutral
OmpA_V2	Imipenem susceptibility (0.005)	76	97.2	Neutral
Omp33_V2	Imipenem susceptibility (0.04)	62	73.3	Neutral
Omp33_V3	Meropenem susceptibility (0.001)	63.6	96.6	Neutral
CarO1	Imipenem resistance (0.000)	55.4	78.1	Neutral

PBPs previously reported in the literature (listed in Supplementary Table S2), in addition to other observed variants, were explored for their correlation to imipenem- and meropenem-susceptibility phenotypes. Variants showing a significant correlation to imipenem- and meropenem-susceptibility/resistance, together with their predictive values and functional effect on protein stability, are shown in Tables 4, 5, respectively.

**Table 4:** Correlation of different PBPs variants with imipenem susceptibility phenotypes.

**Variant**	**Correlation with (*p*-value)**	**Positive predictive value (PPV) %**	**Specificity %**	**I-Mutant prediction**
PBP1a_T38A	Resistance (0.000)	59.8	91.3	Large decrease in stability
PBP1a_S382N	Susceptibility (0.000)	80.2	95.5	Neutral
PBP1a_T636A	Susceptibility (0.000)	82.6	94.6	Large decrease in stability
PBP1a_T776A	Resistance (0.002)	59.5	94.3	Large decrease in stability
PBP1b_N513H	Resistance (0.005)	55.5	91.3	Large decrease in stability
PBP1b_P764S	Susceptibility (0.000)	73.3	89.8	Large decrease in stability
PBP3_G523V	Susceptibility (0.000)	72.3	90.3	Neutral
PBP3_H370Y	Susceptibility (0.000)	96.4	99.8	Neutral
PBP2_K152Q	Resistance (0.000)	72.9	94.8	Large decrease in stability
PBP2_P665A	Resistance (0.003)	53.8	87.2	Large decrease in stability
PBP2_V509I	Susceptibility (0.000)	73.7	90.5	Large decrease in stability
PBP78_S32T	Resistance (0.001)	64.4	96.3	Large decrease in stability
*MtgA*_T49P	Susceptibility (0.000)	59.7	21	Large decrease in stability
*MtgA*_V54I	Susceptibility (0.000)	61.3	29.3	Large decrease in stability
*MtgA*_N122K	Resistance (0.004)	80	99.5	Large decrease in stability
*MtgA*_E225Q	Susceptibility (0.034)	90	99.8	Large decrease in stability

**Table 5:** Correlation of different PBPs variants with meropenem susceptibility phenotypes.

**Variant**	**Correlation with (*p*-value)**	**Positive predictive value (PPV) %**	**Specificity %**	**I-Mutant prediction**
PBP1a_T38A	Resistance (0.001)	79.5	92	Large decrease in stability
PBP1a_T636A	Susceptibility (0.000)	66	95.5	Large decrease in stability
PBP1b_S112P	Resistance (0.015)	59.2	25	Neutral
PBP1b_N513H	Resistance (0.003)	78.1	92.5	Large decrease in stability
PBP3_H370Y	Susceptibility (0.027)	75	99.4	Neutral
PBP2_K152Q	Resistance (0.000)	85.1	95.3	Large decrease in stability
PBP6_N329S	Resistance (0.013)	70.1	75	Large decrease in stability
PBP6b_S28P	Resistance (0.015)	59.2	25	Neutral
PBP78_T71A	Resistance (0.038)	67.5	63.7	Large decrease in stability
*MtgA*_L18F	Susceptibility (0.003)	87.5	99.7	Neutral
*MtgA*_V54I	Susceptibility (0.000)	42.3	30.5	Large decrease in stability
*MtgA*_F4V	Susceptibility (0.001)	69.6	98	Large decrease in stability
*MtgA*_E225Q	Susceptibility (0.009)	100	100	Large decrease in stability

**Figure 4:**
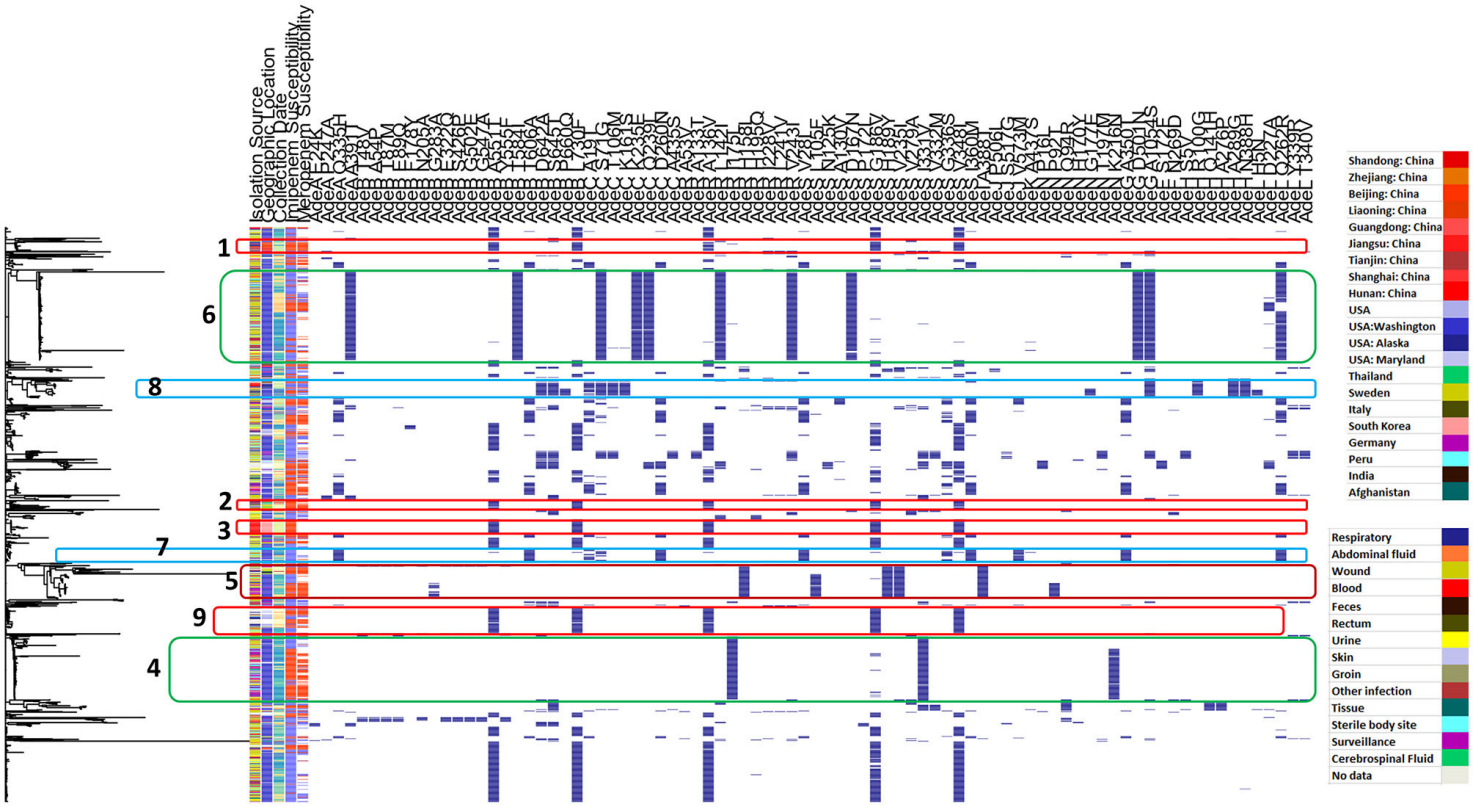
Distribution of different efflux pump gene variants and carbapenem-resistance phenotypes in relation to the genetic-relatedness phylogram. Dark blue color indicates the presence of an efflux-pump gene variant. Blue color indicates carbapenem susceptibility, while red color indicates non-susceptibility to carbapenems.

**Figure 5:**
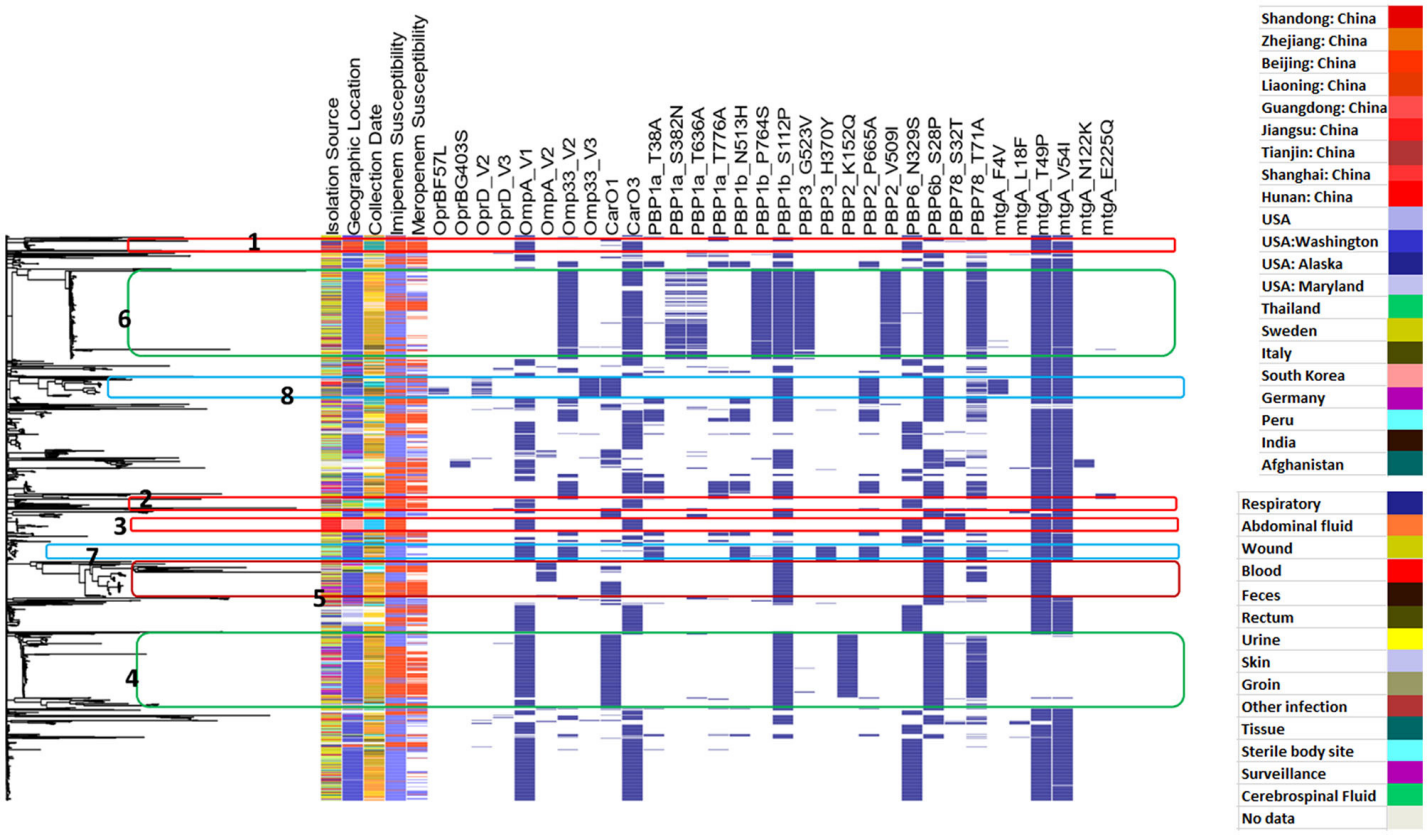
Distribution of different membrane-protein gene variants and carbapenem-resistance phenotypes in relation to the genetic-relatedness phylogram. Dark blue color indicates the presence of a membrane protein variant. Blue color indicates carbapenem susceptibility, while red color indicates non-susceptibility to carbapenems.

Figure 4 shows the distribution of different efflux-pump gene variants and carbapenem-resistance phenotypes in relation to the genetic-relatedness phylogram, while Figure 5 shows the distribution of different membrane protein gene variants and carbapenem-resistance phenotypes in relation to the genetic-relatedness phylogram.

## 4. Discussion

*AdeABC* is a chromosomal RND-type efflux system. It was the first efflux pump described in *A. baumannii* in 2001, followed by *AdeIJK* in 2008 and *AdeFGH* in 2010. *AdeABC* is the most frequently studied type, and the literature shows evidence that the detection rate of *AdeB* is the highest in clinical isolates compared to *AdeFGH* and *AdeIJK*, although variable among other strains (Xu et al., 2019). Based on MIC changes in different pump mutants, it has been assumed that the three carbapenems could act as substrates for *AdeABC*, while only meropenem could be a substrate for the *AdeIJK* pump (Yoon et al., 2015).

Researchers have also shown that overexpression of the *AdeABC* system could significantly contribute to resistance against meropenem in *A. baumannii* clinical isolates (Cardoso et al., 2016). The high incidence of *AdeABC* efflux-pump hyper-expression in MDR *A. baumannii* has been outlined in several reports and linked to several mutations in the corresponding two-component regulatory system (Yoon et al., 2013). These mutations have been revised and explored in the current study set. Some studies have shown the universal presence of RND-type efflux pumps and their variable expression level, highlighting the complexity of their regulatory expression and the role of expressed pumps in MDR (Pagdepanichkit et al., 2016). *AdeABC* has been described to be common in imipenem-resistant *A. baumannii*, leading to higher levels of resistance when combined with other resistance-conferring elements (Xiao et al., 2016). More recently, the contributions of *AdeABC* and *AdeIJK* to drug resistance and growth physiology have been analyzed, showing different specificities and non-overlapping changes in both pump systems (Leus et al., 2020).

Different studies have investigated the distribution of different efflux-pump-related genes, their expression, and their correlation to the MDR phenotype (Lin et al., 2017; Chen et al., 2018; Basatian-Tashkan et al., 2020). Although it has been proposed that high resistance levels do not occur because of efflux-pump genes, their variable expression levels cannot be ignored in relation to MDR (Basatian-Tashkan et al., 2020). Furthermore, the presence of the *AdeABC* genes in sensitive strains is assumed to be low, suggesting the use of *AdeABC* as a sign of resistance in *A. baumannii* (Lin et al., 2009; Xu et al., 2019). No direct evidence confirming the link between the presence/absence of porin proteins and efflux systems and carbapenem susceptibility in *A. baumannii* has been found, with a study concluding that intrinsic resistance mechanisms, including both membrane proteins and efflux pumps, do not play a major role in carbapenem resistance; however, the study may have underestimated this role as it included only 14 isolates from one geographic locality (Wong et al., 2019). This needs to be carefully studied and analyzed in an extensive collection of sequences, especially focusing on different variants and their potential role in modifying the expression or functionality of these genes, which was applied in the current study.

However, the *AdeIJK* efflux pump has been detected in both sensitive and resistant isolates and is considered a factor of intrinsic resistance with the average expression level of *AdeJ* being relatively low (Coyne et al., 2011) or entirely non-overexpressed in some studies (Yoon et al., 2013). Its regulatory gene *AdeN* has been shown to have a high level of amino acid conservation (Touchon et al., 2014). Mutated or truncated inactive *AdeN* protein has been shown to result in *AdeIJK* pump overexpression (Rosenfeld et al., 2012), which underlies meropenem and doripenem resistance (Fernando et al., 2014). The overexpression of the *AdeIJK* efflux pump because of mutations in the negative regulator *AdeN* has been related to the increase in meropenem MIC (Fernando et al., 2014). No studies have directly compared the effect of *AdeABC* and *AdeIJK* efflux pumps, and further studies were recommended to understand the importance of the *AdeIJK* effect on *A. baumannii* (Xu et al., 2019).

The *AdeABC* system is under the regulatory control of the *AdeRS* two-component regulatory system, which contains a sensor kinase coding gene *AdeS* and a response regulator coding gene *AdeR* (Marchand et al., 2004). *AdeRS* operon is known to express high genetic variability with different alleles and variants being reported, especially the *AdeS* gene (Sun et al., 2014; Hammerstrom et al., 2015; Montaña et al., 2015). A list of previously reported substitutions in these two genes is shown in Supplementary Table S2.

*AdeFGH* is another RND efflux system conferring MDR when overexpressed, and its structural genes are under the regulation of the upstream-located *AdeL*. It is not constitutively expressed in wild-type strains, so it is not believed to contribute to intrinsic resistance (Coyne et al., 2011).

### 4.1. Distribution of efflux-pump genes

Previous studies have shown *AdeAB* and *AdeRS* to be highly distributed among MDR AB clinical isolates, while *AdeC* was less frequently distributed (Wieczorek et al., 2013). In their study, Wieczorek et al. (2013) showed that *AdeB* was found throughout the studied strains. In the same study, three strains had no *AdeR* gene, and a single strain had no *AdeA* gene or *AdeS* gene. *AdeC* was negative in 16 strains among 100 studied randomly selected isolates, making it the least abundant. Other studies have reported the distribution of *AdeB* at 70% (Chu et al., 2006), 75% (Lin et al., 2009), and 87% (Nemec et al., 2007). The latter study demonstrated the absence of *AdeA, AdeB, AdeR*, and *AdeS* in 11 out of 116 studied isolates, while *AdeC* was detected at a much lower frequency (only in 49 out of 116) (Nemec et al., 2007). These results align with those obtained from the analysis performed in this study; however, our results may be more representative as they include the analysis of a diverse group of sequences that are timely and geographically separated.

A more recent study investigated the distribution of *AdeABC* and *AdeRS* genes among 75 *A. baumannii* environmental isolates isolated from contaminated soil. The study determined the distribution of *AdeA* at 66.6% (50/75), *AdeB* at 60% (45/75), *AdeC* at 30.6% (23/75), *AdeR* and *AdeS* at 54.6% (41/75), and 48% (36/75) of isolates, respectively (Al-Tofaily et al., 2020).

Other studies have also shown a high distribution of *AdeABC-AdeRS* efflux-system genes in CRAB strains, which was reported at 90%, and a significantly increased distribution of the regulator genes *AdeR* and *AdeS* in CRAB strains as compared to CSAB strains. CRAB strains also showed a significantly increased expression of *AdeB* (12.3-fold) but a decreased expression of *AdeR* (3.3-fold) (Chen et al., 2018). Another study concluded the universal presence of some efflux-pump genes and correlated *AdeB* and *AdeJ* upregulation to MDR; however, the study only included 21 isolates (Lin et al., 2017). Similarly, other investigators have reported the high prevalence of *AdeA, AdeB*, and *AdeC* genes among clinical isolates of *A. baumannii* showing imipenem and meropenem resistance. The efflux-pump genes occurred at the frequencies of 96.2, 96.2, and 91.1%, respectively, and were significantly related to imipenem resistance (Ranjbar et al., 2020).

Both *AdeA and AdeS* were also detected at a high frequency among *A.baumannii* clinical isolates, where 80% (48/60) of *A. baumanii* isolates had the *AdeA* gene and 81.66% (49/60) of isolates had the *AdeS* gene (Basatian-Tashkan et al., 2020). Other researchers have also identified both genes at similar frequencies of 79.6 and 80.6% for the *AdeA* and *AdeS* genes, respectively (Jia et al., 2015). Nemec et al. (2007) identified both *AdeA* and *AdeS* at a similar distribution of 81.9%. Contrarily, *AdeA* was identified at a frequency of only 62.7% in other studies (Kor et al., 2014). In another study, the *AdeR* and *AdeS* genes were detected in 36/60 (60%) and 59/60 (98.3%), respectively (Ostad Asadolah-Malayeri et al., 2016). In yet another study, *AdeRS* was positive in 31/37 (84%) of *A. baumannii* isolates studied (Montaña et al., 2015). Another study reported the presence of different pump genes at the frequencies of 60 (92%) for *AdeA*, 40 (61.5%) for *AdeB*, and 55 (84.6%) for *AdeC*, while *AdeR* was detected upstream of the *AdeABC* operon in 38 (58.4%) of the studied isolates (Modarresi et al., 2015). Another study identified the *AdeR, AdeS*, and *AdeB* genes at the percentages of 96.8 (61/63), 63.4 (41/63), and 79.3 (50/63) of isolates, respectively (Hassan et al., 2018).

In addition to *AdeABC*, two other RND pumps, *AdeIJK* and *AdeFGH*, also contribute to resistance in the *A. baumannii* clinical isolates. Coyne et al. (2010) showed that *AdeABC* was detected in 80% of clinically isolated resistant strains, while *AdeFGH* operon was identified in 36 out of 40 *A. baumannii* clinical isolates. Researchers have also identified *AdeL* mutations in three *AdeFGH*-overexpressing mutants in the same study set.

In another study, the genes of the *AdeABC, AdeFGH*, and *AdeIJK* efflux pumps existed in all AB-XDR clinical isolates. The transcriptional level of *AdeA* was significantly higher than the *AdeF* and *AdeI* genes. Seventy out of 81 (70/81) isolates (86.4%) overexpressed *AdeABC*, and only 17 out of 81 isolates (21.0%) overexpressed *AdeIJK*, while no isolate overexpressed the *AdeFGH* pump system (Sun et al., 2014).

Positive *AdeB* and *AdeJ* were detected in 47 (94%) and 45 (90%) of the 50 studied imipenem-resistant isolates, respectively. Forty-three out of 47 (43/47) *AdeB*-positive imipenem-resistant *A. baumannii* (IRAB) carried the *AdeRS* regulatory system, which was absent in four isolates. Six (12%) isolates of IRAB carried *AdeB* and *AdeE* simultaneously. In addition, *AdeB* and *AdeJ* showed approximately 4- and 3-fold increases in expression levels in imipenem-resistant isolates as compared to imipenem-susceptible isolates (Hou et al., 2012). Both *AdeA* and *AdeI* showed overexpression in MDR strains (Leus et al., 2020).

The reason for some observed differences may be attributed to variations in antibiotic usage patterns in the studied localities, the type of clinical samples isolated, the number of samples studied, the sampling method, environmental factors, or differences in geographical distribution of these genes. However, we believe that the frequencies identified in the current analysis better reflect the general epidemiology of these pump genes due to the inclusive and representative nature of this study.

### 4.2. Role of efflux-pump gene variants

*AdeABC* was the first RND-type efflux pump discovered in *A. baumannii* and has been extensively studied. *AdeA* is the membrane fusion protein part of the efflux pump acting as a periplasmic adapter with transmembrane transporter activity. It facilitates interaction between transporters and outer membrane channels and links the reactions between the inner and outer membranes together (Zgurskaya et al., 2015). As a membrane fusion protein, *AdeA* is a linear protein formed of four characteristic domains (Supplementary Figure S1A) that extend from the inner membrane into the periplasm to meet the outer membrane channel (Mikolosko et al., 2006).

In the current analysis, *AdeA* Q335H was observed in the carbapenem non-susceptible group. The variant was located at the upper end of the membrane-proximal domain at the start of a beta sheet at the upper pocket near the short flexible linker between the membrane-proximal domain and the beta-barrel domain (Supplementary Figure S1B). These linker regions are known to be highly flexible and are capable of making conformational changes due to high structural variation that results in the transmission of energy from the inner membrane to the outer membrane, which is needed for drug efflux (Zgurskaya et al., 2015).

GLN at 335 links through a 3.1 hydrogen bond to ILE 342. When GLN was mutated to HIS, no change in the hydrogen bonding was observed. It appears that the nature of the change of the polar glutamine to histidine with the imidazole side chain may be needed to effectively participate in catalysis. Positively charged residues located at the interface between the β-barrel and the MP domains are important for both binding to the core LPS and the functionality of the pump (Shuo et al., 2013). Researchers have also observed that the protonation/deprotonation of HIS underlies the PH-regulated conformational dynamics of AcrA (Wang et al., 2012), a similar MFP efflux pump. Twisting of the MP domain relative to the beta-barrel domain has been previously observed among the conformational changes needed for the efflux process. This twisting may be necessary for the steps of access and binding of the drug to the efflux transporter (Symmons et al., 2009). Based on this, the change of GLN to HIS at position 335 may contribute to a more stable binding, potentially leading to more effective efflux and contributing to resistance.

Three other variants were identified in the carbapenem-susceptible group: *AdeA* E24K, *AdeA* A391T, and *AdeA* P247A. *AdeA* P247A was located in the middle of a coil connecting the two groups of beta strands that form the beta-barrel domain, as shown by the predicted structure using AlphaFold (Supplementary Figure S1C). Although the mechanism is not clear, the change of PRO to ALA appears to destabilize drug-binding, thereby compromising the efflux process. Although *AdeA* E24K was located in an area of very low model confidence, as predicted by the AlphaFold model, position 24 was located just next to the predicted signal peptide found at the start of the efflux RND transporter periplasmic adaptor subunit. Similarly, *AdeA* A391T was located in a predicted coil at the end of the multidrug resistance protein and also in an area of very low model prediction confidence. Due to their location in an area of low model prediction confidence, the functional effects of these variants could not be hypothesized.

*AdeF* and *AdeI* components of the efflux pumps *AdeIJK* and *AdeFGH*, respectively, perform a similar periplasmic adapter function as that of *AdeA*. In this analysis, *AdeI* A388T and *AdeF* S172F variants were detected in the carbapenem non-susceptible group. *AdeF* S172F was located at the lower side of the α-helical hairpin that spans the periplasm (Supplementary Figure S2). The α-helical hairpins are known to oligomerize and assemble a funnel-like structure that interacts with the outer membrane channels to help extrude the drug. It is postulated that the interaction between the MFP transporter and the outer membrane channels is dynamic and results from conformational changes that transmit energy, resulting in open-channel conformations that extrude the drug (Tikhonova et al., 2007). SER at position 172 appears to be tightly bonded to the surrounding residues, forming 5 hydrogen bonds within its containing alpha helix. These include two hydrogen bonds (3A and 3.3A) with ASN 176, a 3.2 A bond with LEU 175, a 3.9 A bond with ALA 169, and a 2.9 A bond with ALA 168. The change of SER to the bulky hydrophobic non-polar PHE may result in chain instability; this may consequently lead to conformational changes because of the hydrophobic interaction with the nearby non-polar amino acids, which is known to stabilize folded protein conformation. *AdeI* A388T was located in a predicted coil at the end of the proteins in an area of very low model prediction confidence, so its function could not be predicted.

Both the multidrug transporter *AdeB* and outer membrane channel *AdeC* are considered structural proteins of the pump that promote drug discharge, where *AdeB* captures the substrate in the inner membrane of the phospholipid bilayer or cytoplasm, while *AdeC* transports the substrate outside the cell (Xu et al., 2019). The *AdeB* shows the highest detection rate in clinical isolates and is considered to be most vitally related to resistance in *A. baumannii* (Yoon et al., 2016). The three variants *AdeB* T48V, *AdeB* T584I, and *AdeB* P660Q were significantly detected in the carbapenem-susceptible group and were predicted to be deleterious mutations. The mutations were mapped on the PDB available 3D structure found in the RCSB Protein Data Bank with accession number 6OWS.

P660 is one of the important residues for substrate recognition and binding at the periplasmic cleft multidrug-binding sites (Su et al., 2019). *AdeB* P660Q was located at the apex periplasmic binding cleft between the subdomains PC1 and PC2 at the start of the F loop that connects the cleft entrance to the proximal drug-binding pocket (Su et al., 2019) (Supplementary Figure S3A). On the contrary, both *AdeB* T48V and *AdeB* T584I were located at the distal part of the binding cleft (Supplementary Figure S3A). *AdeB* T48V was located at the last position of the first beta strand located approximately in the middle of the multidrug efflux transporter pore domain. Similarly, *AdeB* T584I was located in the second predicted multidrug efflux transporter pore domain. It was located at the beginning of an alpha helix just opposite T48V. It appears that these mutations in the periplasmic cleft affect binding to the carbapenem molecule and that destabilization of its structure affects this binding, consequently affecting drug transport and discharge outside the cell. This would consequently make the cell carbapenem susceptible by preventing carbapenem efflux. These positions may act as probable targets for new efflux pump inhibitors.

*AdeB* G283A was located at the junction of two small beta sheets in close proximity to the multidrug efflux transporter pore domain but on the outer side. The residue was located near the distal drug-binding site (Supplementary Figure S3B). It may also have a role in binding to the carbapenem molecule. It was predicted to be deleterious and was significantly detected in the carbapenem-resistant group.

Similar to *AdeB*, both *AdeG* and *AdeJ* recognize and transport substrates through a drug-proton antiporter mechanism (Ruggerone et al., 2013). *AdeG* A350T was identified in the carbapenem non-susceptible group, while *AdeG* D501N, *AdeJ* P506L, *AdeJ* S517G, and *AdeJ* V573M were identified in the carbapenem-susceptible group.

*AdeJ* P506L, *AdeJ* S517G, and *AdeJ* V573M were mapped on the *AdeJ* efflux-pump structure available at PDB (7M4Q). *AdeJ* V573M is located near the proximal drug-binding site (Zhang et al., 2021) (Supplementary Figure S4). It has been proposed that non-polar residues at such drug-binding sites are generally involved in the recognition of different antimicrobials through hydrophobic interactions (Zhang et al., 2021). Although both are non-polar hydrophobic amino acids, the change of valine to methionine may affect drug recognition, binding, and, consequently, its extrusion because methionine residue contains sulfur atoms, which are more susceptible to oxidation.

*AdeG* A350T and *AdeG* D501N were mapped to the AlphaFold-predicted 3D structure (Supplementary Figure S5). *AdeG* A350T was linked to carbapenem resistance and was located in the middle of an alpha helix at the drug-access site near the inner membrane. *AdeG* D501N was also located at the lower end of the molecule in the middle of a predicted loop just below the inner membrane. The functional effect of these mutations needs to be further studied.

*AdeC* is an outer membrane channel protein that was reported to occur at the lowest frequency, and it is considered not necessary for *AdeABC*-mediated drug efflux. However, its presence usually correlates with multidrug resistance or pan-resistance (Xu et al., 2019). *AdeC* A19T was located at the area of the alpha helix identified at very low confidence prediction by AlphaFold and was related to carbapenem resistance. *AdeC* S71G was located at the center of the third alpha helix, forming a pocket just parallel to the four large alpha helices, while the *AdeC* Q239L deleterious mutation was located in the opposite alpha helix at the boundaries of the same pocket (Supplementary Figure S6). Both variants were observed in the carbapenem-susceptible group. *AdeC* T106M was located at the first position of the first beta sheet at the junction between the beta sheets and alpha helices and was also observed in the carbapenem-susceptible group. The mentioned variants appear to affect the function of *AdeC* in transporting carbapenem molecules outside the cell; however, the dynamics related to this need to be further studied.

Similar to *AdeC*, both *AdeH* and *AdeK* provide channels for substrates to cross the outer membrane. Two variants, *AdeH* R100G and *AdeH* A289G, were significantly related to meropenem susceptibility, showing high specificity, and they were predicted to be deleterious (Supplementary Figure S7).

The function of *AdeS* is to receive the environmental signals causing autophosphorylation and then to transfer the phosphoric acid to the output responder *AdeR*, which acts as a transcriptional activator (Marchand et al., 2004). The *AdeS* structure was analyzed in this study, and two domains were predicted using different tools. These domains include the HAMP linker domain and the histidine kinase domain. It has been suggested that the HAMP domain with the alpha-helical regions common to many chemoreceptors plays a role in regulating the phosphorylation of homo-dimeric receptors by transmitting the conformational changes in periplasmic ligand-binding domains to a cytoplasmic signaling kinase (Aravind and Ponting, 1999). It has also been proposed that the two HAMP alpha helices take two different conformational states depending on the ligand-bound state of the chemoreceptor and that a transmembrane signaling helix can switch between these conformational states. Furthermore, it has been proposed that the bihelical HAMP domain of monomeric histidine kinases binds intramolecularly to the bihelical dimerization domain and that ligand-mediated change in HAMP domain conformation leads to the subsequent formation of the four-helix bundle within histidine kinase dimers (Aravind and Ponting, 1999).

Three deleterious mutations were observed in the HAMP linker domain, *AdeS* L105F, *AdeS* N125K, and *AdeS* A130V. Mapping these variants on the AlphaFold-predicted structure showed that L105F was located at the turn between two coils, while A130V was located just opposite to it near the start of the following alpha helix. Similarly, N125K was located in the same area (Supplementary Figure S8A). It appears that L105F may have a role in making bihelical dimerization conformational changes, helping to transmit phosphorylation reactions. Supporting this is the nature of the substitution of leucine into the bulky hydrophobic phenylalanine residue with an aromatic ring that is known to stabilize the folded conformation of proteins and that frequently forms the cores of protein folds.

Three other deleterious variants were observed in the histidine kinase domain, *AdeS* D167N, *AdeS* P172L, and *AdeS* G186V. Previous studies have shown that *AdeS* inactivation leads to aminoglycoside susceptibility with Thr153 Met mutations downstream from the putative His-149 site of autophosphorylation, probably leading to the loss of phosphorylase activity by the sensor (Marchand et al., 2004). In the current analysis, the two deleterious mutants, *AdeS* D167N and *AdeS* P172L, showed a correlation with carbapenem susceptibility. *AdeS* D167N was located at the last position of an alpha-4 helix and the following coil on which *AdeS* P172L was located. Contrarily, *AdeS* G186V was located in the middle of an alpha helix and probably at the boundary of a binding pocket (Supplementary Figure S8B). This substitution was conserved in tigecycline-resistant isolates in other studies (Sun et al., 2014). Although its role was not clear, it was proposed that previous selective pressure resulting from the use of different antibiotic agents, including carbapenem, was related to the emergence of tigecycline resistance.

I175L and H195Q were mapped on the available 3D structure of *AdeR* from PDB (5X5L) for the C-terminal DNA-binding domain for the positions 139-247. Supplementary Figure S9A shows only chain A and chain E for illustration purposes. I175L was identified in the middle of an alpha helix, probably affecting the DNA-binding pocket, and H195Q was also located near the end of another small alpha helix closely related to the DNA-binding pocket (Supplementary Figure S9A).

*AdeR* A53V was the only variant identified in the response regulator receiver domain. Mapped on the predicted structure for that domain, this amino acid substitution occurred near the end of the alpha-2 helix. It appears that it is located as a part of the magnesium-binding motif with a role in the phosphorylation of *AdeR*. Thus, it may be correlated with the expression of the efflux pump *AdeABC*. ALA 53 forms a polar interaction of 2.2 A with Ile 49 (Supplementary Figure S9B). The change of the ALA 53 to VAL leads to an additional 2.7 A bonding with GLU 50.

A133T and A136V are another two variants located in between the two domains of *AdeR*. A133T was located at the end of an alpha helix, and A136V was located at the start of the coil connecting the two domains (Supplementary Figure S10A). ALA 133 forms a polar interaction of 2 A with ARG 129, while ALA 136 forms a polar interaction of 2.9 with ASN 138 (Supplementary Figure S10B). Mutating ALA 136 to VAL leads to an additional bond of 2.5 A to ALA 133. Additional mutation of ALA 133 to THR leads to additional 2.2 bonding to VAL 136, probably adding more stability (Supplementary Figure S10B).

### 4.3. Role of membrane proteins and related variants

Previous studies have demonstrated the contribution of outer membrane channel loss or mutation, including OprD and CarO, to the carbapenem-resistance phenotype in *Acinetobacter* (Morán-Barrio et al., 2017). Although genetically engineered experimental mutants of *A. baumannii* with mutated CarO, Omp33, and OprD have shown almost similar MICs (<2-fold difference) compared to parenteral strains, Δ*ompA* mutants were more susceptible to both imipenem and meropenem when compared to parenteral strains (2–3 fold difference) (Tsai et al., 2020). In addition, similar findings showed <2-fold change in imipenem MIC with Δ*ompA* mutant compared to the wild-type strain (Kwon et al., 2017). This slight change in imipenem and meropenem susceptibility resulting from OmpA changes was supported by the finding in the current analysis, which showed lower specificity and PPVs for the OmpA_V1 variant observed (Table 3). This contrasted the significant increase in meropenem resistance, which was observed (>4-fold difference) with single *AdeRS* mutations. However, a <2-fold increase in resistance was observed with Δ*AdeAB*. Δ*AdeH* and Δ*AdeL* were also shown to have similar results (Tsai et al., 2020).

Large-scale quantitative proteomics analysis demonstrated that membrane proteins, including each of OmpA, CarO, and OmpW, were more abundant in MDR isolates (Chopra et al., 2013). Another study showed that the overexpression of genes encoding RND pumps—especially the *AdeFGH* pump—and the downregulation of porin-encoding genes are common in clinical CRAB isolates (Fernando et al., 2013). Studies have also shown that imipenem exposure can notably increase the expression levels of different RND efflux pumps and PBPs and decrease the levels of OmpW, OmpA, and CarO to prevent antibiotic import (Yun et al., 2011). In addition, recent proteomic and transcriptional analyses have identified OmpA and OmpW among the major proteins related to extensive drug resistance (Lee et al., 2018). In the current study, OmpW appeared to be highly conserved in the studied set, and although the variant OmpW T18A was observed, it did not show a significant correlation to carbapenem-resistance phenotypes.

Loss of a 43-kDa protein identified as a homolog of the OprD protein in *P. aeruginosa* has been previously linked to carbapenem resistance (Perez et al., 2007). Contrasting results did not correlate CarO, OprD, and 33-36Kda Omp expression to carbapenem resistance and showed identical outer membrane protein profiles among the studied panel (D'Souza et al., 2019). However, these observations may be deviated by the type of studied isolates. In the current set of studied isolates, two OprD variants correlated significantly with carbapenem susceptibility, and despite being observed in fewer isolates, both OprD_V2 and OprD_V3 showed high specificity to meropenem- and imipenem-susceptibility behavior, with values of 97.7 and 99.5%, respectively (Table 3). However, Omp33_V2 showed a significant correlation to the imipenem-susceptibility phenotype, while Omp33_V3 showed a significant correlation to the meropenem-susceptibility phenotype, with Omp33_V3 showing high specificity (96.6%) (Table 3).

The absence of the 29-kDa OMP (CarO) and 33-kDa outer membrane proteins has been previously associated with carbapenem resistance in *A. baumannii* (Limansky et al., 2002). In addition, Yang et al. (2015) showed CarO absence in 2 out of 27 studied MDR isolates. From similar findings, we can speculate that the channel would be changed due to mutations in the membrane porin, and this could result in resistance either by totally preventing antibiotic entry or by decreasing its amounts. However, another study from Brazil concluded that OXA-23 carbapenemase activity is the major carbapenem resistance mechanism among 28 studied isolates and that CarO loss plays only a minor role. However, the same study could associate different CarO alleles and their levels of expression with imipenem resistance, highlighting the complexity of understanding the carbapenem-resistance phenotype (Fonseca et al., 2013). Our findings that showed either the absence of a significant correlation or low predictive values of the studied CarO variants toward both meropenem- and imipenem-susceptibility phenotypes support these latter findings.

The glucose-selective porin OprB, the outer membrane protein A precursor, and the 33–36 kDa outer membrane proteins were among the major outer membrane proteins downregulated in imipenem culture conditions (Lee et al., 2014). Two variants located in the carbohydrate-selective porin domain OprB showed significant correlations to carbapenem-susceptibility behavior (Table 3).

In this analysis, OprB F57L was significantly correlated with meropenem susceptibility, showing high specificity and a predicted large decrease in protein stability. The variant is located in the middle of the first beta strand, which has an extracellular location near the protein N-terminal (Supplementary Figure S11). In *Pseudomonas*, position 56 was described as highly conserved in OprB orthologs and is located at strand S2, lining the porin channel with its side chain (van den Berg, 2012). From the current findings, it appears that the change of the bulky phenylalanine with an unreactive aromatic ring to leucine at position 57 might facilitate meropenem-binding and entry through the porin channel. However, the exact mechanism needs further study.

On the other hand, OprB G403S showed a significant correlation with both imipenem and meropenem resistance with high specificity and PPVs (Table 3). Functional prediction indicated that the variant is associated with a large decrease in protein stability. It is located in a coil preceding the last beta strand in the protein structure and has a cytoplasmic location near the C-terminal, as predicted using Psipred (Supplementary Figure S11). Position 402 was described among the highly conserved residues in OprB orthologs and is located in Loop L8, allowing a tight turn (van den Berg, 2012).

It can be concluded that, among membrane proteins, both OmpA and CarO have a lower contribution to the change in carbapenem resistance, and both OprB and OprD variants show a greater effect.

However, a study by Sen and Joshi (2016) concluded the absence of a correlation between each of OprD, *AdeABC*, and *AdeIJK* efflux pumps and carbapenem resistance based on expression data results. Their results may be deviated and non-generalizable because the study only included 16 carbapenem-resistant isolates, which might have greatly underestimated the contribution of these mechanisms. However, other research reports have shown that the combination of carbapenemases, efflux-pump expression, and the absence of outer membrane proteins contribute to carbapenem resistance in *A. baumannii* (Alcántar-Curiel et al., 2014).

### 4.4. Role of penicillin-binding protein variants

PBPs are an important family of enzymes inactivated by B-lactam drugs, including carbapenems. Recognizing and exploiting variations in these proteins is an essential approach in new antibiotic drug discovery and engineering to meet the growing challenge of multidrug-resistant Gram-negatives. Several studies have reported the role of PBP3 mutations in meropenem resistance (Hawkey et al., 2018; Nelson et al., 2020). The role of other PBPs in imipenem resistance has also been previously demonstrated (Cayô et al., 2011).

Researchers have reported that both PBP1a and PBP3 have unique roles in drug-resistant *A. baumannii* and could potentially underlie therapeutic failure (Kang and Boll, 2022). The analysis of variants observed in these proteins is, therefore, an extremely vital topic. The essentiality of PBP3 for the survival of *A. baumannii* has been recently emphasized (Toth et al., 2022). In our study, the variant PBP3_H370Y showed a significant correlation with the imipenem-susceptibility phenotype, showing very high specificity and PPV (Table 4). This position occurs near the drug-binding site, showing hydrophobic interactions, as observed in previous studies (Han et al., 2011; Papp-Wallace et al., 2012). Given the results of previous studies (Toth et al., 2022), in addition to the results of our study, it can be concluded that the amino acid position 370 of PBP3 may act as a potential drug target that can be exploited to counteract imipenem resistance in *A. baumannii*.

In the same study (Toth et al., 2022), the researchers demonstrated that the inactivation of PBP1a results in bacterial growth retardation and an increase in susceptibility to B-lactam. This was further enhanced by the additional inactivation of PBP2, resulting in a 4-fold increase in B-lactam antibiotic susceptibility. A similar trend can be concluded from the results of this study, where the PBP1a_T636A variant associated with a large decrease in stability can act as a potential drug target together with PBP1a_S382N, both acting as markers of imipenem susceptibility and showing high specificity and positive predictive values (Table 4).

Mapping PBP1a_S382N to the PBP1a 3D structure (ID = 3UDX) showed that the variant occurs at the transpeptidase (TP) domain, whose inactivation was associated with increased B-lactam susceptibility observed in other studies (Toth et al., 2022). Both the statistical and functional evidence reported in this study, as well as additional experimental evidence from other studies (Kang and Boll, 2022; Toth et al., 2022), make these positions potentially important drug targets.

Researchers have recently reported that a decreased affinity of PBP2 to carbapenems is one of the best described target site changes in *A. baumannii* (Martínez-Trejo et al., 2022). The lack of PBP2 was one of the most common mechanisms reported in CRAB isolates (Fernández-Cuenca et al., 2003), and these facts can be supported by the findings of our study. The results from the current study show that the variant PBP2_K152Q, predicted to cause a large decrease in protein stability, was significantly correlated with meropenem resistance, showing both high specificity and high PPV (Table 4).

Based on the PsiPred output for predicting the 3D structure, *MtgA*_L18F was located in the middle of the first alpha helix, which is a transmembrane helix showing membrane interaction (Supplementary Figure S12). Mapping the amino acid changes on the AlphaFold predicted 3D structure of *MtgA* showed that L18 forms three hydrogen bonds of 2.9 A, 2.9 A, and 3.4 A, respectively, within the containing alpha helix. While the F residue contains an unreactive aromatic ring, mutating the hydrophobic residue L into the bulky F residue may affect the protein-membrane interaction. *MtgA*_N122K is located in the middle of a coil between two alpha helices in the PBP transpeptidase fold that belongs to the biosynthetic peptidoglycan transglycosylase-like superfamily (Supplementary Figure S12). The change of the N residue to the K residue may affect post-translational modification because asparagine side chains are known to be major sites of post-translational modification. *MtgA*_E225Q was located at the end of a predicted coil at the end of the protein that is located near the center of the folded protein structure (Supplementary Figure S12). The change of glutamic acid to glutamine may affect its metal ion chelating activity.

### 4.5. Variants in relation to the genetic background

Figure 4 shows that *AdeB* A551T, *AdeB* L730F, *AdeR* A136V, *AdeS* G186V, and *AdeS* V348I co-occurred in Clusters 1, 2, and 3. All of these variants showed a significant correlation with meropenem resistance, while only *AdeS* G186V showed a significant correlation to both imipenem and meropenem resistance and was expected to be deleterious. The same panel was also observed in Cluster 9.

It is interesting to observe that *AdeR* I175L was only identified in Cluster 4 across the entire phylogram, and both *AdeS* I331V and *AdeN* K216N showed the largest occurrence in this cluster. *AdeS* I331V showed a significant correlation to imipenem resistance, while both *AdeR* I175L and *AdeN* K216N showed a significant correlation to both imipenem and meropenem resistance. Both showed high specificity (Table 1).

Cluster 5 appeared to be more recently acquired, and it included a unique cluster of variants occurring together. These were nearly only identified in this cluster across the entire phylogram. These included *AdeR* H158L, *AdeS* L105F, *AdeS* H189Y, *AdeS* V235I, and *AdeI* A388T. In addition, both *AdeN* P92T and *AdeB* G283A occurred exclusively at the lower subcluster, showing very high specificity and PPVs to both imipenem and meropenem resistance (Table 1). Each of *AdeN* P92T, *AdeB* G283A, *AdeR* H158L, *AdeS* H189Y, and *AdeI* A388T showed a significant correlation to both imipenem and meropenem resistance, while both *AdeS* L105F and *AdeS* V235I showed a significant correlation to meropenem resistance.

However, Cluster 6 showed the preserved occurrence of the variants *AdeA* A391T, *AdeB* T584I, *AdeC* S71G, *AdeC* K235E, *AdeC* Q239L, *AdeR* L142I, *AdeR* V243I, *AdeS* D167N, *AdeG* D501N, and *AdeL* Q262R among mostly carbapenem-susceptible isolates.

Each of *AdeA* A391T, *AdeC* S71G, *AdeR* L142I, *AdeR* V243I, and *AdeS* D167N showed a significant correlation to both imipenem and meropenem susceptibility, while each of *AdeB* T584I, *AdeC* K235E, *AdeC* Q239L, *AdeG* D501N, and *AdeL* Q262R were significantly correlated to imipenem susceptibility.

*AdeJ* V573M occurred only in Cluster 7 and showed a significant correlation to imipenem susceptibility with high specificity (Table 2). It occurred together with *AdeL* Q262R, which showed a significant correlation to imipenem susceptibility. However, other efflux variants from the same cluster did not correlate with susceptibility.

Each of *AdeB* D642A, *AdeB* S645T, *AdeC* S71G, *AdeC* T106M, *AdeC* K161S, *AdeH* R100G, *AdeH* A289G, and *AdeH* N388H co-occurred in Cluster 8 and showed a significant correlation with carbapenem susceptibility.

From Figure 5, it was observed that the three most recent clusters regarding the time of isolate collection were Clusters 1–3. Cluster 1 showed the co-occurrence of Omp_V1, CarO3, and PBP6_N329S, in addition to *MtgA*_T49P and *MtgA*_V54I. The isolates in this cluster were collected from different parts of China over the period between 2011 and 2013. Most of the clusters included respiratory samples, and the cluster showed both imipenem and meropenem resistance. The isolates in Cluster 2 were collected from Sweden and Thailand between 2011 and 2016 and similarly showed the co-occurrence of Omp_V1, CarO3, and PBP6_N329S, in addition to *MtgA*_T49P and *MtgA*_V54I. This cluster also showed imipenem and meropenem resistance; however, the isolates were isolated from different types of samples, including respiratory, wound, sterile body sites, and other infections. Imipenem-resistant Cluster 3 was observed in South Korea in blood isolates obtained in 2012 and showed the occurrence of the same panel listed in Clusters 1 and 2, in addition to PBP78_S32T. Out of these variants, Omp_V1 and PBP6_N329S showed a significant correlation to meropenem resistance, while both Omp_V1 and PBP78_S32T showed a significant correlation to imipenem resistance.

It can be observed that *MtgA*_T49P and *MtgA*_V54I are the most commonly occurring variants among the presented membrane-protein variants; however, both were nearly co-absent in a large cluster of related isolates, with nearly all isolates collected from Washington between 2003 and 2010 (Cluster 4). The cluster showed the co-occurrence of Omp_V1, CarO1, PBP1b_S112P, PBP2_K152Q, PBP6b_S28P, and PBP78_T71A. It is interesting to observe that PBP2_K152Q was only identified in this cluster and showed a significant correlation to both imipenem and meropenem resistance with high specificity (Table 5). Omp_V1 showed a significant correlation to both imipenem and meropenem resistance, while CarO1 showed a correlation to both imipenem and meropenem resistance (only significant with imipenem). Additionally, each of PBP1b_S112P, PBP6b_S28P, and PBP78_T71A showed a significant correlation to meropenem resistance.

*MtgA*_V54I was also not observed in another two related clusters from the USA (2003-2007) and from Sweden, Thailand, and Germany (2008-2013) (Cluster 5).

Another large cluster isolated from Washington (2003–2008) showed the co-occurrence of Omp33_V2, CarO3, PBP1a_S382N, PBP1a_T363A, PBP1b_P764S, PBP1b_S112P, PBP3_G523V, PBP2_V509I, PBP6b_S28P, and PBP78_T71A, in addition to *MtgA*_T49P and MtgA_V54I (Cluster 6). Among these, the variants Omp33_V2, PBP1a_S382N, PBP1a_T636A, PBP1b_P764S, PBP3_G523V, and PBP2_V509I occurred only in this cluster across the entire phylogram and were significantly related to carbapenem susceptibility.

The variant PBP3_H370Y was only observed across the whole phylogramin in Cluster 7 (shown in blue). It showed a significant correlation to imipenem susceptibility with very high specificity and PPV (Table 5). Isolates from this cluster were isolated from either wounds or sterile body sites and were isolated in Washington between 2003 and 2007. Most of these isolates were imipenem susceptible. Among the variants occurring in the same cluster, each of *MtgA*_T49P, *MtgA*_V54I, and Omp33_V2 showed a significant correlation to imipenem susceptibility.

Cluster 8 showed an interesting observation. The four variants Omp33_V3, OprD_V2, OprB_F57L, and *MtgA*_F4V were observed almost exclusively in cluster 8 across the entire phylogram. These four variants showed a significant correlation to meropenem susceptibility. Although genetically related, the cluster contained isolates collected from different parts of the USA, China, and India. It also spanned 10 years between 2003 and 2013 and was collected from different types of specimens, including respiratory infection, wound infection, abdominal fluid, blood, and groin, in addition to sterile body sites, which might indicate the more informative and predictive value of these variants.

## 5. Conclusion

Considerably more is known about efflux systems in *Ps. aeruginosa* and *E. coli* than about those in *Acinetobacter* spp. Most efflux pump systems possess physiological functions related to the homeostasis of the bacterial cell. An improved understanding of these systems, including the comprehension of their regulation and, particularly, of the RND family, can help us decode their natural functions and offer approaches to block or alter these pieces of machinery. It is also obvious that a complex network of regulations directs the efflux capacity of bacteria and that interplay or compensatory mechanisms between efflux systems could exist. It is worth noting that both membrane proteins and efflux-pump gene changes, when compared to other carbapenem-related elements of resistance, were not linked to phenotypic heteroresistance as in the case of other mobile elements (Fernández Cuenca et al., 2012), which may enforce their more essential and stable role in relation to carbapenem resistance.

From the predictive capacity and genetic relatedness analysis, it can be concluded that the following combinations occur together to predict carbapenem resistance:

1. The combination of *AdeN* P92T, *AdeB* G283A, *AdeR* H158L, *AdeI* A388T, *AdeS* H189Y, *AdeS* L105F, and *AdeS* V235I.
2. The combination of *AdeR* I175L, *AdeS* I331V, *AdeN* K216N, Omp_V1, CarO1, PBP1b_S112P, PBP2_K152Q, PBP6b_S28P, and PBP78_T71A, with higher predictive values especially for efflux pumps.
3. The combination of *AdeB* A551T, *AdeB* L730F, *AdeR* A136V, *AdeS* G186V, *AdeS* V348I, CarO3, Omp_V1, and PBP6_N329S co-occurs and is correlated with carbapenem resistance (significantly with meropenem resistance), with lower predictive values.

However, other combinations occur together and can be used to predict carbapenem susceptibility.

1. The combination of *AdeA* A391T, *AdeB* T584I, *AdeC* S71G, *AdeC* K235E, *AdeC* Q239L, *AdeR* L142I, *AdeR* V243I, *AdeS* D167N, *AdeG* D501N, and *AdeL* Q262R from efflux-pump variants and Omp33_V2, PBP1a_S382N, PBP1a_T636A, PBP1b_P764S, PBP3_G523V, and PBP2_V509I from membrane-protein variants.
2. The combination of *AdeB* D642A, *AdeB* S645T, *AdeC* S71G, *AdeC* T106M, *AdeC* K161S, *AdeH* R100G, *AdeH* A289G, and *AdeH* N388H from efflux-pump variants and Omp33_V3, OprD_V2, OprB_F57L, and *MtgA*_F4V from membrane-protein variants.

Carbapenem resistance is currently widespread worldwide in ICUs in different parts of the world; however, the currently available rapid diagnostic panels often depend on the detection of carbapenemases to predict carbapenem resistance and usually do not differentiate antibiotic agents from the same antimicrobial class. This approach is apparently lacking and incomplete as it disregards important chromosomal resistance predictors. Based on the results we presented in this study, the identified sets of markers can act as useful diagnostic predictor panels for inclusion in sequence-based diagnostic workflows.

The variants identified in this study can act as potential diagnostic markers that can be used to guide the clinical prescription of carbapenems, which would preserve the utility and effectiveness of such an antimicrobial class, especially in high-risk settings. The elements identified can also provide potential valuable targets for designing agents that might restore carbapenem susceptibility.

## Data availability statement

The original contributions presented in the study are included in the article/Supplementary material, further inquiries can be directed to the corresponding author.

## Author contributions

WN: Conceptualization, Formal analysis, Investigation, Methodology, Resources, Visualization, Writing—original draft, Writing—review and editing, Supervision. NA: Funding acquisition, Investigation, Writing—original draft, Methodology, Resources, Validation. AA: Funding acquisition, Investigation, Writing—original draft, Resources, Validation, Visualization. SZ: Funding acquisition, Investigation, Writing—review and editing, Methodology, Resources, Validation. AE: Writing—original draft, Visualization. HH: Formal analysis, Investigation, Resources, Supervision, Writing—original draft, Writing—review and editing, Validation.
