# Supplementary material for: Global genomic epidemiology of chromosomally mediated non-enzymatic carbapenem resistance in Acinetobacter baumannii: on the way to predict and modify resistance
Source: Front Microbiol. 2023 Oct 6;14:1271733. doi: 10.3389/fmicb.2023.1271733 (PMC10587612; doi:10.3389/fmicb.2023.1271733)
Supplement: Supplementary file 1 [file Data_Sheet_1.pdf]

Supplementary Tables

Global genomic epidemiology of chromosomally mediated non-enzymatic carbapenem resistance in *Acinetobacter baumannii* : On the way to predict and modify resistance

Wedad M. Nageeb [1*], Nada AlHarbi [2], Amani A. Alrehaili [3] , Shadi A. Zakai [4] , Ahmed Elfadadny [5] , Helal F. Hetta [6]

[1] Department of Medical Microbiology and Immunology, Faculty of Medicine, Suez Canal University, Ismailia, Egypt
[2] Department of Biology, Princess Nourah bint Abdulrahman University, College of Science, Riyadh, Saudi Arabia

[3] Department of Clinical Laboratory Sciences, College of Applied Medical Sciences, Taif University, Taif, Saudi Arabia

[4] Department of Clinical Microbiology and Immunology, Faculty of Medicine, King Abdulaziz University, Jeddah, Saudi Arabia

[5] Department of Animal Internal Medicine, Faculty of Veterinary Medicine, Damanhour University, Damanhour, El-Beheira, Egypt

[6] Department of Medical Microbiology and Immunology, Faculty of Medicine , Assiut University, Assiut, Egypt

*** Correspondence:**
Wedad M. Nageeb
wedad_saleh@med.suez.edu.eg

S1 Table: Genome names and IDs of studied whole genome sequences

Genome ID	Genome Name
470.1304	Acinetobacter baumannii strain 2011SDAB1
470.1305	Acinetobacter baumannii strain 2011SDAB2
470.1306	Acinetobacter baumannii strain 2005LNAB4
470.1307	Acinetobacter baumannii strain 2004ZJAB5
470.1308	Acinetobacter baumannii strain 2011LNAB2
470.1309	Acinetobacter baumannii strain 2011BJAB1
470.1359	Acinetobacter baumannii strain MRSN16897
470.1548	Acinetobacter baumannii strain MRSN17592
470.1549	Acinetobacter baumannii strain MRSN17593
470.1550	Acinetobacter baumannii strain MRSN16901
470.1551	Acinetobacter baumannii strain MRSN16896
470.1552	Acinetobacter baumannii strain MRSN16898
470.1553	Acinetobacter baumannii strain MRSN16881
470.1554	Acinetobacter baumannii strain MRSN16882
470.1555	Acinetobacter baumannii strain MRSN15038
470.1556	Acinetobacter baumannii strain MRSN20315
470.1557	Acinetobacter baumannii strain MRSN15084
470.1558	Acinetobacter baumannii strain MRSN20755
470.1559	Acinetobacter baumannii strain MRSN7081
470.1560	Acinetobacter baumannii strain MRSN16874
470.1561	Acinetobacter baumannii strain MRSN4119
470.1562	Acinetobacter baumannii strain MRSN3941
470.1563	Acinetobacter baumannii strain MRSN5540
470.1564	Acinetobacter baumannii strain MRSN6269
470.1565	Acinetobacter baumannii strain MRSN6273
470.2142	Acinetobacter baumannii A069
470.2143	Acinetobacter baumannii A068
470.2155	Acinetobacter baumannii A085
470.2156	Acinetobacter baumannii A086
470.2157	Acinetobacter baumannii A087
470.2158	Acinetobacter baumannii A089
470.2159	Acinetobacter baumannii A091
470.2160	Acinetobacter baumannii A092
470.2161	Acinetobacter baumannii A093
470.2162	Acinetobacter baumannii A094
470.2163	Acinetobacter baumannii A095

470.2165	Acinetobacter baumannii A097
470.2166	Acinetobacter baumannii A099
470.2167	Acinetobacter baumannii A100
470.2168	Acinetobacter baumannii A101
470.2425	Acinetobacter baumannii A070
470.2426	Acinetobacter baumannii A071
470.2427	Acinetobacter baumannii A072
470.2428	Acinetobacter baumannii A074
470.2429	Acinetobacter baumannii A076
470.2430	Acinetobacter baumannii A077
470.2431	Acinetobacter baumannii A078
470.2432	Acinetobacter baumannii A079
470.2433	Acinetobacter baumannii A080
470.2434	Acinetobacter baumannii A084
470.2435	Acinetobacter baumannii A082
470.2436	Acinetobacter baumannii A096
470.2437	Acinetobacter baumannii A105
470.2843	Acinetobacter baumannii strain AR_0036
470.2844	Acinetobacter baumannii strain AR_0037
470.2845	Acinetobacter baumannii strain AR_0033
470.2846	Acinetobacter baumannii strain AR_0035
470.2847	Acinetobacter baumannii strain AR_0045
470.2905	Acinetobacter baumannii strain 2011BJAB3
470.3764	Acinetobacter baumannii strain ARLG-1818
470.3765	Acinetobacter baumannii strain ARLG-1775
470.3766	Acinetobacter baumannii strain ARLG-1915
470.3767	Acinetobacter baumannii strain ARLG-1788
470.3768	Acinetobacter baumannii strain ARLG-1777
470.3769	Acinetobacter baumannii strain ARLG-1860
470.3770	Acinetobacter baumannii strain ARLG-1894
470.3771	Acinetobacter baumannii strain ARLG-1854
470.3772	Acinetobacter baumannii strain ARLG-1912
470.4063	Acinetobacter baumannii strain SIUA6
470.4064	Acinetobacter baumannii strain SIPA5
470.4065	Acinetobacter baumannii strain SIUA14
470.4066	Acinetobacter baumannii strain SIPA14
470.4701	Acinetobacter baumannii strain AR_0056
470.4702	Acinetobacter baumannii strain AR_0063
470.4704	Acinetobacter baumannii strain AR_0078

470.4748	Acinetobacter baumannii strain AR_0088
470.4785	Acinetobacter baumannii strain AR_0102
470.4786	Acinetobacter baumannii strain AR_0101
470.4881	Acinetobacter baumannii strain AR_0056
470.4901	Acinetobacter baumannii strain AR_0083
470.5040	Acinetobacter baumannii strain CCUG 70743
470.5410	Acinetobacter baumannii strain AR_0070
470.5421	Acinetobacter baumannii strain AR_0052
470.559	Acinetobacter baumannii 2011BJAB2
470.573	Acinetobacter baumannii 2011BJAB4
470.574	Acinetobacter baumannii 2011BJAB7
470.575	Acinetobacter baumannii 2004BJAB10
470.576	Acinetobacter baumannii 2003BJAB12
470.577	Acinetobacter baumannii 2004BJAB13
470.578	Acinetobacter baumannii 2004BJAB14
470.579	Acinetobacter baumannii 2011GDAB3
470.580	Acinetobacter baumannii 2005JSAB1
470.581	Acinetobacter baumannii 2011SHAB1
470.584	Acinetobacter baumannii 2011SDAB3
470.585	Acinetobacter baumannii 2011LNAB1
470.587	Acinetobacter baumannii 2011ZJAB1
470.589	Acinetobacter baumannii 2004ZJAB6
470.590	Acinetobacter baumannii 2011BJAB5
470.591	Acinetobacter baumannii 2011BJAB8
470.592	Acinetobacter baumannii 2011BJAB9
470.593	Acinetobacter baumannii 1999BJAB11
470.594	Acinetobacter baumannii 2011GDAB1
470.595	Acinetobacter baumannii 2011GDAB2
470.596	Acinetobacter baumannii 2011GDAB4
470.597	Acinetobacter baumannii 2011HNAB1
470.598	Acinetobacter baumannii 2011TJAB1
470.600	Acinetobacter baumannii 2011LNAB3
470.601	Acinetobacter baumannii 2011ZJAB2
470.602	Acinetobacter baumannii 2011ZJAB3
470.603	Acinetobacter baumannii 2011ZJAB4
470.604	Acinetobacter baumannii 2011BJAB6
470.7351	Acinetobacter baumannii strain BL02
470.7352	Acinetobacter baumannii strain BL03
470.7353	Acinetobacter baumannii strain BL04

470.7354	Acinetobacter baumannii strain BL05
470.7355	Acinetobacter baumannii strain BL06
470.7356	Acinetobacter baumannii strain BL07
470.7357	Acinetobacter baumannii strain BL08
470.7358	Acinetobacter baumannii strain BL09
470.7359	Acinetobacter baumannii strain BL10
470.7360	Acinetobacter baumannii strain BL11
470.7361	Acinetobacter baumannii strain BL12
470.7362	Acinetobacter baumannii strain BL13
470.7363	Acinetobacter baumannii strain BL14
470.7364	Acinetobacter baumannii strain BL15
470.7365	Acinetobacter baumannii strain BL16
470.7366	Acinetobacter baumannii strain BL17
470.7367	Acinetobacter baumannii strain BL18
470.7368	Acinetobacter baumannii strain BL19
470.7369	Acinetobacter baumannii strain BL20
470.7370	Acinetobacter baumannii strain BL21
470.7371	Acinetobacter baumannii strain BL22
470.7372	Acinetobacter baumannii strain BL23
470.7373	Acinetobacter baumannii strain BL24
470.7374	Acinetobacter baumannii strain FDA-CDC-AR_0273
470.7375	Acinetobacter baumannii strain FDA-CDC-AR_0274
470.7376	Acinetobacter baumannii strain FDA-CDC-AR_0275
470.7377	Acinetobacter baumannii strain FDA-CDC-AR_0277
470.7378	Acinetobacter baumannii strain FDA-CDC-AR_0278
470.7379	Acinetobacter baumannii strain FDA-CDC-AR_0279
470.7380	Acinetobacter baumannii strain FDA-CDC-AR_0280
470.7381	Acinetobacter baumannii strain FDA-CDC-AR_0281
470.7382	Acinetobacter baumannii strain FDA-CDC-AR_0282
470.7383	Acinetobacter baumannii strain FDA-CDC-AR_0283
470.7384	Acinetobacter baumannii strain FDA-CDC-AR_0284
470.7385	Acinetobacter baumannii strain FDA-CDC-AR_0285
470.7386	Acinetobacter baumannii strain FDA-CDC-AR_0286
470.7387	Acinetobacter baumannii strain FDA-CDC-AR_0287
470.7388	Acinetobacter baumannii strain FDA-CDC-AR_0288
470.7389	Acinetobacter baumannii strain FDA-CDC-AR_0289
470.7390	Acinetobacter baumannii strain FDA-CDC-AR_0290
470.7391	Acinetobacter baumannii strain FDA-CDC-AR_0291
470.7392	Acinetobacter baumannii strain FDA-CDC-AR_0292

470.7393	Acinetobacter baumannii strain FDA-CDC-AR_0293
470.7394	Acinetobacter baumannii strain FDA-CDC-AR_0294
470.7395	Acinetobacter baumannii strain FDA-CDC-AR_0295
470.7396	Acinetobacter baumannii strain FDA-CDC-AR_0296
470.7397	Acinetobacter baumannii strain FDA-CDC-AR_0297
470.7398	Acinetobacter baumannii strain FDA-CDC-AR_0298
470.7399	Acinetobacter baumannii strain FDA-CDC-AR_0299
470.7400	Acinetobacter baumannii strain FDA-CDC-AR_0300
470.7401	Acinetobacter baumannii strain FDA-CDC-AR_0301
470.7402	Acinetobacter baumannii strain FDA-CDC-AR_0302
470.7403	Acinetobacter baumannii strain FDA-CDC-AR_0303
470.7404	Acinetobacter baumannii strain FDA-CDC-AR_0304
470.7405	Acinetobacter baumannii strain FDA-CDC-AR_0305
470.7406	Acinetobacter baumannii strain FDA-CDC-AR_0306
470.7407	Acinetobacter baumannii strain FDA-CDC-AR_0307
470.7408	Acinetobacter baumannii strain FDA-CDC-AR_0308
470.7409	Acinetobacter baumannii strain FDA-CDC-AR_0309
470.7410	Acinetobacter baumannii strain FDA-CDC-AR_0310
470.7411	Acinetobacter baumannii strain FDA-CDC-AR_0311
470.7412	Acinetobacter baumannii strain FDA-CDC-AR_0312
470.7413	Acinetobacter baumannii strain FDA-CDC-AR_0313
470.7414	Acinetobacter baumannii strain MRSN10191
470.7415	Acinetobacter baumannii strain MRSN10371
470.7416	Acinetobacter baumannii strain MRSN10372
470.7417	Acinetobacter baumannii strain MRSN11650
470.7418	Acinetobacter baumannii strain MRSN11653
470.7419	Acinetobacter baumannii strain MRSN11659
470.7420	Acinetobacter baumannii strain MRSN11660
470.7421	Acinetobacter baumannii strain MRSN11669
470.7422	Acinetobacter baumannii strain MRSN11671
470.7423	Acinetobacter baumannii strain MRSN11674
470.7424	Acinetobacter baumannii strain MRSN11675
470.7425	Acinetobacter baumannii strain MRSN11679
470.7426	Acinetobacter baumannii strain MRSN11680
470.7427	Acinetobacter baumannii strain MRSN11683
470.7428	Acinetobacter baumannii strain MRSN11684
470.7429	Acinetobacter baumannii strain MRSN11687
470.7430	Acinetobacter baumannii strain MRSN11688
470.7431	Acinetobacter baumannii strain MRSN11690

470.7432	Acinetobacter baumannii strain MRSN11695
470.7433	Acinetobacter baumannii strain MRSN11698
470.7434	Acinetobacter baumannii strain MRSN11700
470.7435	Acinetobacter baumannii strain MRSN11701
470.7436	Acinetobacter baumannii strain MRSN11705
470.7437	Acinetobacter baumannii strain MRSN11706
470.7438	Acinetobacter baumannii strain MRSN11707
470.7439	Acinetobacter baumannii strain MRSN11708
470.7440	Acinetobacter baumannii strain MRSN11709
470.7441	Acinetobacter baumannii strain MRSN11710
470.7442	Acinetobacter baumannii strain MRSN11712
470.7443	Acinetobacter baumannii strain MRSN11713
470.7444	Acinetobacter baumannii strain MRSN11714
470.7445	Acinetobacter baumannii strain MRSN11716
470.7446	Acinetobacter baumannii strain MRSN11720
470.7447	Acinetobacter baumannii strain MRSN11721
470.7448	Acinetobacter baumannii strain MRSN11722
470.7449	Acinetobacter baumannii strain MRSN11723
470.7450	Acinetobacter baumannii strain MRSN11725
470.7451	Acinetobacter baumannii strain MRSN11726
470.7452	Acinetobacter baumannii strain MRSN11727
470.7453	Acinetobacter baumannii strain MRSN11729
470.7454	Acinetobacter baumannii strain MRSN11732
470.7455	Acinetobacter baumannii strain MRSN11735
470.7456	Acinetobacter baumannii strain MRSN11736
470.7457	Acinetobacter baumannii strain MRSN11737
470.7458	Acinetobacter baumannii strain MRSN11738
470.7459	Acinetobacter baumannii strain MRSN11739
470.7460	Acinetobacter baumannii strain MRSN11742
470.7461	Acinetobacter baumannii strain MRSN11744
470.7462	Acinetobacter baumannii strain MRSN11745
470.7463	Acinetobacter baumannii strain MRSN11746
470.7464	Acinetobacter baumannii strain MRSN11747
470.7465	Acinetobacter baumannii strain MRSN11748
470.7466	Acinetobacter baumannii strain MRSN11749
470.7467	Acinetobacter baumannii strain MRSN11750
470.7468	Acinetobacter baumannii strain MRSN11751
470.7469	Acinetobacter baumannii strain MRSN11753
470.7470	Acinetobacter baumannii strain MRSN11756

470.7471	Acinetobacter baumannii strain MRSN11757
470.7472	Acinetobacter baumannii strain MRSN11759
470.7473	Acinetobacter baumannii strain MRSN11760
470.7474	Acinetobacter baumannii strain MRSN11761
470.7475	Acinetobacter baumannii strain MRSN11762
470.7476	Acinetobacter baumannii strain MRSN11763
470.7477	Acinetobacter baumannii strain MRSN11764
470.7478	Acinetobacter baumannii strain MRSN11765
470.7479	Acinetobacter baumannii strain MRSN11766
470.7480	Acinetobacter baumannii strain MRSN11767
470.7481	Acinetobacter baumannii strain MRSN11769
470.7482	Acinetobacter baumannii strain MRSN11770
470.7483	Acinetobacter baumannii strain MRSN11771
470.7484	Acinetobacter baumannii strain MRSN11772
470.7485	Acinetobacter baumannii strain MRSN11774
470.7486	Acinetobacter baumannii strain MRSN11776
470.7487	Acinetobacter baumannii strain MRSN11777
470.7488	Acinetobacter baumannii strain MRSN11778
470.7489	Acinetobacter baumannii strain MRSN11780
470.7490	Acinetobacter baumannii strain MRSN11781
470.7491	Acinetobacter baumannii strain MRSN11783
470.7492	Acinetobacter baumannii strain MRSN11784
470.7493	Acinetobacter baumannii strain MRSN11788
470.7494	Acinetobacter baumannii strain MRSN11790
470.7495	Acinetobacter baumannii strain MRSN11792
470.7496	Acinetobacter baumannii strain MRSN11793
470.7497	Acinetobacter baumannii strain MRSN11795
470.7498	Acinetobacter baumannii strain MRSN11796
470.7499	Acinetobacter baumannii strain MRSN11797
470.7500	Acinetobacter baumannii strain MRSN11801
470.7501	Acinetobacter baumannii strain MRSN11803
470.7502	Acinetobacter baumannii strain MRSN11804
470.7503	Acinetobacter baumannii strain MRSN11806
470.7504	Acinetobacter baumannii strain MRSN11807
470.7505	Acinetobacter baumannii strain MRSN11811
470.7506	Acinetobacter baumannii strain MRSN11812
470.7507	Acinetobacter baumannii strain MRSN11813
470.7508	Acinetobacter baumannii strain MRSN11815
470.7509	Acinetobacter baumannii strain MRSN11816

470.751	Acinetobacter baumannii ABUH45562
470.7511	Acinetobacter baumannii strain MRSN11820
470.7512	Acinetobacter baumannii strain MRSN11821
470.7513	Acinetobacter baumannii strain MRSN11822
470.7514	Acinetobacter baumannii strain MRSN11823
470.7515	Acinetobacter baumannii strain MRSN11825
470.7516	Acinetobacter baumannii strain MRSN11826
470.7517	Acinetobacter baumannii strain MRSN1308
470.7518	Acinetobacter baumannii strain MRSN1309
470.7519	Acinetobacter baumannii strain MRSN1339
470.752	Acinetobacter baumannii ABUH45561
470.7521	Acinetobacter baumannii strain MRSN1420
470.7522	Acinetobacter baumannii strain MRSN1428
470.7523	Acinetobacter baumannii strain MRSN1439
470.7524	Acinetobacter baumannii strain MRSN1449
470.7525	Acinetobacter baumannii strain MRSN1450
470.7526	Acinetobacter baumannii strain MRSN1461
470.7527	Acinetobacter baumannii strain MRSN15037
470.7528	Acinetobacter baumannii strain MRSN15038
470.7529	Acinetobacter baumannii strain MRSN15039
470.753	Acinetobacter baumannii ABUH31558
470.7531	Acinetobacter baumannii strain MRSN15043
470.7532	Acinetobacter baumannii strain MRSN15044
470.7533	Acinetobacter baumannii strain MRSN15045
470.7534	Acinetobacter baumannii strain MRSN15046
470.7535	Acinetobacter baumannii strain MRSN15047
470.7536	Acinetobacter baumannii strain MRSN15048
470.7537	Acinetobacter baumannii strain MRSN15064
470.7538	Acinetobacter baumannii strain MRSN15065
470.7539	Acinetobacter baumannii strain MRSN15066
470.754	Acinetobacter baumannii ABUH24357
470.7541	Acinetobacter baumannii strain MRSN15068
470.7542	Acinetobacter baumannii strain MRSN15070
470.7543	Acinetobacter baumannii strain MRSN15071
470.7544	Acinetobacter baumannii strain MRSN15072
470.7545	Acinetobacter baumannii strain MRSN15073
470.7546	Acinetobacter baumannii strain MRSN15075
470.7547	Acinetobacter baumannii strain MRSN15076
470.7548	Acinetobacter baumannii strain MRSN15078

470.7549	Acinetobacter baumannii strain MRSN15079
470.755	Acinetobacter baumannii ABUH24356
470.7551	Acinetobacter baumannii strain MRSN15081
470.7552	Acinetobacter baumannii strain MRSN15082
470.7553	Acinetobacter baumannii strain MRSN15083
470.7554	Acinetobacter baumannii strain MRSN15084
470.7555	Acinetobacter baumannii strain MRSN15085
470.7556	Acinetobacter baumannii strain MRSN15086
470.7557	Acinetobacter baumannii strain MRSN15087
470.7558	Acinetobacter baumannii strain MRSN1551
470.7559	Acinetobacter baumannii strain MRSN15088
470.756	Acinetobacter baumannii ABUH24355
470.7561	Acinetobacter baumannii strain MRSN1738
470.7562	Acinetobacter baumannii strain MRSN1966
470.7563	Acinetobacter baumannii strain MRSN1967
470.7564	Acinetobacter baumannii strain MRSN1971
470.7565	Acinetobacter baumannii strain MRSN1973
470.7566	Acinetobacter baumannii strain MRSN1980
470.7567	Acinetobacter baumannii strain MRSN1987
470.7568	Acinetobacter baumannii strain MRSN1988
470.7569	Acinetobacter baumannii strain MRSN1992
470.757	Acinetobacter baumannii ABUH24354
470.7571	Acinetobacter baumannii strain MRSN2009
470.7572	Acinetobacter baumannii strain MRSN2021
470.7573	Acinetobacter baumannii strain MRSN2024
470.7574	Acinetobacter baumannii strain MRSN2427
470.7575	Acinetobacter baumannii strain MRSN2441
470.7576	Acinetobacter baumannii strain MRSN2821
470.7577	Acinetobacter baumannii strain MRSN2830
470.7578	Acinetobacter baumannii strain MRSN2839
470.7579	Acinetobacter baumannii strain MRSN2840
470.758	Acinetobacter baumannii ABUH1752
470.7581	Acinetobacter baumannii strain MRSN3547
470.7582	Acinetobacter baumannii strain MRSN3607
470.7583	Acinetobacter baumannii strain MRSN3843
470.7584	Acinetobacter baumannii strain MRSN3851
470.7585	Acinetobacter baumannii strain MRSN4481
470.7586	Acinetobacter baumannii strain MRSN7068
470.7587	Acinetobacter baumannii strain MRSN7069

470.7588	Acinetobacter baumannii strain MRSN7070
470.7589	Acinetobacter baumannii strain MRSN7072
470.759	Acinetobacter baumannii ABUH24343
470.7591	Acinetobacter baumannii strain MRSN7074
470.7592	Acinetobacter baumannii strain MRSN7079
470.7593	Acinetobacter baumannii strain MRSN7084
470.7594	Acinetobacter baumannii strain MRSN7080
470.7595	Acinetobacter baumannii strain MRSN7086
470.7596	Acinetobacter baumannii strain MRSN7087
470.7597	Acinetobacter baumannii strain MRSN7088
470.7598	Acinetobacter baumannii strain MRSN7091
470.7599	Acinetobacter baumannii strain MRSN7097
470.7600	Acinetobacter baumannii strain MRSN7099
470.7601	Acinetobacter baumannii strain MRSN7100
470.7602	Acinetobacter baumannii strain MRSN7102
470.7603	Acinetobacter baumannii strain MRSN7108
470.7604	Acinetobacter baumannii strain MRSN7111
470.7605	Acinetobacter baumannii strain MRSN7112
470.7606	Acinetobacter baumannii strain MRSN7114
470.7607	Acinetobacter baumannii strain MRSN7115
470.7608	Acinetobacter baumannii strain MRSN7116
470.7609	Acinetobacter baumannii strain MRSN7117
470.761	Acinetobacter baumannii ABUH31539
470.7611	Acinetobacter baumannii strain MRSN7119
470.7612	Acinetobacter baumannii strain MRSN7122
470.7613	Acinetobacter baumannii strain MRSN7124
470.7614	Acinetobacter baumannii strain MRSN7125
470.7615	Acinetobacter baumannii strain MRSN7126
470.7616	Acinetobacter baumannii strain MRSN7127
470.7617	Acinetobacter baumannii strain MRSN7128
470.7618	Acinetobacter baumannii strain MRSN7129
470.7619	Acinetobacter baumannii strain MRSN7130
470.762	Acinetobacter baumannii ABUH45538
470.7621	Acinetobacter baumannii strain MRSN7132
470.7622	Acinetobacter baumannii strain MRSN7133
470.7623	Acinetobacter baumannii strain MRSN7134
470.7624	Acinetobacter baumannii strain MRSN7137
470.7625	Acinetobacter baumannii strain MRSN7138
470.7626	Acinetobacter baumannii strain MRSN7139

470.7627	Acinetobacter baumannii strain MRSN7140
470.7628	Acinetobacter baumannii strain MRSN7142
470.7629	Acinetobacter baumannii strain MRSN7143
470.763	Acinetobacter baumannii ABUH24333
470.7631	Acinetobacter baumannii strain MRSN7146
470.7632	Acinetobacter baumannii strain MRSN7147
470.7633	Acinetobacter baumannii strain MRSN7148
470.7634	Acinetobacter baumannii strain MRSN7149
470.7635	Acinetobacter baumannii strain MRSN7150
470.7636	Acinetobacter baumannii strain MRSN7153
470.7637	Acinetobacter baumannii strain MRSN7154
470.7638	Acinetobacter baumannii strain MRSN7155
470.7639	Acinetobacter baumannii strain MRSN7156
470.764	Acinetobacter baumannii ABUH34827
470.7641	Acinetobacter baumannii strain MRSN7158
470.7642	Acinetobacter baumannii strain MRSN7159
470.7643	Acinetobacter baumannii strain MRSN7160
470.7644	Acinetobacter baumannii strain MRSN7161
470.7645	Acinetobacter baumannii strain MRSN7162
470.7646	Acinetobacter baumannii strain MRSN7163
470.7647	Acinetobacter baumannii strain MRSN7164
470.7648	Acinetobacter baumannii strain MRSN7165
470.7649	Acinetobacter baumannii strain MRSN7166
470.765	Acinetobacter baumannii ABUH34825
470.7651	Acinetobacter baumannii strain MRSN7168
470.7652	Acinetobacter baumannii strain MRSN7169
470.7653	Acinetobacter baumannii strain MRSN7170
470.7654	Acinetobacter baumannii strain MRSN7171
470.7655	Acinetobacter baumannii strain MRSN7172
470.7656	Acinetobacter baumannii strain MRSN7173
470.7657	Acinetobacter baumannii strain MRSN7175
470.7658	Acinetobacter baumannii strain MRSN7181
470.7659	Acinetobacter baumannii strain MRSN7183
470.766	Acinetobacter baumannii ABUH24314
470.7661	Acinetobacter baumannii strain MRSN7185
470.7662	Acinetobacter baumannii strain MRSN7186
470.7663	Acinetobacter baumannii strain MRSN7188
470.7664	Acinetobacter baumannii strain MRSN7189
470.7665	Acinetobacter baumannii strain MRSN7202

470.7666	Acinetobacter baumannii strain MRSN7204
470.7667	Acinetobacter baumannii strain MRSN7206
470.7668	Acinetobacter baumannii strain MRSN7207
470.7669	Acinetobacter baumannii strain MRSN7208
470.767	Acinetobacter baumannii ABUH34813
470.7671	Acinetobacter baumannii strain MRSN7211
470.7672	Acinetobacter baumannii strain MRSN7212
470.7673	Acinetobacter baumannii strain MRSN7214
470.7674	Acinetobacter baumannii strain MRSN7216
470.7675	Acinetobacter baumannii strain MRSN7217
470.7676	Acinetobacter baumannii strain MRSN7218
470.7677	Acinetobacter baumannii strain MRSN7219
470.7678	Acinetobacter baumannii strain MRSN7220
470.7679	Acinetobacter baumannii strain MRSN7221
470.768	Acinetobacter baumannii ABUH4556
470.7681	Acinetobacter baumannii strain MRSN7223
470.7682	Acinetobacter baumannii strain MRSN7224
470.7683	Acinetobacter baumannii strain MRSN7226
470.7684	Acinetobacter baumannii strain MRSN7227
470.7685	Acinetobacter baumannii strain MRSN7228
470.7686	Acinetobacter baumannii strain MRSN7229
470.7687	Acinetobacter baumannii strain MRSN7231
470.7688	Acinetobacter baumannii strain MRSN7232
470.7689	Acinetobacter baumannii strain MRSN7235
470.7690	Acinetobacter baumannii strain MRSN7234
470.7691	Acinetobacter baumannii strain MRSN7236
470.7692	Acinetobacter baumannii strain MRSN7237
470.7693	Acinetobacter baumannii strain MRSN7238
470.7694	Acinetobacter baumannii strain MRSN7239
470.7695	Acinetobacter baumannii strain MRSN7240
470.7696	Acinetobacter baumannii strain MRSN7241
470.7697	Acinetobacter baumannii strain MRSN7243
470.7698	Acinetobacter baumannii strain MRSN7245
470.7699	Acinetobacter baumannii strain MRSN7247
470.7700	Acinetobacter baumannii strain MRSN7248
470.7701	Acinetobacter baumannii strain MRSN7249
470.7702	Acinetobacter baumannii strain MRSN7250
470.7703	Acinetobacter baumannii strain MRSN7251
470.7704	Acinetobacter baumannii strain MRSN7252

470.7705	Acinetobacter baumannii strain MRSN7253
470.7706	Acinetobacter baumannii strain MRSN7254
470.7707	Acinetobacter baumannii strain MRSN7255
470.7708	Acinetobacter baumannii strain MRSN7256
470.7709	Acinetobacter baumannii strain MRSN7257
470.771	Acinetobacter baumannii AbH12O-A2
470.7711	Acinetobacter baumannii strain MRSN7259
470.7712	Acinetobacter baumannii strain MRSN7261
470.7713	Acinetobacter baumannii strain MRSN7263
470.7714	Acinetobacter baumannii strain MRSN7262
470.7715	Acinetobacter baumannii strain MRSN7267
470.7716	Acinetobacter baumannii strain MRSN7269
470.7717	Acinetobacter baumannii strain MRSN7270
470.7718	Acinetobacter baumannii strain MRSN7271
470.7719	Acinetobacter baumannii strain MRSN7272
470.7720	Acinetobacter baumannii strain MRSN7273
470.7721	Acinetobacter baumannii strain MRSN7274
470.7722	Acinetobacter baumannii strain MRSN7277
470.7723	Acinetobacter baumannii strain MRSN7278
470.7724	Acinetobacter baumannii strain MRSN7279
470.7725	Acinetobacter baumannii strain MRSN7280
470.7726	Acinetobacter baumannii strain MRSN7281
470.7727	Acinetobacter baumannii strain MRSN7282
470.7728	Acinetobacter baumannii strain MRSN7284
470.7729	Acinetobacter baumannii strain MRSN7285
470.773	Acinetobacter baumannii 6200
470.7731	Acinetobacter baumannii strain MRSN7289
470.7732	Acinetobacter baumannii strain MRSN7290
470.7733	Acinetobacter baumannii strain MRSN7291
470.7734	Acinetobacter baumannii strain MRSN7293
470.7735	Acinetobacter baumannii strain MRSN7294
470.7736	Acinetobacter baumannii strain MRSN7295
470.7737	Acinetobacter baumannii strain MRSN7296
470.7738	Acinetobacter baumannii strain MRSN7297
470.7739	Acinetobacter baumannii strain MRSN7298
470.774	Acinetobacter baumannii IOMTU 433
470.7741	Acinetobacter baumannii strain MRSN7301
470.7742	Acinetobacter baumannii strain MRSN7303
470.7743	Acinetobacter baumannii strain MRSN7304

470.7744	Acinetobacter baumannii strain MRSN7305
470.7745	Acinetobacter baumannii strain MRSN7306
470.7746	Acinetobacter baumannii strain MRSN7308
470.7747	Acinetobacter baumannii strain MRSN7309
470.7748	Acinetobacter baumannii strain MRSN7310
470.7749	Acinetobacter baumannii strain MRSN7311
470.775	Acinetobacter baumannii A1
470.7751	Acinetobacter baumannii strain MRSN7314
470.7752	Acinetobacter baumannii strain MRSN7316
470.7753	Acinetobacter baumannii strain MRSN7318
470.7754	Acinetobacter baumannii strain MRSN7321
470.7755	Acinetobacter baumannii strain MRSN7322
470.7756	Acinetobacter baumannii strain MRSN7323
470.7757	Acinetobacter baumannii strain MRSN7324
470.7758	Acinetobacter baumannii strain MRSN7325
470.7759	Acinetobacter baumannii strain MRSN7326
470.7760	Acinetobacter baumannii strain MRSN7327
470.7761	Acinetobacter baumannii strain MRSN7328
470.7762	Acinetobacter baumannii strain MRSN7330
470.7763	Acinetobacter baumannii strain MRSN7331
470.7764	Acinetobacter baumannii strain MRSN7336
470.7765	Acinetobacter baumannii strain MRSN7338
470.7766	Acinetobacter baumannii strain MRSN7342
470.7767	Acinetobacter baumannii strain MRSN7343
470.7768	Acinetobacter baumannii strain MRSN7344
470.7769	Acinetobacter baumannii strain MRSN7345
470.7770	Acinetobacter baumannii strain MRSN7346
470.7771	Acinetobacter baumannii strain MRSN7347
470.7772	Acinetobacter baumannii strain MRSN7348
470.7773	Acinetobacter baumannii strain MRSN7349
470.7774	Acinetobacter baumannii strain MRSN7350
470.7775	Acinetobacter baumannii strain MRSN7351
470.7776	Acinetobacter baumannii strain MRSN7352
470.7777	Acinetobacter baumannii strain MRSN7353
470.7778	Acinetobacter baumannii strain MRSN7354
470.7779	Acinetobacter baumannii strain MRSN7355
470.7780	Acinetobacter baumannii strain MRSN7356
470.7781	Acinetobacter baumannii strain MRSN7357
470.7782	Acinetobacter baumannii strain MRSN7359

470.7783	Acinetobacter baumannii strain MRSN7360
470.7784	Acinetobacter baumannii strain MRSN7361
470.7785	Acinetobacter baumannii strain MRSN7362
470.7786	Acinetobacter baumannii strain MRSN7363
470.7787	Acinetobacter baumannii strain MRSN7364
470.7788	Acinetobacter baumannii strain MRSN7365
470.7789	Acinetobacter baumannii strain MRSN7366
470.7790	Acinetobacter baumannii strain MRSN7367
470.7791	Acinetobacter baumannii strain MRSN7368
470.7792	Acinetobacter baumannii strain MRSN7370
470.7793	Acinetobacter baumannii strain MRSN7372
470.7794	Acinetobacter baumannii strain MRSN7373
470.7795	Acinetobacter baumannii strain MRSN7375
470.7796	Acinetobacter baumannii strain MRSN7376
470.7797	Acinetobacter baumannii strain MRSN7377
470.7798	Acinetobacter baumannii strain MRSN7378
470.7799	Acinetobacter baumannii strain MRSN7379
470.7800	Acinetobacter baumannii strain MRSN7380
470.7801	Acinetobacter baumannii strain MRSN7381
470.7802	Acinetobacter baumannii strain MRSN7382
470.7803	Acinetobacter baumannii strain MRSN7383
470.7804	Acinetobacter baumannii strain MRSN7385
470.7805	Acinetobacter baumannii strain MRSN7386
470.7806	Acinetobacter baumannii strain MRSN7387
470.7807	Acinetobacter baumannii strain MRSN7388
470.7808	Acinetobacter baumannii strain MRSN7389
470.7809	Acinetobacter baumannii strain MRSN7390
470.7810	Acinetobacter baumannii strain MRSN7391
470.7811	Acinetobacter baumannii strain MRSN7392
470.7812	Acinetobacter baumannii strain MRSN7393
470.7813	Acinetobacter baumannii strain MRSN7394
470.7814	Acinetobacter baumannii strain MRSN7397
470.7815	Acinetobacter baumannii strain MRSN7398
470.7816	Acinetobacter baumannii strain MRSN7401
470.7817	Acinetobacter baumannii strain MRSN7402
470.7818	Acinetobacter baumannii strain MRSN7403
470.7819	Acinetobacter baumannii strain MRSN7404
470.7820	Acinetobacter baumannii strain MRSN7405
470.7821	Acinetobacter baumannii strain MRSN7406

470.7822	Acinetobacter baumannii strain MRSN7407
470.7823	Acinetobacter baumannii strain MRSN7408
470.7824	Acinetobacter baumannii strain MRSN7409
470.7825	Acinetobacter baumannii strain MRSN7410
470.7826	Acinetobacter baumannii strain MRSN7411
470.7827	Acinetobacter baumannii strain MRSN7412
470.7828	Acinetobacter baumannii strain MRSN7413
470.7829	Acinetobacter baumannii strain MRSN7414
470.7830	Acinetobacter baumannii strain MRSN7415
470.7831	Acinetobacter baumannii strain MRSN7416
470.7832	Acinetobacter baumannii strain MRSN7417
470.7833	Acinetobacter baumannii strain MRSN7418
470.7834	Acinetobacter baumannii strain MRSN7419
470.7835	Acinetobacter baumannii strain MRSN7420
470.7836	Acinetobacter baumannii strain MRSN7421
470.7837	Acinetobacter baumannii strain MRSN7423
470.7838	Acinetobacter baumannii strain MRSN7424
470.7839	Acinetobacter baumannii strain MRSN7434
470.7840	Acinetobacter baumannii strain MRSN7435
470.7841	Acinetobacter baumannii strain MRSN7436
470.7842	Acinetobacter baumannii strain MRSN7437
470.7843	Acinetobacter baumannii strain MRSN7438
470.7844	Acinetobacter baumannii strain MRSN7440
470.7845	Acinetobacter baumannii strain MRSN7441
470.7846	Acinetobacter baumannii strain MRSN7446
470.7847	Acinetobacter baumannii strain MRSN7447
470.7848	Acinetobacter baumannii strain MRSN7448
470.7849	Acinetobacter baumannii strain MRSN7454
470.7850	Acinetobacter baumannii strain MRSN7455
470.7851	Acinetobacter baumannii strain MRSN7456
470.7852	Acinetobacter baumannii strain MRSN7457
470.7853	Acinetobacter baumannii strain MRSN7458
470.7854	Acinetobacter baumannii strain MRSN7460
470.7855	Acinetobacter baumannii strain MRSN7459
470.7856	Acinetobacter baumannii strain MRSN7461
470.7857	Acinetobacter baumannii strain MRSN7462
470.7858	Acinetobacter baumannii strain MRSN7463
470.7859	Acinetobacter baumannii strain MRSN7465
470.7860	Acinetobacter baumannii strain MRSN7467

470.7861	Acinetobacter baumannii strain MRSN7468
470.7862	Acinetobacter baumannii strain MRSN7469
470.7863	Acinetobacter baumannii strain MRSN7470
470.7864	Acinetobacter baumannii strain MRSN7471
470.7865	Acinetobacter baumannii strain MRSN7472
470.7866	Acinetobacter baumannii strain MRSN7474
470.7867	Acinetobacter baumannii strain MRSN7475
470.7868	Acinetobacter baumannii strain MRSN7477
470.7869	Acinetobacter baumannii strain MRSN7478
470.7870	Acinetobacter baumannii strain MRSN7479
470.7871	Acinetobacter baumannii strain MRSN7481
470.7872	Acinetobacter baumannii strain MRSN7482
470.7873	Acinetobacter baumannii strain MRSN7483
470.7874	Acinetobacter baumannii strain MRSN7484
470.7875	Acinetobacter baumannii strain MRSN7485
470.7876	Acinetobacter baumannii strain MRSN7486
470.7877	Acinetobacter baumannii strain MRSN7487
470.7878	Acinetobacter baumannii strain MRSN7488
470.7879	Acinetobacter baumannii strain MRSN7489
470.7880	Acinetobacter baumannii strain MRSN7491
470.7881	Acinetobacter baumannii strain MRSN7492
470.7882	Acinetobacter baumannii strain MRSN7495
470.7883	Acinetobacter baumannii strain MRSN7497
470.7884	Acinetobacter baumannii strain MRSN7498
470.7885	Acinetobacter baumannii strain MRSN7499
470.7886	Acinetobacter baumannii strain MRSN7500
470.7887	Acinetobacter baumannii strain MRSN7503
470.7888	Acinetobacter baumannii strain MRSN7504
470.7889	Acinetobacter baumannii strain MRSN7505
470.7890	Acinetobacter baumannii strain MRSN7506
470.7891	Acinetobacter baumannii strain MRSN7508
470.7892	Acinetobacter baumannii strain MRSN7509
470.7893	Acinetobacter baumannii strain MRSN7510
470.7894	Acinetobacter baumannii strain MRSN7511
470.7895	Acinetobacter baumannii strain MRSN7512
470.7896	Acinetobacter baumannii strain MRSN7514
470.7897	Acinetobacter baumannii strain MRSN7515
470.7898	Acinetobacter baumannii strain MRSN7518
470.7899	Acinetobacter baumannii strain MRSN7517

470.7900	Acinetobacter baumannii strain MRSN7520
470.7901	Acinetobacter baumannii strain MRSN7521
470.7902	Acinetobacter baumannii strain MRSN7524
470.7903	Acinetobacter baumannii strain MRSN7525
470.7904	Acinetobacter baumannii strain MRSN7526
470.7905	Acinetobacter baumannii strain MRSN7531
470.7906	Acinetobacter baumannii strain MRSN7532
470.7907	Acinetobacter baumannii strain MRSN7535
470.7908	Acinetobacter baumannii strain MRSN7539
470.7909	Acinetobacter baumannii strain MRSN7543
470.7910	Acinetobacter baumannii strain MRSN7544
470.7911	Acinetobacter baumannii strain MRSN7545
470.7912	Acinetobacter baumannii strain MRSN7547
470.7913	Acinetobacter baumannii strain MRSN7548
470.7914	Acinetobacter baumannii strain MRSN7549
470.7915	Acinetobacter baumannii strain MRSN7550
470.7916	Acinetobacter baumannii strain MRSN7551
470.7917	Acinetobacter baumannii strain MRSN7555
470.7918	Acinetobacter baumannii strain MRSN7557
470.7919	Acinetobacter baumannii strain MRSN7558
470.7920	Acinetobacter baumannii strain MRSN7560
470.7921	Acinetobacter baumannii strain MRSN7563
470.7922	Acinetobacter baumannii strain MRSN7564
470.7923	Acinetobacter baumannii strain MRSN7565
470.7924	Acinetobacter baumannii strain MRSN7566
470.7925	Acinetobacter baumannii strain MRSN7567
470.7926	Acinetobacter baumannii strain MRSN7568
470.7927	Acinetobacter baumannii strain MRSN7569
470.7928	Acinetobacter baumannii strain MRSN7570
470.7929	Acinetobacter baumannii strain MRSN7571
470.7930	Acinetobacter baumannii strain MRSN7574
470.7931	Acinetobacter baumannii strain MRSN7576
470.7932	Acinetobacter baumannii strain MRSN7577
470.7933	Acinetobacter baumannii strain MRSN7578
470.7934	Acinetobacter baumannii strain MRSN7579
470.7935	Acinetobacter baumannii strain MRSN7580
470.7936	Acinetobacter baumannii strain MRSN7582
470.7937	Acinetobacter baumannii strain MRSN7585
470.7938	Acinetobacter baumannii strain MRSN7586

470.7939	Acinetobacter baumannii strain MRSN7588
470.7940	Acinetobacter baumannii strain MRSN7590
470.7941	Acinetobacter baumannii strain MRSN7591
470.7942	Acinetobacter baumannii strain MRSN7593
470.7943	Acinetobacter baumannii strain MRSN7594
470.7944	Acinetobacter baumannii strain MRSN7597
470.7945	Acinetobacter baumannii strain MRSN7598
470.7946	Acinetobacter baumannii strain MRSN7599
470.7947	Acinetobacter baumannii strain MRSN7600
470.7948	Acinetobacter baumannii strain MRSN7604
470.7949	Acinetobacter baumannii strain MRSN7605
470.7950	Acinetobacter baumannii strain MRSN7607
470.7951	Acinetobacter baumannii strain MRSN7610
470.7952	Acinetobacter baumannii strain MRSN7611
470.7953	Acinetobacter baumannii strain MRSN7612
470.7954	Acinetobacter baumannii strain MRSN7617
470.7955	Acinetobacter baumannii strain MRSN7618
470.7956	Acinetobacter baumannii strain MRSN7619
470.7957	Acinetobacter baumannii strain MRSN7626
470.7958	Acinetobacter baumannii strain MRSN7627
470.7959	Acinetobacter baumannii strain MRSN7628
470.7960	Acinetobacter baumannii strain MRSN7629
470.7961	Acinetobacter baumannii strain MRSN7631
470.7962	Acinetobacter baumannii strain MRSN7636
470.7963	Acinetobacter baumannii strain MRSN7637
470.7964	Acinetobacter baumannii strain MRSN7639
470.7965	Acinetobacter baumannii strain MRSN7640
470.7966	Acinetobacter baumannii strain MRSN7642
470.7967	Acinetobacter baumannii strain MRSN7643
470.7968	Acinetobacter baumannii strain MRSN7644
470.7969	Acinetobacter baumannii strain MRSN7646
470.7970	Acinetobacter baumannii strain MRSN7647
470.7971	Acinetobacter baumannii strain MRSN7648
470.7972	Acinetobacter baumannii strain MRSN7649
470.7973	Acinetobacter baumannii strain MRSN7653
470.7974	Acinetobacter baumannii strain MRSN7654
470.7975	Acinetobacter baumannii strain MRSN7655
470.7976	Acinetobacter baumannii strain MRSN7657
470.7977	Acinetobacter baumannii strain MRSN7658

470.7978	Acinetobacter baumannii strain MRSN7659
470.7979	Acinetobacter baumannii strain MRSN7660
470.7980	Acinetobacter baumannii strain MRSN7662
470.7981	Acinetobacter baumannii strain MRSN7667
470.7982	Acinetobacter baumannii strain MRSN7668
470.7983	Acinetobacter baumannii strain MRSN7669
470.7984	Acinetobacter baumannii strain MRSN7670
470.7985	Acinetobacter baumannii strain MRSN7671
470.7986	Acinetobacter baumannii strain MRSN7672
470.7987	Acinetobacter baumannii strain MRSN7673
470.7988	Acinetobacter baumannii strain MRSN7674
470.7989	Acinetobacter baumannii strain MRSN7675
470.7990	Acinetobacter baumannii strain MRSN7676
470.7991	Acinetobacter baumannii strain MRSN7677
470.7992	Acinetobacter baumannii strain MRSN7678
470.7993	Acinetobacter baumannii strain MRSN7679
470.7994	Acinetobacter baumannii strain MRSN7683
470.7995	Acinetobacter baumannii strain MRSN7684
470.7996	Acinetobacter baumannii strain MRSN7686
470.7997	Acinetobacter baumannii strain MRSN7689
470.7998	Acinetobacter baumannii strain MRSN7690
470.7999	Acinetobacter baumannii strain MRSN7693
470.8000	Acinetobacter baumannii strain MRSN7694
470.8001	Acinetobacter baumannii strain MRSN7695
470.8002	Acinetobacter baumannii strain MRSN7697
470.8003	Acinetobacter baumannii strain MRSN7698
470.8004	Acinetobacter baumannii strain MRSN7699
470.8005	Acinetobacter baumannii strain MRSN7701
470.8006	Acinetobacter baumannii strain MRSN7703
470.8007	Acinetobacter baumannii strain MRSN7706
470.8008	Acinetobacter baumannii strain MRSN7707
470.8009	Acinetobacter baumannii strain MRSN7709
470.8010	Acinetobacter baumannii strain MRSN7710
470.8011	Acinetobacter baumannii strain MRSN7711
470.8012	Acinetobacter baumannii strain MRSN7712
470.8013	Acinetobacter baumannii strain MRSN7713
470.8014	Acinetobacter baumannii strain MRSN7714
470.8015	Acinetobacter baumannii strain MRSN7716
470.8016	Acinetobacter baumannii strain MRSN7717

470.8017	Acinetobacter baumannii strain MRSN7718
470.8018	Acinetobacter baumannii strain MRSN7720
470.8019	Acinetobacter baumannii strain MRSN7722
470.8020	Acinetobacter baumannii strain MRSN7723
470.8021	Acinetobacter baumannii strain MRSN7724
470.8022	Acinetobacter baumannii strain MRSN7725
470.8023	Acinetobacter baumannii strain MRSN7726
470.8024	Acinetobacter baumannii strain MRSN7730
470.8025	Acinetobacter baumannii strain MRSN7731
470.8026	Acinetobacter baumannii strain MRSN7735
470.8027	Acinetobacter baumannii strain MRSN7740
470.8028	Acinetobacter baumannii strain MRSN7745
470.8029	Acinetobacter baumannii strain MRSN7748
470.8030	Acinetobacter baumannii strain MRSN7749
470.8031	Acinetobacter baumannii strain MRSN7751
470.8032	Acinetobacter baumannii strain MRSN7752
470.8033	Acinetobacter baumannii strain MRSN7753
470.8034	Acinetobacter baumannii strain MRSN7756
470.8035	Acinetobacter baumannii strain MRSN7759
470.8036	Acinetobacter baumannii strain MRSN7760
470.8037	Acinetobacter baumannii strain MRSN7761
470.8038	Acinetobacter baumannii strain MRSN7767
470.8039	Acinetobacter baumannii strain MRSN7768
470.8040	Acinetobacter baumannii strain MRSN7769
470.8041	Acinetobacter baumannii strain MRSN7776
470.8042	Acinetobacter baumannii strain MRSN7777
470.8043	Acinetobacter baumannii strain MRSN7779
470.8044	Acinetobacter baumannii strain MRSN7780
470.8045	Acinetobacter baumannii strain MRSN7782
470.8046	Acinetobacter baumannii strain MRSN7787
470.8047	Acinetobacter baumannii strain MRSN7790
470.8048	Acinetobacter baumannii strain MRSN7791
470.8049	Acinetobacter baumannii strain MRSN7792
470.8050	Acinetobacter baumannii strain MRSN7794
470.8051	Acinetobacter baumannii strain MRSN7797
470.8052	Acinetobacter baumannii strain MRSN7798
470.8053	Acinetobacter baumannii strain MRSN7799
470.8054	Acinetobacter baumannii strain MRSN7805
470.8055	Acinetobacter baumannii strain MRSN7806

470.8056	Acinetobacter baumannii strain MRSN7807
470.8057	Acinetobacter baumannii strain MRSN7811
470.8058	Acinetobacter baumannii strain MRSN7814
470.8059	Acinetobacter baumannii strain MRSN7815
470.8060	Acinetobacter baumannii strain MRSN7816
470.8061	Acinetobacter baumannii strain MRSN7817
470.8062	Acinetobacter baumannii strain MRSN7818
470.8063	Acinetobacter baumannii strain MRSN7819
470.8064	Acinetobacter baumannii strain MRSN7821
470.8065	Acinetobacter baumannii strain MRSN7822
470.8066	Acinetobacter baumannii strain MRSN7824
470.8067	Acinetobacter baumannii strain MRSN7828
470.8068	Acinetobacter baumannii strain MRSN7829
470.8069	Acinetobacter baumannii strain MRSN7831
470.8070	Acinetobacter baumannii strain MRSN7832
470.8071	Acinetobacter baumannii strain MRSN7834
470.8072	Acinetobacter baumannii strain MRSN83
470.8073	Acinetobacter baumannii strain MRSN846
470.8074	Acinetobacter baumannii strain MRSN85
470.8075	Acinetobacter baumannii strain MRSN867
470.8076	Acinetobacter baumannii strain MRSN870
470.8077	Acinetobacter baumannii strain MRSN885
470.8078	Acinetobacter baumannii strain MRSN886
470.8079	Acinetobacter baumannii strain MRSN888
470.8080	Acinetobacter baumannii strain MRSN889
470.8081	Acinetobacter baumannii strain MRSN8899
470.8082	Acinetobacter baumannii strain MRSN89
470.8083	Acinetobacter baumannii strain MRSN890
470.8084	Acinetobacter baumannii strain MRSN8900
470.8085	Acinetobacter baumannii strain MRSN8901
470.8086	Acinetobacter baumannii strain MRSN8902
470.8087	Acinetobacter baumannii strain MRSN8903
470.8088	Acinetobacter baumannii strain MRSN891
470.8089	Acinetobacter baumannii strain MRSN896
470.8090	Acinetobacter baumannii strain MRSN899
470.8091	Acinetobacter baumannii strain MRSN902
470.8092	Acinetobacter baumannii strain MRSN908
470.8093	Acinetobacter baumannii strain MRSN909
470.8094	Acinetobacter baumannii strain MRSN910

470.8095	Acinetobacter baumannii strain MRSN915
470.8096	Acinetobacter baumannii strain MRSN916
470.8097	Acinetobacter baumannii strain MRSN917
470.8098	Acinetobacter baumannii strain MRSN919
470.8099	Acinetobacter baumannii strain MRSN920
470.8100	Acinetobacter baumannii strain MRSN921
470.8101	Acinetobacter baumannii strain MRSN922
470.8102	Acinetobacter baumannii strain MRSN926
470.8103	Acinetobacter baumannii strain MRSN927
470.8104	Acinetobacter baumannii strain MRSN929
470.8105	Acinetobacter baumannii strain MRSN939
470.8106	Acinetobacter baumannii strain MRSN946
470.8107	Acinetobacter baumannii strain MRSN953
470.8108	Acinetobacter baumannii strain MRSN966
470.8109	Acinetobacter baumannii strain SIUA3
470.9021	Acinetobacter baumannii strain MRSN31196
470.9022	Acinetobacter baumannii strain MRSN31461
470.9023	Acinetobacter baumannii strain MRSN31468
470.9024	Acinetobacter baumannii strain MRSN30909
470.9025	Acinetobacter baumannii strain MRSN5969
470.9026	Acinetobacter baumannii strain MRSN960
470.9027	Acinetobacter baumannii strain MRSN7690
470.9028	Acinetobacter baumannii strain MRSN959
470.9029	Acinetobacter baumannii strain MRSN489678
470.9030	Acinetobacter baumannii strain MRSN7213
470.9031	Acinetobacter baumannii strain MRSN489669
470.9032	Acinetobacter baumannii strain MRSN4484
470.9033	Acinetobacter baumannii strain MRSN843
470.9034	Acinetobacter baumannii strain MRSN918
470.9035	Acinetobacter baumannii strain MRSN7521
470.9036	Acinetobacter baumannii strain MRSN7251
470.9037	Acinetobacter baumannii strain MRSN6541
470.9038	Acinetobacter baumannii strain MRSN32142
470.9039	Acinetobacter baumannii strain MRSN334
470.9040	Acinetobacter baumannii strain MRSN480561
470.9041	Acinetobacter baumannii strain MRSN7153
470.9042	Acinetobacter baumannii strain MRSN4943
470.9043	Acinetobacter baumannii strain MRSN31915
470.9044	Acinetobacter baumannii strain MRSN31523

470.9045	Acinetobacter baumannii strain MRSN7124
470.9046	Acinetobacter baumannii strain MRSN7431
470.9047	Acinetobacter baumannii strain MRSN337038
470.9048	Acinetobacter baumannii strain MRSN7725
470.9049	Acinetobacter baumannii strain MRSN32892
470.9050	Acinetobacter baumannii strain MRSN32875
470.9051	Acinetobacter baumannii strain MRSN3658
470.9052	Acinetobacter baumannii strain MRSN7113
470.9053	Acinetobacter baumannii strain MRSN7576
470.9054	Acinetobacter baumannii strain MRSN32108
470.9055	Acinetobacter baumannii strain MRSN7067
470.9056	Acinetobacter baumannii strain MRSN480622
470.9057	Acinetobacter baumannii strain MRSN7460
470.9058	Acinetobacter baumannii strain MRSN423159
470.9059	Acinetobacter baumannii strain MRSN7137
470.9060	Acinetobacter baumannii strain MRSN32866
470.9061	Acinetobacter baumannii strain MRSN351524
470.9062	Acinetobacter baumannii strain MRSN3874
470.9063	Acinetobacter baumannii strain MRSN30912
470.9064	Acinetobacter baumannii strain MRSN32865
470.9065	Acinetobacter baumannii strain MRSN31947
470.9066	Acinetobacter baumannii strain MRSN3692
470.9067	Acinetobacter baumannii strain MRSN3360
470.9068	Acinetobacter baumannii strain MRSN32104
470.9069	Acinetobacter baumannii strain MRSN32842
470.9070	Acinetobacter baumannii strain MRSN7735
470.9071	Acinetobacter baumannii strain MRSN7446
470.9072	Acinetobacter baumannii strain MRSN351162
470.9073	Acinetobacter baumannii strain MRSN32915
470.9074	Acinetobacter baumannii strain MRSN32304
470.9075	Acinetobacter baumannii strain MRSN32797
470.9076	Acinetobacter baumannii strain MRSN31937
470.9077	Acinetobacter baumannii strain MRSN32076
470.9078	Acinetobacter baumannii strain MRSN31942
470.9079	Acinetobacter baumannii strain MRSN30945
470.9080	Acinetobacter baumannii strain MRSN11224
470.9081	Acinetobacter baumannii strain MRSN1174
470.9082	Acinetobacter baumannii strain MRSN11669
470.9083	Acinetobacter baumannii strain MRSN30000

470.9084	Acinetobacter baumannii strain MRSN19482
470.9085	Acinetobacter baumannii strain MRSN25547
470.9086	Acinetobacter baumannii strain MRSN29908
470.9087	Acinetobacter baumannii strain MRSN11695
470.9088	Acinetobacter baumannii strain MRSN24008
470.9089	Acinetobacter baumannii strain MRSN1551
470.9090	Acinetobacter baumannii strain MRSN2821
470.9091	Acinetobacter baumannii strain MRSN10372
470.9092	Acinetobacter baumannii strain MRSN24603
470.9093	Acinetobacter baumannii strain MRSN15088
470.9094	Acinetobacter baumannii strain MRSN1171
470.9095	Acinetobacter baumannii strain MRSN30885
470.9096	Acinetobacter baumannii strain MRSN29999
470.9097	Acinetobacter baumannii strain MRSN22112
470.9098	Acinetobacter baumannii strain MRSN11703
470.9099	Acinetobacter baumannii strain MRSN21660
470.9100	Acinetobacter baumannii strain MRSN15075
470.9101	Acinetobacter baumannii strain MRSN17493
470.9102	Acinetobacter baumannii strain MRSN30896
470.9103	Acinetobacter baumannii strain MRSN15129
470.9104	Acinetobacter baumannii strain MRSN15070
470.9105	Acinetobacter baumannii strain MRSN15049
470.9106	Acinetobacter baumannii strain MRSN1311
470.9107	Acinetobacter baumannii strain MRSN14427
470.9108	Acinetobacter baumannii strain MRSN14193
470.9109	Acinetobacter baumannii strain MRSN14237
470.9110	Acinetobacter baumannii strain MRSN1187
470.9111	Acinetobacter baumannii strain MRSN1196
470.9112	Acinetobacter baumannii strain MRSN11816
470.9113	Acinetobacter baumannii strain MRSN1183
470.9114	Acinetobacter baumannii strain MRSN15574
470.9115	Acinetobacter baumannii strain MRSN15093
470.9116	Acinetobacter baumannii strain MRSN16880
470.9117	Acinetobacter baumannii strain MRSN31159
470.9118	Acinetobacter baumannii strain MRSN11663
470.9120	Acinetobacter baumannii strain MRSN23390
470.9121	Acinetobacter baumannii strain MRSN21681
470.9341	Acinetobacter baumannii strain 5464
470.9342	Acinetobacter baumannii strain 34301

470.9343	Acinetobacter baumannii strain 5326
470.9344	Acinetobacter baumannii strain 2992
470.9345	Acinetobacter baumannii strain 20858
470.9346	Acinetobacter baumannii strain 29282
470.9347	Acinetobacter baumannii strain 2375
470.9348	Acinetobacter baumannii strain 16533
470.9411	Acinetobacter baumannii strain 5457
470.9416	Acinetobacter baumannii strain 6507

S2 Table: Summary of Efflux-pump variants and membrane proteins previously reported in the literature

**Variant**	**Reference**
AdeR [D20N, P116L, A91V, A136V, L192R]	Lari AR, Ardebili A, Hashemi A. AdeR-AdeS mutations & overexpression of the AdeABC efflux system in ciprofloxacin-resistant *Acinetobacter baumannii* clinical isolates. Indian J Med Res. 2018 Apr;147(4):413-421. doi: 10.4103/ijmr.IJMR_644_16. PMID: 29998878; PMCID: PMC6057251.
AdeS[G186V, T153M, G30D, A94V, G103D]	Lari AR, Ardebili A, Hashemi A. AdeR-AdeS mutations & overexpression of the AdeABC efflux system in ciprofloxacin-resistant Acinetobacter baumannii clinical isolates. Indian J Med Res. 2018 Apr;147(4):413-421. doi: 10.4103/ijmr.IJMR_644_16. PMID: 29998878; PMCID: PMC6057251.
AdeR[D20N, A91V and P116L]	Yu-Kuo Tsai, Ci-Hong Liou, Jung-Chung Lin, Chang-Phone Fung, Feng-Yee Chang, L. Kristopher Siu, Effects of different resistance mechanisms on antimicrobial resistance in Acinetobacter baumannii: a strategic system for screening and activity testing of new antibiotics,International Journal of Antimicrobial Agents,Volume 55, Issue 4,2020,105918,ISSN 0924-8579,https://doi.org/10.1016/j.ijantimicag.2020.105918.
AdeS[G30D, A94V, R152K and T153M]	
AdeS[Gly186Val]	Yu-Kuo Tsai, Ci-Hong Liou, Jung-Chung Lin, Chang-Phone Fung, Feng-Yee Chang, L. Kristopher Siu, Effects of different resistance mechanisms on antimicrobial resistance in Acinetobacter baumannii: a strategic system for screening and activity testing of new antibiotics,International Journal of Antimicrobial Agents,Volume 55, Issue 4,2020,105918,ISSN 0924-8579,https://doi.org/10.1016/j.ijantimicag.2020.105918.
AdeS[Thr 153 Met, Gly30Asp]	Marchand I, Damier-Piolle L, Courvalin P, Lambert T. Expression of the RND-type efflux pump AdeABC in Acinetobacter baumannii is regulated by the AdeRS two-component system. Antimicrob Agents Chemother. 2004 Sep;48(9):3298-304. doi: 10.1128/AAC.48.9.3298-3304.2004. PMID: 15328088; PMCID: PMC514774.
AdeR[Pro 116 Leu]	Marchand I, Damier-Piolle L, Courvalin P, Lambert T. Expression of the RND-type efflux pump AdeABC in Acinetobacter baumannii is regulated by the AdeRS two-component system. Antimicrob Agents Chemother. 2004 Sep;48(9):3298-304. doi: 10.1128/AAC.48.9.3298-3304.2004. PMID: 15328088; PMCID: PMC514774.
AdeS[N268H and G186A, L172P, Y303F,	Reem Hassan, Ahmed Mukhtar, Ahmed Hasanin & Doaa Ghaith (2018) Role of insertion sequence Aba-1 and AdeS in reduced tigecycline susceptibility in MDR-

A37V, P305A, G186A, S188A, V217E, I257N]	Acinetobacter baumannii clinical isolates from Cairo, Egypt, Journal of Chemotherapy, 30:2, 89-94, DOI: 10.1080/1120009X.2017.1396057
AdeS[G30D]	Coyne S, Guigon G, Courvalin P, Périchon B. Screening and quantification of the expression of antibiotic resistance genes in Acinetobacter baumannii with a microarray. Antimicrob Agents Chemother. 2010 Jan;54(1):333-40. doi: 10.1128/AAC.01037-09. Epub 2009 Nov 2. PMID: 19884373; PMCID: PMC2798560.
AdeR[D20N, A91V, P116L, P56S, L192R, E219A, I175M]	Yoon EJ, Courvalin P, Grillot-Courvalin C. RND-type efflux pumps in multidrug-resistant clinical isolates of Acinetobacter baumannii: major role for AdeABC overexpression and AdeRS mutations. Antimicrob Agents Chemother. 2013 Jul;57(7):2989-95. doi: 10.1128/AAC.02556-12. Epub 2013 Apr 15. PMID: 23587960; PMCID: PMC3697384.
AdeS[G103D, T153M, A94V, R152K, I252S, V27I, V32I, A94V, F214L, S280A, Q281D, Q299R, and I360M, N268H, W143R, D227H, and T337S]	Yoon EJ, Courvalin P, Grillot-Courvalin C. RND-type efflux pumps in multidrug-resistant clinical isolates of Acinetobacter baumannii: major role for AdeABC overexpression and AdeRS mutations. Antimicrob Agents Chemother. 2013 Jul;57(7):2989-95. doi: 10.1128/AAC.02556-12. Epub 2013 Apr 15. PMID: 23587960; PMCID: PMC3697384.
AdeR[Asn134-Lys, His195-Gln, Pro241-Leu, Val120-Ile, Ala136-Val, Ala133-Thr, Val243-Ile]	Sirawit Pagdepanichkit, Chanwit Tribuddharat, and Rungtip Chuanchuen. 2016. Distribution and expression of the Ade multidrug efflux systems in Acinetobacter baumannii clinical isolates. Canadian Journal of Microbiology. 62(9): 794-801. https://doi.org/10.1139/cjm-2015-0730
AdeS[Iso73-Val, Ala153-Thr, Leu214-Phe, Val245-Phe, Ser263-Ala, Val279-Ala, Ala280-Ser, Asp281-Gln, Val331-Ile, Ser354-Pro, Gly186-Val, Leu214-Phe, Asn268-Ser, Val348-Ile, Asp167-Tyr, Gly168-Cys, Gly41-Ala, Trp42-Gly, Ile43-Leu, Ser44-Val, Glu174-Lys, Ser341-Cys, Met355-Leu]	Sirawit Pagdepanichkit, Chanwit Tribuddharat, and Rungtip Chuanchuen. 2016. Distribution and expression of the Ade multidrug efflux systems in Acinetobacter baumannii clinical isolates. Canadian Journal of Microbiology. 62(9): 794-801. https://doi.org/10.1139/cjm-2015-0730
AdeR[E219 K]	Qingye Xu, Xiaoting Hua, Jintao He, Di Zhang, Qiong Chen, Linghong Zhang, Belinda Loh, Sebastian Leptihn, Yurong Wen, Paul G. Higgins, Yunsong Yu, Zhihui Zhou,<br>The distribution of mutations and hotspots in transcription regulators of resistance-nodulation-cell division efflux pumps in tigecycline non-susceptible Acinetobacter baumannii in China,International Journal of Medical

	Microbiology,Volume 310, Issue 8,2020,151464,ISSN 1438-4221,https://doi.org/10.1016/j.ijmm.2020.151464.
AdeS[A130 T, S8R , A130D]	Qingye Xu, Xiaoting Hua, Jintao He, Di Zhang, Qiong Chen, Linghong Zhang, Belinda Loh, Sebastian Leptihn, Yurong Wen, Paul G. Higgins, Yunsong Yu, Zhihui Zhou, The distribution of mutations and hotspots in transcription regulators of resistance-nodulation-cell division efflux pumps in tigecycline non-susceptible Acinetobacter baumannii in China,International Journal of Medical Microbiology,Volume 310, Issue 8,2020,151464,ISSN 1438-4221,https://doi.org/10.1016/j.ijmm.2020.151464.
AdeR[A136V and V120I, L16F]	Yilmaz Ş, Hasdemir U, Aksu B, Altınkanat Gelmez G, Söyletir G. Alterations in AdeS and AdeR regulatory proteins in 1-(1-naphthylmethyl)-piperazine responsive colistin resistance of *Acinetobacter baumannii*. J Chemother. 2020 Oct;32(6):286-293. doi: 10.1080/1120009X.2020.1735118. Epub 2020 Mar 5. PMID: 32131715.
AdeS[L172P, A94V, V27I, Q281D, V32I, G186V, and G164A]	Yilmaz Ş, Hasdemir U, Aksu B, Altınkanat Gelmez G, Söyletir G. Alterations in AdeS and AdeR regulatory proteins in 1-(1-naphthylmethyl)-piperazine responsive colistin resistance of *Acinetobacter baumannii*. J Chemother. 2020 Oct;32(6):286-293. doi: 10.1080/1120009X.2020.1735118. Epub 2020 Mar 5. PMID: 32131715.
AdeL[Arg2-Cys, Gln264-Arg, Asp227-Ala, Gln264-Arg]	Sirawit Pagdepanichkit, Chanwit Tribuddharat, and Rungtip Chuanchuen. 2016. Distribution and expression of the Ade multidrug efflux systems in Acinetobacter baumannii clinical isolates. Canadian Journal of Microbiology. 62(9): 794-801. https://doi.org/10.1139/cjm-2015-0730
AdeN[Met197-Thr, Met174-Thr,	Sirawit Pagdepanichkit, Chanwit Tribuddharat, and Rungtip Chuanchuen. 2016. Distribution and expression of the Ade multidrug efflux systems in Acinetobacter baumannii clinical isolates. Canadian Journal of Microbiology. 62(9): 794-801. https://doi.org/10.1139/cjm-2015-0730
AdeL[valine-to-glycine 139, threonine-to-lysine 319]	Coyne S, Rosenfeld N, Lambert T, Courvalin P, Périchon B. Overexpression of resistance-nodulation-cell division pump AdeFGH confers multidrug resistance in Acinetobacter baumannii. Antimicrob Agents Chemother. 2010 Oct;54(10):4389-93. doi: 10.1128/AAC.00155-10. Epub 2010 Aug 9. PMID: 20696879; PMCID: PMC2944555.
AdeB[F136L, G288S]	Hawkey J, Ascher DB, Judd LM, Wick RR, Kostoulias X, Cleland H, Spelman DW, Padiglione A, Peleg AY, Holt KE. Evolution of carbapenem resistance in Acinetobacter baumannii during a prolonged infection. Microb Genom. 2018 Mar;4(3):e000165. doi: 10.1099/mgen.0.000165. Epub 2018 Mar 16. PMID: 29547094; PMCID: PMC5885017.
AdeB[A551T, T584I, T606A, D642A, T645S,P660Q , L730F, A768T]	Huys G, Cnockaert M, Nemec A, Swings J. Sequence-based typing of ade B as a potential tool to identify intraspecific groups among clinical strains of multidrug-resistant Acinetobacter baumannii. J Clin Microbiol. 2005 Oct;43(10):5327-31. doi: 10.1128/JCM.43.10.5327-5331.2005. PMID: 16208010; PMCID: PMC1248495.
OprD[T98A, D278G]	Yang H, Huang L, Barnie PA, Su Z, Mi Z, Chen J, Aparna V, Kumar D, Xu H. Characterization and distribution of drug resistance associated β-lactamase, membrane porin and efflux pump genes in MDR A. baumannii isolated from Zhenjiang, China. Int J Clin Exp Med. 2015 Sep 15;8(9):15393-402. PMID: 26629028; PMCID: PMC4658917.
AdeR[Lys219Glu]	Yang H, Huang L, Barnie PA, Su Z, Mi Z, Chen J, Aparna V, Kumar D, Xu H. Characterization and distribution of drug resistance associated β-lactamase,

	membrane porin and efflux pump genes in MDR A. baumannii isolated from Zhenjiang, China. Int J Clin Exp Med. 2015 Sep 15;8(9):15393-402. PMID: 26629028; PMCID: PMC4658917.
AdeR[Tyr31Phe, Val136Ala ,Leu142Ile, Asn115His]	Ali Riza Atasoy, Ihsan Hakki Ciftci, Mustafa Petek & Huseyin Agah Terzi (2016) Investigation of mutations in adeR and adeS gene regions in gentamicine resistant Acinetobacter baumannii isolates, Biotechnology & Biotechnological Equipment, 30:2, 360-367, DOI: 10.1080/13102818.2015.1135082
AdeS[Met197 Ile, Gly200 Cys]	Tein-Yao Chang, Bo-Jun Huang, Jun-Ren Sun, Cherng-Lih Perng, Ming-Chin Chan, Cheng-Ping Yu, Tzong-Shi Chiueh, AdeR protein regulates adeABC expression by binding to a direct-repeat motif in the intercistronic spacer,Microbiological Research,Volume 183,2016,Pages 60-67,ISSN 0944-5013,https://doi.org/10.1016/j.micres.2015.11.010.
AdeR[Met197Ile, Ser200Cys, Glu219Ala]	Tein-Yao Chang, Bo-Jun Huang, Jun-Ren Sun, Cherng-Lih Perng, Ming-Chin Chan, Cheng-Ping Yu, Tzong-Shi Chiueh, AdeR protein regulates adeABC expression by binding to a direct-repeat motif in the intercistronic spacer,Microbiological Research,Volume 183,2016,Pages 60-67,ISSN 0944-5013,https://doi.org/10.1016/j.micres.2015.11.010.
AdeS[Gly186Val]	Sun JR, Perng CL, Lin JC, Yang YS, Chan MC, Chang TY, Lin FM, Chiueh TS. AdeRS combination codes differentiate the response to efflux pump inhibitors in tigecycline-resistant isolates of extensively drug-resistant Acinetobacter baumannii. Eur J Clin Microbiol Infect Dis. 2014 Dec;33(12):2141-7. doi: 10.1007/s10096-014-2179-7. Epub 2014 Jun 18. PMID: 24939621.
AdeR[Ala136Val]	Sun JR, Perng CL, Lin JC, Yang YS, Chan MC, Chang TY, Lin FM, Chiueh TS. AdeRS combination codes differentiate the response to efflux pump inhibitors in tigecycline-resistant isolates of extensively drug-resistant Acinetobacter baumannii. Eur J Clin Microbiol Infect Dis. 2014 Dec;33(12):2141-7. doi: 10.1007/s10096-014-2179-7. Epub 2014 Jun 18. PMID: 24939621.
AdeS[A91V, A94V, R108G, L122P, A130V/T, L133P, Q141R, T153A/M, D167A/G, S188F, E201G]	Hammerstrom TG, Beabout K, Clements TP, Saxer G, Shamoo Y (2015) Acinetobacter baumannii Repeatedly Evolves a Hypermutator Phenotype in Response to Tigecycline That Effectively Surveys Evolutionary Trajectories to Resistance. PLoS ONE 10(10): e0140489. doi:10.1371/journal.pone.0140489
AdeS[R152K]	Yoon E-J, Balloy V, Fiette L, Chignard M, Courvalin P, Grillot-Courvalin C. 2016. Contribution of the Ade resistance-nodulation-cell division-type efflux pumps to fitness and pathogenesis of Acinetobacter baumannii. mBio 7(3):e00697-16. doi:10.1128/mBio.00697-16.
AdeR[A91V]	Yoon E-J, Balloy V, Fiette L, Chignard M, Courvalin P, Grillot-Courvalin C. 2016. Contribution of the Ade resistance-nodulation-cell division-type efflux pumps to fitness and pathogenesis of Acinetobacter baumannii. mBio 7(3):e00697-16. doi:10.1128/mBio.00697-16.
AdeL[N334H]	Yoon E-J, Balloy V, Fiette L, Chignard M, Courvalin P, Grillot-Courvalin C. 2016. Contribution of the Ade resistance-nodulation-cell division-type efflux pumps to fitness and pathogenesis of Acinetobacter baumannii. mBio 7(3):e00697-16. doi:10.1128/mBio.00697-16.
AdeN[delta C584]	Yoon E-J, Balloy V, Fiette L, Chignard M, Courvalin P, Grillot-Courvalin C. 2016. Contribution of the Ade resistance-nodulation-cell division-type efflux pumps to

	fitness and pathogenesis of Acinetobacter baumannii. mBio 7(3):e00697-16. doi:10.1128/mBio.00697-16.
AdeS[G186V]	D'Souza R, Pinto NA, Phuong NL, Higgins PG, Vu TN, Byun J-H, Cho YL, Choi JR and Yong D (2019) Phenotypic and Genotypic Characterization of Acinetobacter spp. Panel Strains: A Cornerstone to Facilitate Antimicrobial Development. Front. Microbiol. 10:559. doi: 10.3389/fmicb.2019.00559
AdeR[A136V]	D'Souza R, Pinto NA, Phuong NL, Higgins PG, Vu TN, Byun J-H, Cho YL, Choi JR and Yong D (2019) Phenotypic and Genotypic Characterization of Acinetobacter spp. Panel Strains: A Cornerstone to Facilitate Antimicrobial Development. Front. Microbiol. 10:559. doi: 10.3389/fmicb.2019.00559
AdeS[A94V, L172P, F215L, and Q281D]	Leus, IV, Adamiak, J, Trinh, AN, et al. Inactivation of AdeABC and AdeIJK efflux pumps elicits specific nonoverlapping transcriptional and phenotypic responses in Acinetobacter baumannii. Mol Microbiol. 2020; 114: 1049– 1065. https://doi.org/10.1111/mmi.14594
PBP3[A515V, A515T, A583V, T526S, T506P, T512S]	Nelson K, Rubio-Aparicio D, Tsivkovski R, Sun D, Totrov M, Dudley M, Lomovskaya O. In Vitro Activity of the Ultra-Broad-Spectrum Beta-Lactamase Inhibitor QPX7728 in Combination with Meropenem against Clinical Isolates of Carbapenem-Resistant Acinetobacter baumannii. Antimicrob Agents Chemother. 2020 Oct 20;64(11):e01406-20. doi: 10.1128/AAC.01406-20. PMID: 32868334; PMCID: PMC7577151.
PBP3[H370Y]	Hawkey J, Ascher DB, Judd LM, Wick RR, Kostoulias X, Cleland H, Spelman DW, Padiglione A, Peleg AY, Holt KE. Evolution of carbapenem resistance in Acinetobacter baumannii during a prolonged infection. Microb Genom. 2018 Mar;4(3):e000165. doi: 10.1099/mgen.0.000165. Epub 2018 Mar 16. PMID: 29547094; PMCID: PMC5885017.
**PBP1a (ponA)**	Cayô R, Rodríguez MC, Espinal P, Fernández-Cuenca F, Ocampo-Sosa AA, Pascual A, Ayala JA, Vila J, Martínez-Martínez L. Analysis of genes encoding penicillin-binding proteins in clinical isolates of Acinetobacter baumannii. Antimicrob Agents Chemother. 2011 Dec;55(12):5907-13. doi: 10.1128/AAC.00459-11. Epub 2011 Sep 26. PMID: 21947403; PMCID: PMC3232777.
A_{244}T (C), S_{382}N (C), T_{636}A (C, D), T_{636}A (E, J), T38A (H), A_{244}T (J), S_{382}N (J)	
L_{147}I (I, B), A_{244}T (G, H), S_{382}N (G, H), T_{636}A (G, H)	
**PBP1b (mrdA)**	Cayô R, Rodríguez MC, Espinal P, Fernández-Cuenca F, Ocampo-Sosa AA, Pascual A, Ayala JA, Vila J, Martínez-Martínez L. Analysis of genes encoding penicillin-binding proteins in clinical isolates of Acinetobacter baumannii. Antimicrob Agents Chemother. 2011 Dec;55(12):5907-13. doi: 10.1128/AAC.00459-11. Epub 2011 Sep 26. PMID: 21947403; PMCID: PMC3232777.
P_{112}S (A, I, B), P_{764}S (G, H,C,J)	
**PBP2 (pbpA)**	Cayô R, Rodríguez MC, Espinal P, Fernández-Cuenca F, Ocampo-Sosa AA, Pascual A, Ayala JA, Vila J, Martínez-Martínez L. Analysis of genes encoding penicillin-binding proteins in clinical isolates of Acinetobacter baumannii. Antimicrob Agents Chemother. 2011 Dec;55(12):5907-13. doi: 10.1128/AAC.00459-11. Epub 2011 Sep 26. PMID: 21947403; PMCID: PMC3232777.
V_{509}I (C, G, J), E_{110}Q (D, K), P_{665}A (E)	
**PBP3 (fstl)**	Cayô R, Rodríguez MC, Espinal P, Fernández-Cuenca F, Ocampo-Sosa AA, Pascual A, Ayala JA, Vila J, Martínez-Martínez L. Analysis of genes encoding penicillin-

binding proteins in clinical isolates of Acinetobacter baumannii. Antimicrob Agents Chemother. 2011 Dec;55(12):5907-13. doi: 10.1128/AAC.00459-11. Epub 2011 Sep 26. PMID: 21947403; PMCID: PMC3232777.

$G_{523}V$ (G, C, J), $H_{370}Y$ (I)

Cayô R, Rodríguez MC, Espinal P, Fernández-Cuenca F, Ocampo-Sosa AA, Pascual A, Ayala JA, Vila J, Martínez-Martínez L. Analysis of genes encoding penicillin-binding proteins in clinical isolates of Acinetobacter baumannii. Antimicrob Agents Chemother. 2011 Dec;55(12):5907-13. doi: 10.1128/AAC.00459-11. Epub 2011 Sep 26. PMID: 21947403; PMCID: PMC3232777.

PBP5/6 (dacC)

$N_{329}S$ (A, C, D, I, B)

$T_{374}V$ (B, E, A), $N_{296}D$ (B, E, A), $N_{307}S$ (B, E, A)

Cayô R, Rodríguez MC, Espinal P, Fernández-Cuenca F, Ocampo-Sosa AA, Pascual A, Ayala JA, Vila J, Martínez-Martínez L. Analysis of genes encoding penicillin-binding proteins in clinical isolates of Acinetobacter baumannii. Antimicrob Agents Chemother. 2011 Dec;55(12):5907-13. doi: 10.1128/AAC.00459-11. Epub 2011 Sep 26. PMID: 21947403; PMCID: PMC3232777.

PBP6b (dacD)

$P_{28}S$ (A, I, B), $A_{277}T$ (D, K), $T_{188}P$ (G, H, B), $V_{350}I$ (K, D), $S_{429}N$ (K, D)

Cayô R, Rodríguez MC, Espinal P, Fernández-Cuenca F, Ocampo-Sosa AA, Pascual A, Ayala JA, Vila J, Martínez-Martínez L. Analysis of genes encoding penicillin-binding proteins in clinical isolates of Acinetobacter baumannii. Antimicrob Agents Chemother. 2011 Dec;55(12):5907-13. doi: 10.1128/AAC.00459-11. Epub 2011 Sep 26. PMID: 21947403; PMCID: PMC3232777.

PBP7/8 (pbpG)

$T_{45}S$ (A, C, D), $A_{84}T$ (A, I, B), $T_{39}I$ (I), $T_{45}S$ (G, H, I), $T_{45}S$ (B, F, J, K)

Cayô R, Rodríguez MC, Espinal P, Fernández-Cuenca F, Ocampo-Sosa AA, Pascual A, Ayala JA, Vila J, Martínez-Martínez L. Analysis of genes encoding penicillin-binding proteins in clinical isolates of Acinetobacter baumannii. Antimicrob Agents Chemother. 2011 Dec;55(12):5907-13. doi: 10.1128/AAC.00459-11. Epub 2011 Sep 26. PMID: 21947403; PMCID: PMC3232777.

MtgA (mtgA)

$F_{18}L$ (A, C, D), $T_{49}P$ (A, C, D), $N_{179}S$ (A, C, D)

$F_{18}L$ (G, H, I), $Q_{100}E$ (G, H, C, J), $T_{49}P$ (G, I), $N_{179}S$ (G, H, I)

$F_{18}L$ (B, E, F), $T_{49}P$ (B, E, F), $I_{54}V$ (E, F), $N_{179}S$ (B, E, F)

$F_{18}L$ (J, K), $T_{49}P$ (J, K), $N_{179}S$ (J, K)

S3 Table: FASTA sequences and corresponding gene IDs of studied gene variants

**Gene variant**	**Gene variant ID**
Omp33_V2	|470.1561.peg.274|ASZ73_01405|___33-36_kDa_outer_membrane_protein___Acinetobacter_baumannii_strain_MRSN4119_|_470.1561
**Sequence**	MKKLGLATAVLLAMTGAHAYQFEVQGQSEYVDTTANDKNFTGTAQGTYYFKNVDASKGPLAEAAFLNQASNVSVAYNYIKYDEKDTVNVESHTYGVKGEAYLPTPYLPVYASASYNHTINDFKDGVSDDNGDRYALEAGAMLLPNFLVAVGYTSVADQISLDAFGVNKYGIAKAVGESVAIDEKQDAVTARTK

	YVGNIDGTNMAIGFEAFGVFAEDNAYGMKTDLFVTPKLSVGASFADVSAFNSGYDHVWGGHTQYFITPAVAVGADFVKANAKDGNPRDTQTIGLNAKFRF
Omp33_V3	|470.7597.peg.5426|___33-36_kDa_outer_membrane_protein___Acinetobacter_baumannii_strain_MRSN7088_|_470.7597
**Sequence**	HAYQFEVQGQSEYVDTTANDKNFTGDVAGTFYLKNVDTAKGPLAEAAFLNQASSVSLGYSYQQYDQNNVNYHIGTYGVKGEAYVPTPYLPVYASATYNHTDVDGKNNNSKDDNGDRYALEVGAMLLPNFLMTVGYTSVANQFALDNFGIIGNGIYSAVNQTAAIQNDQDAVTARAKYVGPIDGTNMAIGFEAAGAFGQENQYGLKTDLYLTPKLSVGATFVGNDGSADIKGNDLGEFRQAWGGNVNYFITPALAVGASYMKADVKNASTSYDTQTIGLNTKFRF
OmpA_V1	|470.1305.peg.1410|___Outer_membrane_protein_A_precursor___Acinetobacter_baumannii_strain_2011SDAB2_|_470.1305
**Sequence**	MKLSRIALATMLVAAPLAAANAGVTVTPLLLGYTFQDSQHNNGGKDGNLTNSPELQDDLFVGAALGIELTPWLGFEAEYNQVKGDVDGASAGAEYKQKQINGNFYVTSDLITKNYDSKIKPYVLLGAGHYKYDFDGVNRGTRGTSEEGTLGNAGVGAFWRLNDALSLRTEARATYNADEEFWNYTALAGLNVVLGGHLKPAAPVVEVAPVEPTPVAPQPQELTEDLNMELRVFFDTNKSNIKDQYKPEIAKVAEKLSEYPNATARIEGHTDNTGPRKLNERLSLARANSVKSALVNEYNVDASRLSTQGFAWDQPIADNKTKEGRAMNRRVFATITGSRTVVVQPGQEAAAPAAAQ
OmpA_V2	|470.2142.peg.2664|___Outer_membrane_protein_A_precursor___Acinetobacter_baumannii_A069_|_470.2142
**Sequence**	MKLSRIALATMLVAAPLAAANAGVTVTPLLLGYTWQDSEHNNNKLTDHAELQDDLFVGAGLGVELTPWLGFEAEYNQVKGDLDGTGVQGAEYKQKTIAGNFYATSDLITKNYDSKFKPYVLLGAGQTKTEFDGIYEDKKDTIGNAGVGAFYRLNDALSLRTEARGTYDFDEKYWRYTALAGLNVVLGGHLKPAAPVVEVAPVEPTPVAPQPQELTEDLNMELRVFFDTNKSNIKDQYKPEIAKVAEKLSEYPNATARIEGHTDNTGPRKLNERLSLARANSVKSALVNEYNVDASRLSTQGFAWDQPIADNKTKEGRAMNRRVFATITGSRTVVVQPGQQAQ
OprD_ V2	|470.7872.peg.522|___Outer_membrane_low_permeability_porin_OprD_family___Acinetobacter_baumannii_strain_MRSN7482_|_470.7872
**Sequence**	MLKAQKLTLAVLISAAIISSAQASEQSEAKGFVEDANGSILFRTGYISRDKKDGRADNSSFAQTAIVNIDSGFTPGIVGFGVGVVGDGSFKIGENKNAGNNMIPQHNDGSAYDHWARGGANVKARFSNTTVRYGTQVLDLPVLASNTARLVPEYFTGTLLTSHEIKDLEVVAGKFTKNQYSDQIATDQNGLDRAVVWGAKYKFDDQISGSYYGVDVKDKLDRHYVNVNYKQPLANDSSLTYDFSGYHTKFDKGANLSYATGPADEDKTNNIWAISGTYATGPHSVMLAYQQNSGNIGYNYGVNQDGGQSVYLPNSYLSDFIGNDEKSAQIQYSLDFGKLGVLPGLNWTTAYVYGWDIKTSNGADDSNESEFFNQVKYTVQSGFAKGSSLRLRNSIYRADNAYTTDYMPDTNEWRIFLDIPVTLF
OprD_ V3	|470.7769.peg.2039|___Outer_membrane_low_permeability_porin_OprD_family___Acinetobacter_baumannii_strain_MRSN7345_|_470.7769
**Sequence**	MLKAQKLTLAVLISAAIISSAQASEQSEAKGFVEDANGSILFRTGYISRDKKNGVDDTSSFAQTAIVNIESGFTPGIVGFGVGVVGDGSFKIGANKNAGNNMIPRETLNGKENSGDSYDHWARGGGSVKARFSNTTVRYGTQVLDLPVLASNTARLVPEYFTGTLLTSHEIKDLEVIAGKFTKDQYSDQVNTDGRHLDRAIVWGAKYKFDDNLNASYYGLDSKDKLERHYLNVNYKQPLANDSSLTYDFSGYHTKFDEGASTYSQTTDDLSNRKNNIWAISTAYNTGPHNIMVAYQQNSGNVGYDYGENADGGQSIYLPNSYLSDFIGNDEKSAQIQYSLDFGKLGVLPGLNWTTAFVYGWDIKVKGLTDDAEEREFFNQVKYTVQSGFAKDASLRIRNSYYRASNAYQTNAYIGDTNEWRIFLDIPVKLF
