# Supplementary material for: Global genomic epidemiology of chromosomally mediated non-enzymatic carbapenem resistance in Acinetobacter baumannii: on the way to predict and modify resistance
Source: Front Microbiol. 2023 Oct 6;14:1271733. doi: 10.3389/fmicb.2023.1271733 (PMC10587612; doi:10.3389/fmicb.2023.1271733)

Supplementary Figures

Global genomic epidemiology of chromosomally mediated non-enzymatic carbapenem resistance in *Acinetobacter baumannii* : On the way to predict and modify resistance

Wedad M. Nageeb [1*], Nada AlHarbi [2], Amani A. Alrehaili [3] , Shadi A. Zakai [4] , Ahmed Elfadadny [5] , Helal F. Hetta [6]

[1] Department of Medical Microbiology and Immunology, Faculty of Medicine, Suez Canal University, Ismailia, Egypt
[2] Department of Biology, Princess Nourah bint Abdulrahman University, College of Science, Riyadh, Saudi Arabia

[3] Department of Clinical Laboratory Sciences, College of Applied Medical Sciences, Taif University, Taif, Saudi Arabia

[4] Department of Clinical Microbiology and Immunology, Faculty of Medicine, King Abdulaziz University, Jeddah, Saudi Arabia

[5] Department of Animal Internal Medicine, Faculty of Veterinary Medicine, Damanhour University, Damanhour, El-Beheira, Egypt

[6] Department of Medical Microbiology and Immunology, Faculty of Medicine , Assiut University, Assiut, Egypt

*** Correspondence:**
Wedad M. Nageeb
wedad_saleh@med.suez.edu.eg

S1A Fig. Structure of AdeA Membrane Fusion Protein (MFP) as predicted using AlphaFold

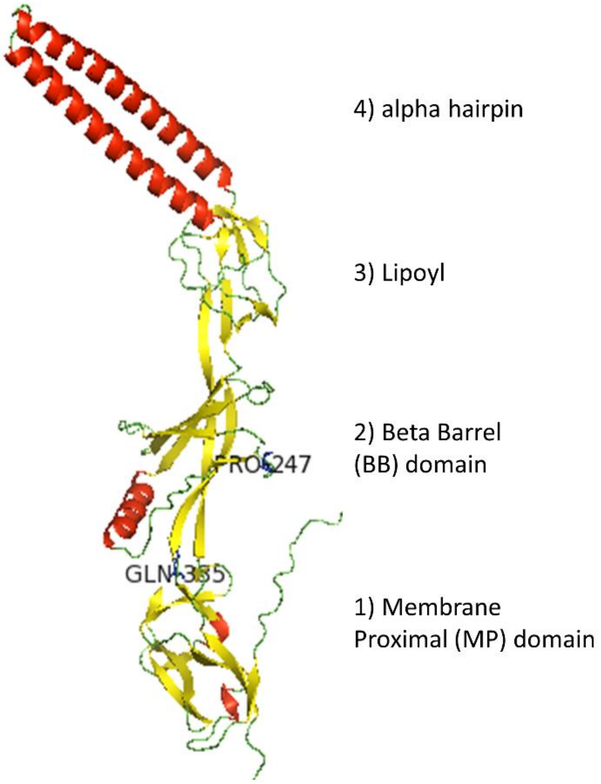

A typical membrane fusion protein is formed of 4 domains : 1) Membrane Proximal (MP) domain 2) Beta Barrel (BB) domain 3) Lipoyl 4) alpha hairpin ordered inside out (from the inner membrane spanning the periplasm to the outer membrane channel)

S1B Fig. Position of AdeA Q335H

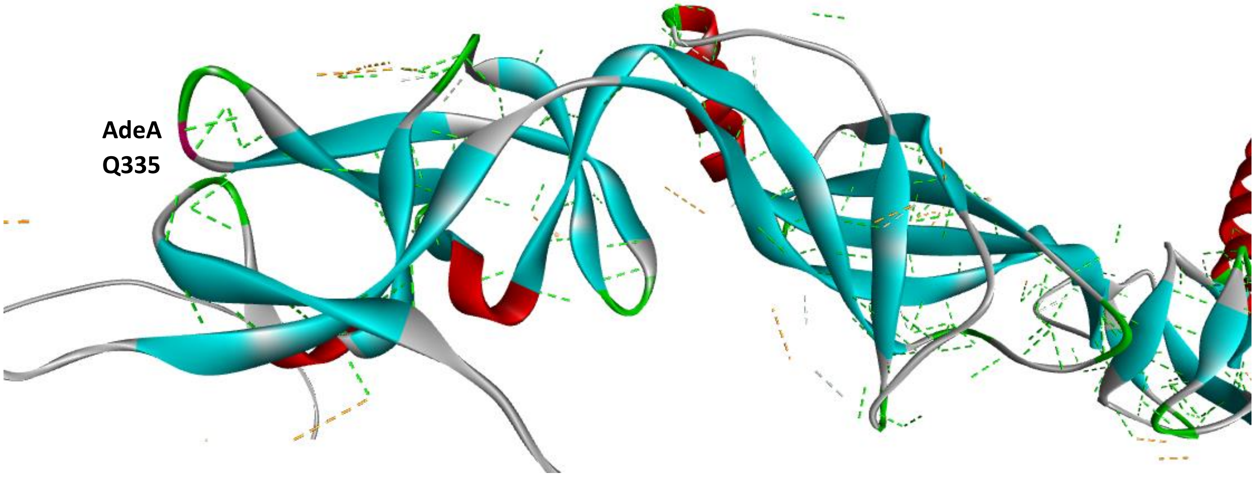

S1C Fig. Position of AdeA P247A

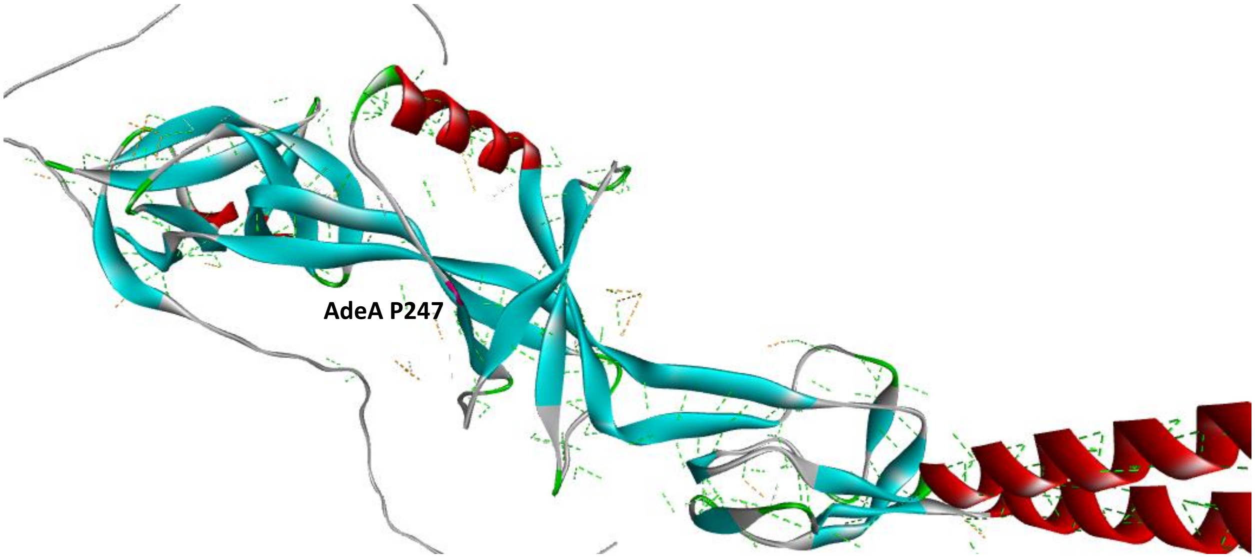

S2 Fig. AdeF S172F located at the lower side of the α-helical hairpin that spans the periplasm and is tightly bonded to surrounding residues

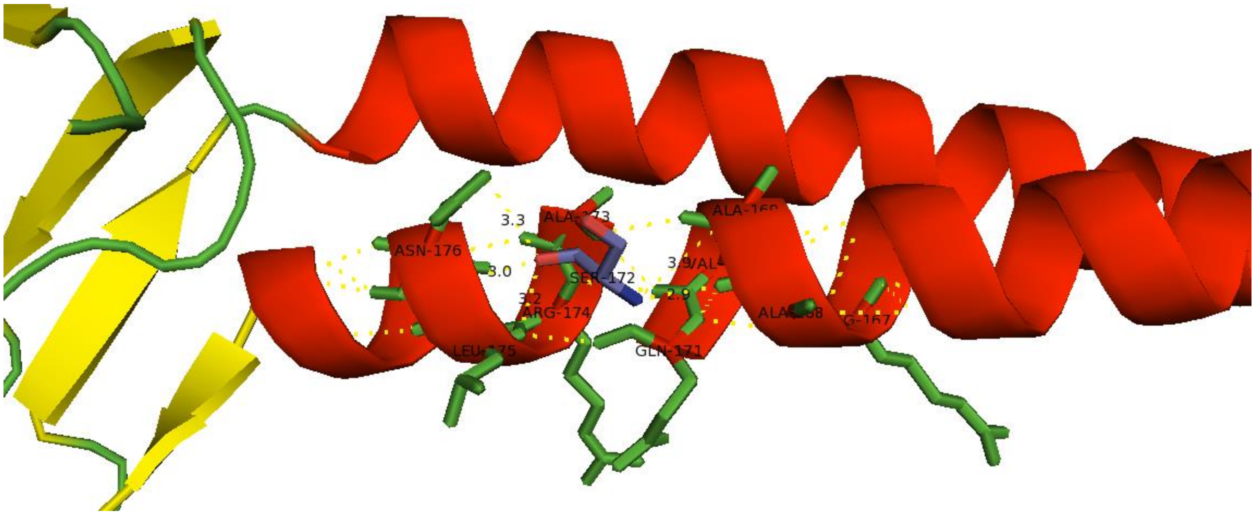

S3 Fig. Position of significant variants observed in AdeB in carbapenem susceptible and resistant groups

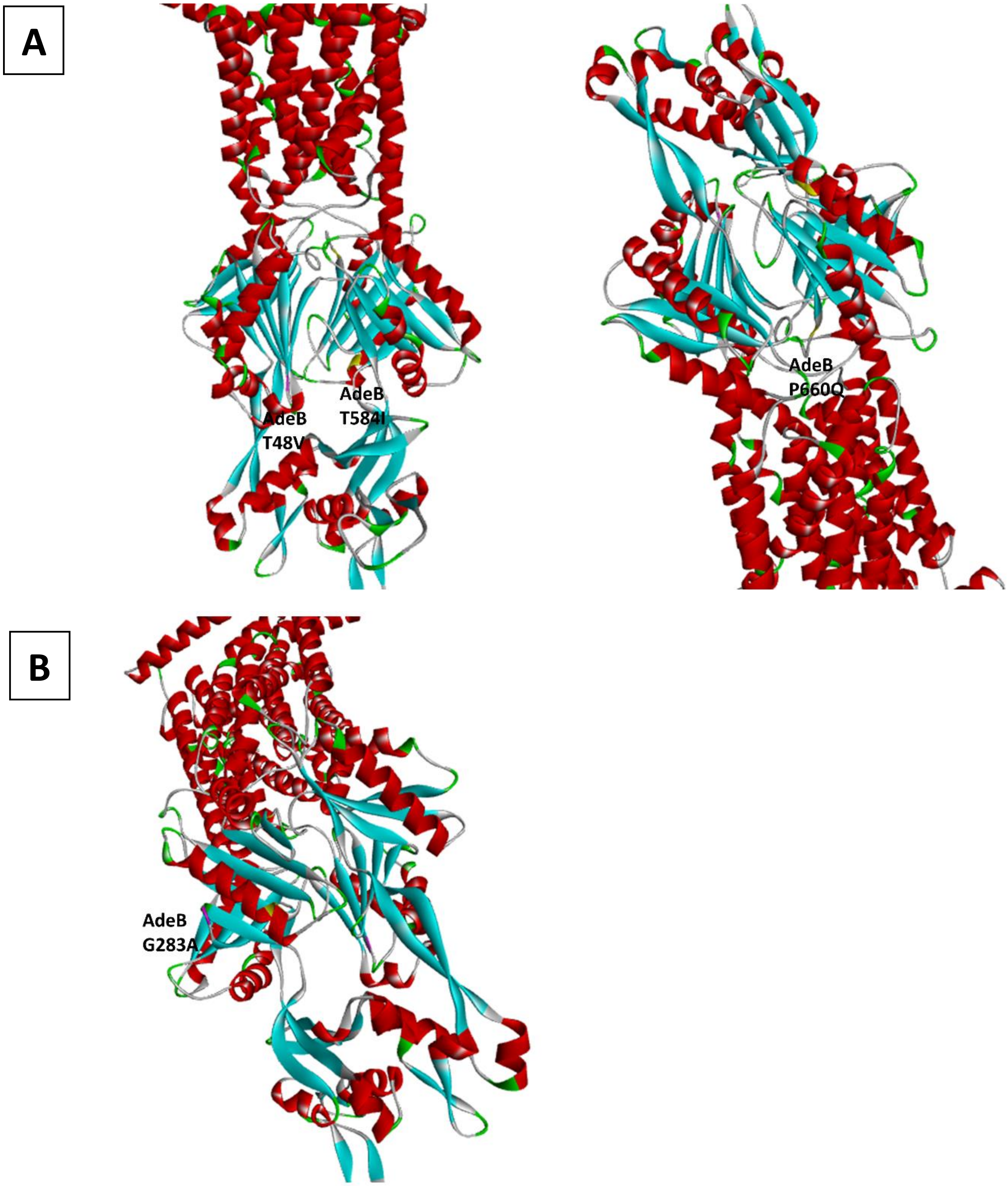

S4 Fig. AdeJ V573M mapped to the AdeJ efflux pump structure available at PDB (7M4Q)

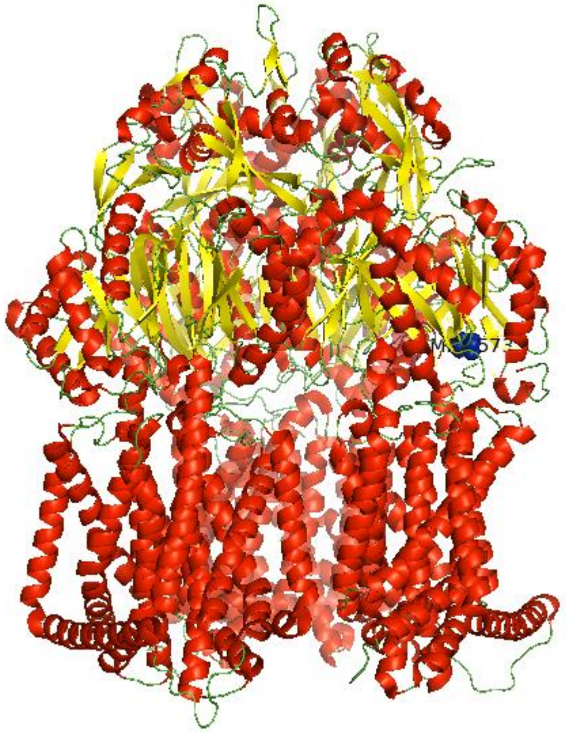

S5 Fig. AdeG A350T and AdeG D501N variants mapped to the AdeG AlphaFold predicted 3D structure

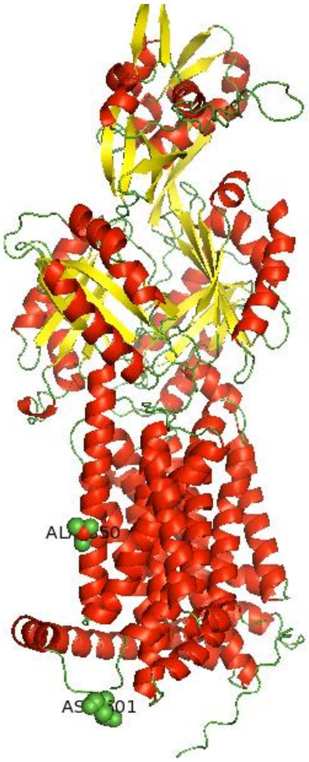

S6 Fig. Position of variants mapped to the AdeC AlphaFold predicted 3D structure

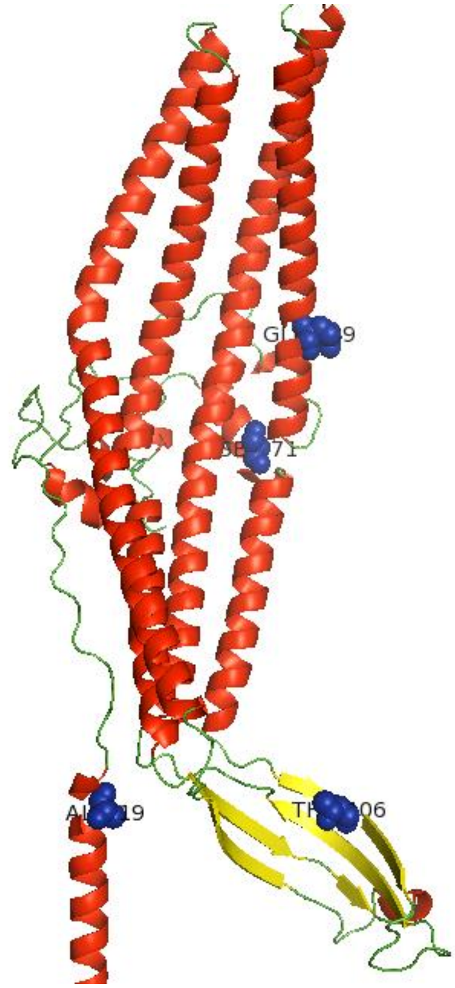

S7 Fig. AdeH R100G and AdeH A289G mapped to AlphaFold predicted 3D structure

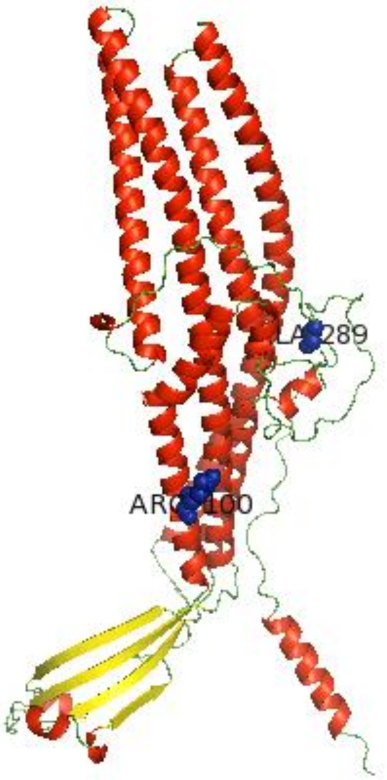

S8 Fig. Significant variants mapped to AlphaFold predicted AdeS 3D structure

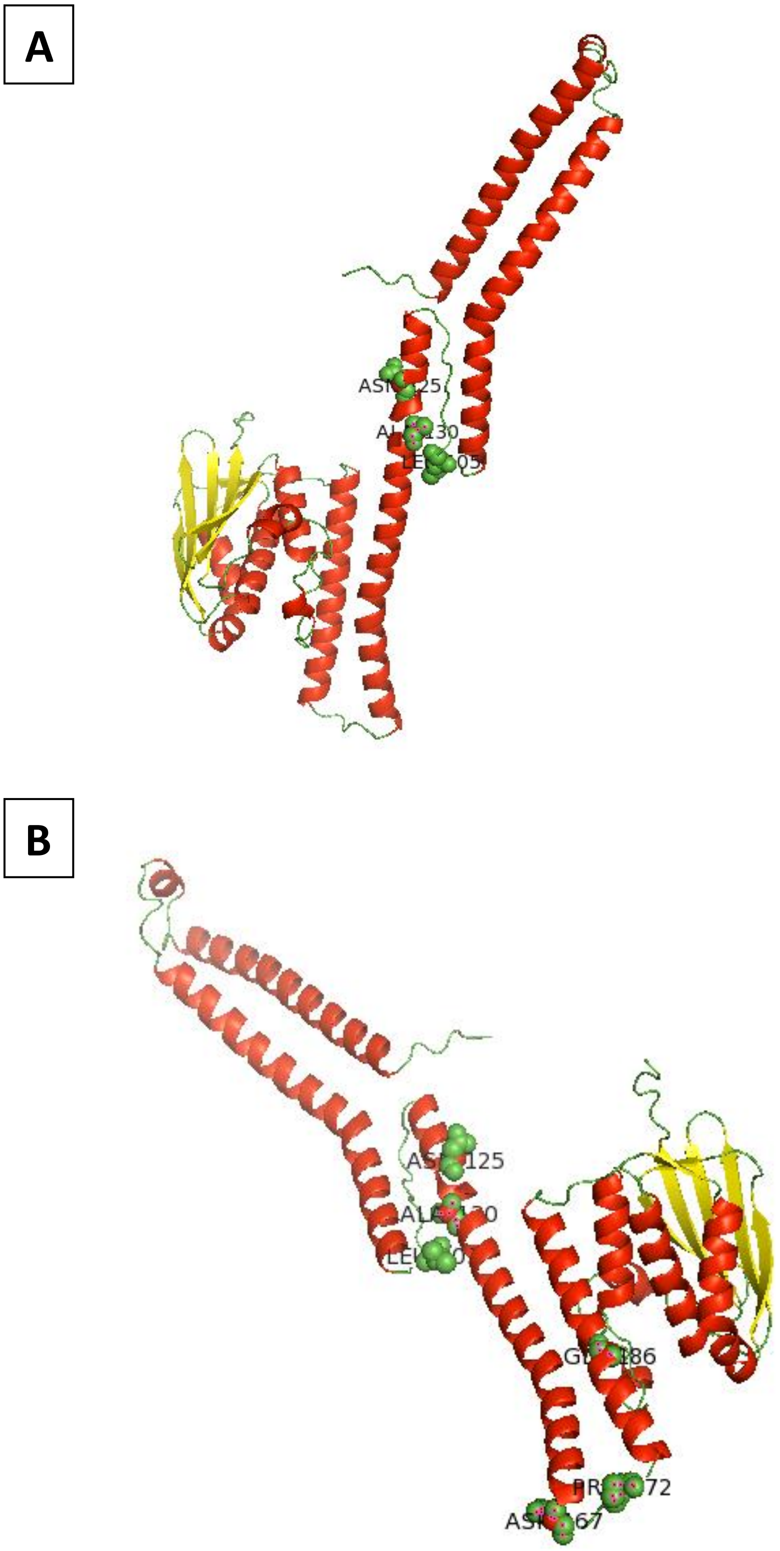

S9A Fig. Variants related to the DNA binding pocket of AdeR 3D structure (5X5L)

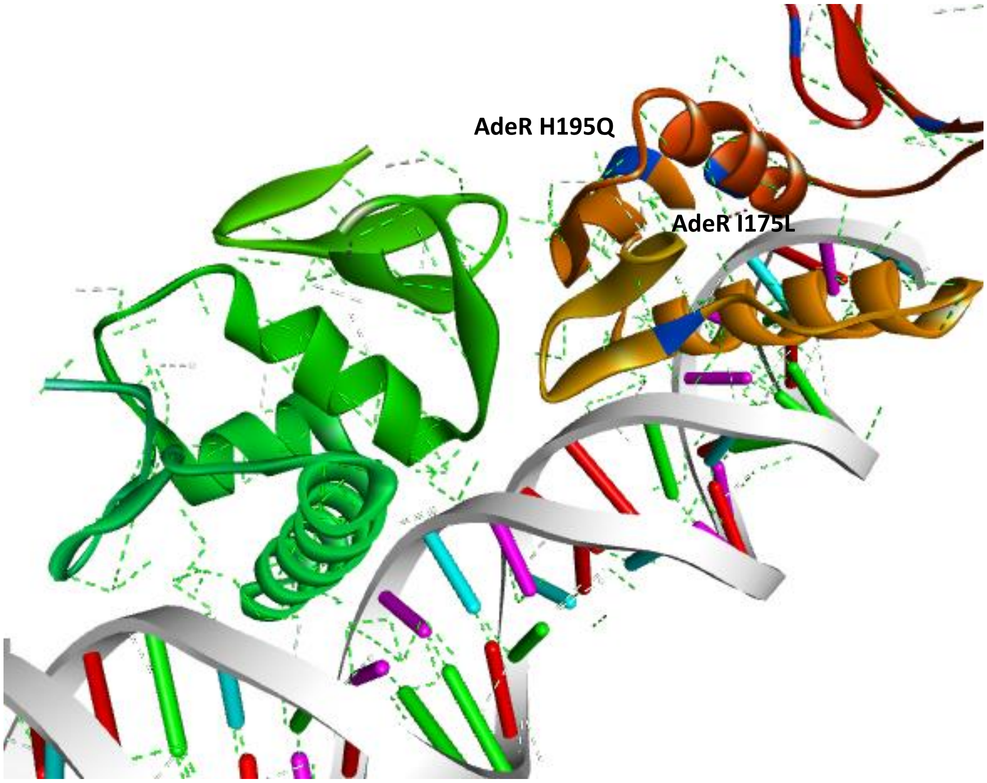

S9B Fig. AdeR A53V bonding with Ile 49

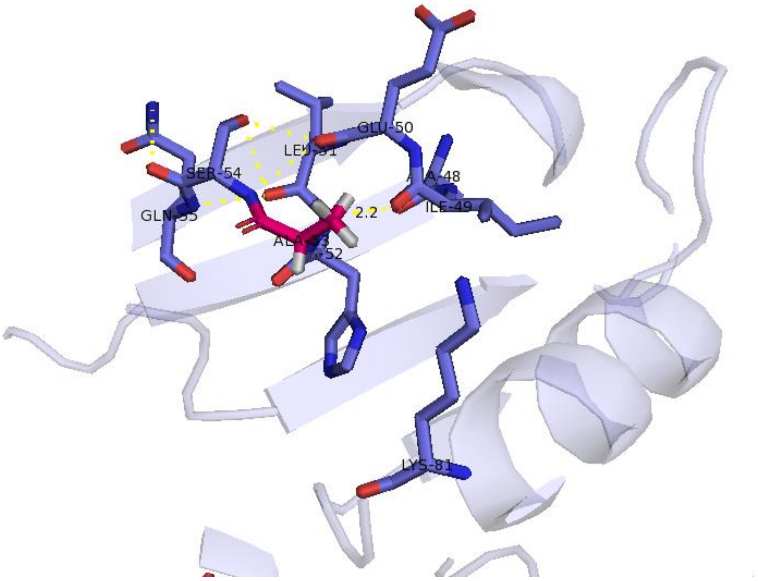

S10A Fig. Variants located in between C-terminal DNA binding domain and N-terminal response regulator domain mapped to AdeR 3D structure (5X5L)

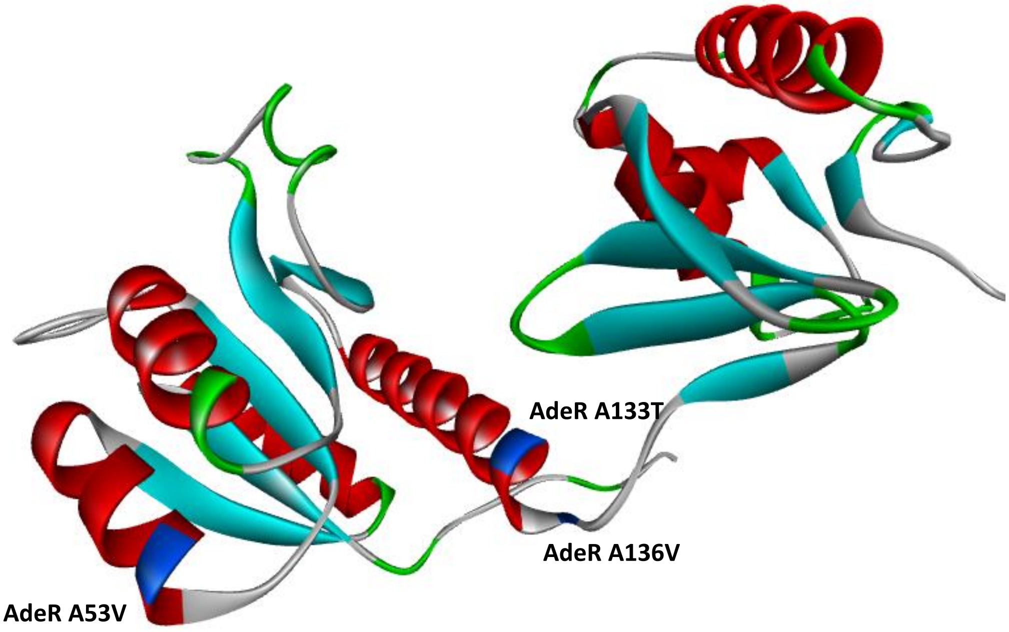

S10B Fig. Bonding of mutated AdeR A133 and A136

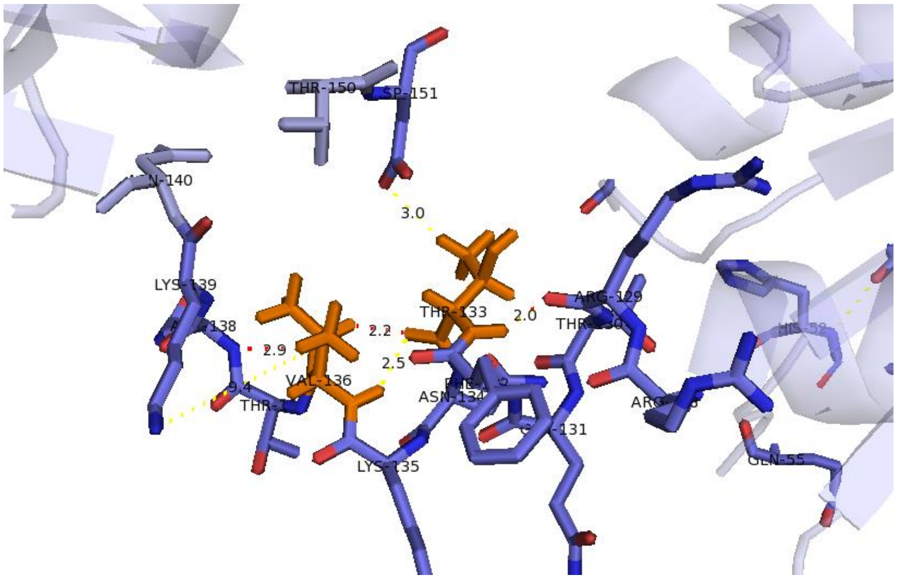

S11 Fig. Significant variants located at outer membrane porin OprB

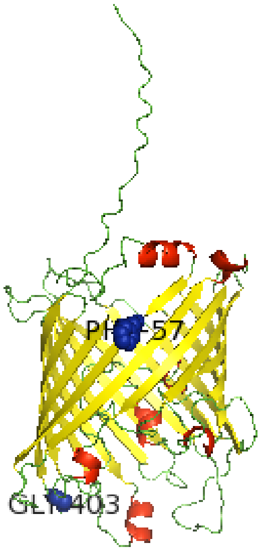

S12 Fig. Significant variants located at the PBP-*mtg*A

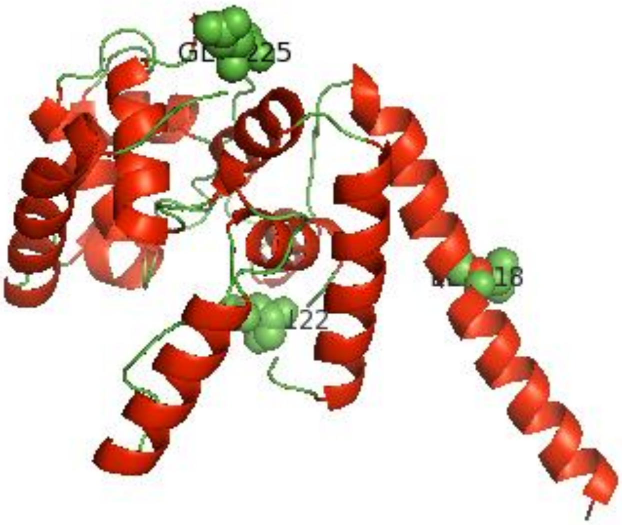

Supplement: Supplementary file 2 [file Data_Sheet_2.pdf]
